# Novel Ag/Ni bimetallic-loaded sawdust-derived biochar as a periodate activator for the effective degradation of levofloxacin

**DOI:** 10.1039/d6ra06933a

**Published:** 2026-09-14

**Authors:** Abdullah S. Bostaji, Nasr Rashid, Bandar Alwushayh, Abdelhalim Azam, Karim Amer

**Affiliations:** a Department of Civil Engineering, College of Engineering and Architecture, Umm Al-Qura University Makkah 24381 Saudi Arabia asbostaji@uqu.edu.sa; b Department of Electrical Engineering, College of Engineering, Jouf University Sakaka 72388 Saudi Arabia nasrrashid@ju.edu.sa; c Department of Civil Engineering, College of Engineering, Jouf University Sakaka 72388 Saudi Arabia baalwushayh@ju.edu.sa amazam@ju.edu.sa; d Technology Management Department, Egypt-Japan University of Science and Technology (E-JUST) P.O. Box 179, New Borg El-Arab City Postal Code 21934 Alexandria Egypt karim.amer@ejust.edu.eg

## Abstract

In this study, a novel silver/nickel-loaded sawdust-derived biochar (Ag/Ni-SDBC) was synthesized and employed as an efficient catalyst for periodate (PI) activation for the degradation of a fluoroquinolone antibiotic, namely levofloxacin (LEV), to tackle antimicrobial resistance. Although bimetallic-modified biochars have attracted increasing attention for advanced oxidation processes, their catalytic mechanisms remain poorly investigated. Moreover, to the best of our knowledge, this is the first study to investigate Ag/Ni-modified biochar for PI activation. LEV degradation by the Ag/Ni-SDBC/PI system was approximately 1.85, 1.35, and 1.15 times greater than that achieved by the SDBC/PI, Ag-SDBC/PI, and Ni-SDBC/PI systems, respectively. The optimum degradation performance was obtained at a PI concentration of 2 mM, an initial LEV concentration of 10 mg L^−1^, a catalyst dosage of 0.5 g L^−1^, and pH 7. The fabricated loaded biochar also demonstrated high reusability performance, with only an 8.79% decline in degradation efficiency after five consecutive reuse cycles. Mechanistic investigations identified singlet oxygen as the dominant reactive species, while no toxic iodinated by-products were detected. The degradation system maintained high degradation efficiency in the presence of the investigated inorganic ions, except HCO_3_^−^. Its applicability was further validated in various water matrices, including real domestic and pharmaceutical wastewater, achieving total organic carbon mineralization efficiencies of 40.26% and 32.48%, respectively. A cost study revealed the economic feasibility of the system, yielding a positive net present value and a payback period of 4.3 years. Overall, the Ag/Ni-SDBC/PI system represents an efficient, reusable, and economically viable technology that can be promising for industrial wastewater treatment.

## Abstract 2

A novel silver/nickel-loaded sawdust-derived biochar (Ag/Ni-SDBC) was synthesized and employed as an efficient catalyst for periodate (PI) activation for the degradation of levofloxacin.

## Graphical abstract

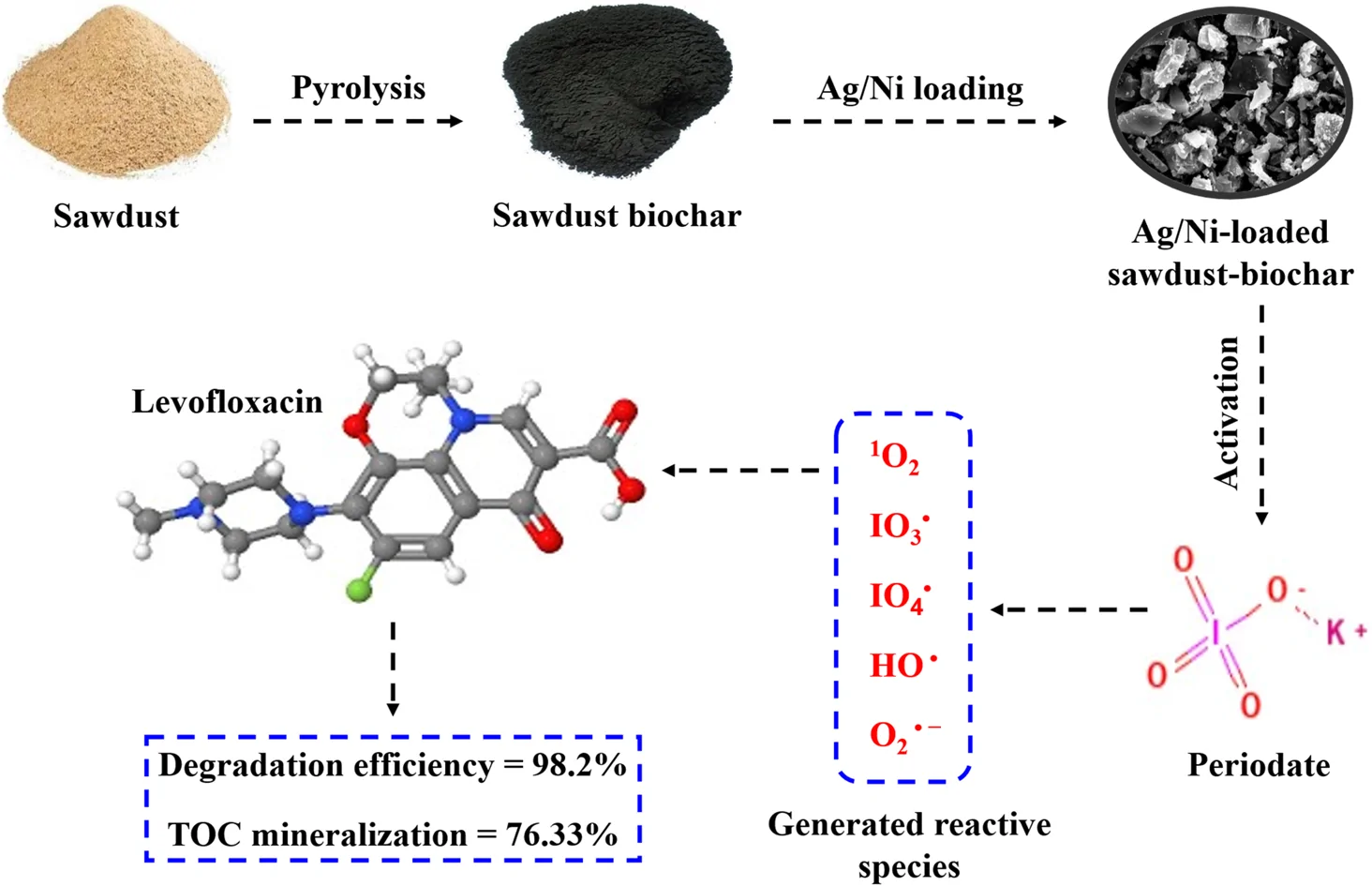

## 1. Introduction

Antibiotics have been excessively utilized by humans and animals for treating bacterial infections, leading to their accumulation in the environment.^1^ Levofloxacin (LEV), a fluoroquinolone antibiotic, has been pervasively employed for curing infectious diseases owing to its effectiveness towards a wide spectrum of the bacterial community.^2^ The incomplete metabolism of LEV in living bodies has resulted in the recurring release of LEV residues into the environment *via* urine or/and feces, thereby participating in the escalation of antibacterial resistance.^3^ Further, LEV has been uncovered in surface water, groundwater, and drinking water due to the discharge of LEV-laden wastewater from different sources, such as healthcare systems and pharmaceutical production industries, which jeopardizes human health and aquatic systems.^4^ Conventional treatment approaches (*e.g.*, adsorption, membrane separation, activated sludge, ion exchange) possess serious shortcomings that limit their scalable applicability, such as high cost, extended reaction time, and suboptimal performance.^5–7^ Thus, finding an efficient, inexpensive technology for treating antibiotics is of great priority to conserve water environment quality.

Advanced oxidation processes (AOPs) have been recognized for their potential to effectively degrade stubborn organic pollutants (*e.g.*, antibiotics) *via* the generated reactive oxygen species (ROS).^8^ Among the AOPs, periodate (PI, IO_4_^−^) has been employed as an inorganic oxidant with remarkable reactivity, noticeable stability, and limited risks during transportation and storage in comparison with other oxidants such as persulfate (PS), peroxymonosulfate (PMS), and hydrogen peroxide (H_2_O_2_).^9^ However, the oxidation capability of sole PI is inadequate for the efficacious degradation of emerging pollutants.^10^ Therefore, activation of PI is pivotal for generating reactive iodine species (RIS) such as iodyl radicals 
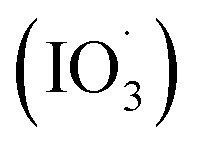
 and periodyl radicals 
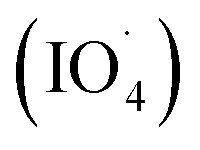
 and ROS such as hydroxyl radicals (HO˙), superoxide radicals (O_2_˙^−^) and singlet oxygen (^1^O_2_).^11^

Activation of PI can be executed by various approaches such as light, ultrasound, and metal catalysts. However, these activation techniques encounter major limitations, as they are energy intensive and cause secondary pollution (*e.g.*, metal leaching) leading to their limited real-world application as activators of PI.^12^ Thus, employing an effective, green activator without energy consumption is indispensable for the full-scale application of PI-based AOPs.

Biochar, a carbonaceous material, with copious oxygen-containing functional groups, low-cost, and high surface area can be fabricated *via* the pyrolysis of organic biomass in an oxygen-deficient environment, thereby acting as an activator of PI.^13^ Biochar has been derived from different agricultural wastes such as rice husk, bean pods, and wheat straw.^14^ Among agricultural biomasses, sawdust is as an excellent feedstock for the fabrication of biochar due to its high lignocellulosic content, which offers abundant binding sites on the biochar's surface during the pyrolysis.^15^ Waste sawdust (SD), an agricultural residue, is generated in substantial amounts, which require resource utilization instead of dumping into landfills.^16^ Therefore, herein, sawdust was pyrolyzed into biochar to utilize in environmental applications. However, the performance of bare biochars as activators is modest due to their limited active surface sites and ineffective electron transfer potential, which necessitates modification.^17^ The catalytic and adsorption performance of intact biochar can be ameliorated *via* different methodologies such as morphology control, heteroatom doping, and metal loading.^18^ Metal loading approach has evoked considerable focus due to its competence to activate inorganic oxidants.^19^ The performance of unmodified can be enhanced in the case of monometallic-loaded biochar due to the ability of single metals to boost adsorption binding sites, raise specific surface area, offer electron donors, and activate functional groups.^20^ However, monometallic-loaded biochars suffer from metal leaching and instability.^21^ Thus, bimetallic-modified biochars can provide higher performance due to their limited metal leaching and improved electron transfer efficacy.^22^ Yu *et al.* loaded biochar with zinc and iron metals for the degradation of sulfamethoxazole antibiotic indicating that bimetallic loading exhibited better performance than that of monometallic loading.^23^ However, the degradation mechanism and pathways in bimetallic-modified biochars still need elucidation. Further, many metals have not yet been evaluated for loading onto intact biochar's surface, making this area a hot research direction. Herein, silver (Ag) and nickel (Ni) were selected to be loaded on the sawdust-derived biochar (SDBC) due to their abundance and stability.^24,25^ Further, Ag and Ni have variable oxidation states which result in redox cycling promoting electron transfer, thereby participating in reduction pathways.^26^

In this study, Ag/Ni co-loaded sawdust-derived biochar (Ag/Ni-SDBC) was fabricated for the first time. The modified biochar was then characterized to investigate the alterations in morphology, chemical composition, and chemical structure after bimetallic loading. The synthesized catalyst was utilized as an activator of PI for the degradation of LEV and other pharmaceutical compounds. Further, the degradation system was optimized, the degradation mechanism was elucidated with determining the main reactive species, and the biochar's reusability was assessed. Additionally, the effects of inorganic species and natural organic matter (NOM) on the degradation performance were explored, and the formation of toxic iodinated by-products were monitored. Moreover, the application of the degradation system to real wastewater and different water matrices was conducted to check the effectiveness of the proposed system in real-world conditions. Additionally, a techno-economic study for the degradation system was performed to evaluate its profitability.

## 2. Materials and methods

### 2.1. Raw materials and chemicals

Waste SD was obtained from a local sawmill workshop in New Borg El-Arab City, Alexandria, Egypt. All chemicals used in this study were of analytical grade and were used as received without further purification. Details of the chemicals are provided in Text S1. Stock solutions of antibiotics were freshly prepared every weak and stored in dark bottles at 4 °C. Deionized water with a resistivity of approximately 18 MΩ cm was used for the preparation of stock solutions, batch reactions, and chromatography analyses.

### 2.2. Materials preparation

In the present study, waste SD was employed as a lignocellulosic biomass feedstock for the preparation of both pristine and metal-loaded biochar materials. Initially, the collected SD was washed thoroughly three times with deionized water and then dried in an oven at 105 °C until a constant weight was attained. The dried material was subsequently pulverized and sieved through a 60-mesh screen to obtain a uniform particle size before being stored in airtight bags. The Ag/Ni-SDBC catalyst was synthesized using a one-step co-impregnation–pyrolysis method with slight modifications to the procedures reported by Xu *et al.*^27^ Briefly, 0.4247 g of silver nitrate (AgNO_3_) and 0.5942 g of nickel(ii) chloride hexahydrate (NiCl_2_·6H_2_O) were dissolved in 100 mL of deionized water in a 250 mL amber glass beaker to prepare an impregnation solution containing 25 mM of each metal precursor. The amber beaker was used to minimize the photoreduction of Ag ions during solution preparation. Subsequently, 5 g of dried SD powder was added to the solution and magnetically stirred for 2 h to promote the uniform distribution of Ag and Ni ions throughout the lignocellulosic matrix. The resulting suspension was then aged at room temperature (*T* ∼25 °C) for 12 h to facilitate the impregnation of the metal precursors into the sawdust structure. The impregnated biomass was recovered by filtration through a 0.22 µm membrane, dried at 105 °C overnight, and transferred to a 100 mL lidded ceramic crucible. Pyrolysis was carried out in a muffle furnace (AMF 25 N, Asahi-Rika, Chiba, Japan) under a continuous nitrogen flow (0.5 L min^−1^) at 700 °C for 120 min using a heating rate of 5 °C min^−1^. After naturally cooling to room temperature inside the furnace under a nitrogen atmosphere, the obtained Ag/Ni-SDBC was collected and washed several times with deionized water, followed by ethanol to eliminate residual impurities and unreacted precursors. Finally, the material was dried at 105 °C overnight, ground, sieved through a 100-mesh sieve, and stored in sealed bags away from light until further characterization and catalytic application. The metal loading, calculated based on the initial sawdust mass, was approximately 5.39 wt% Ag and 2.94 wt% Ni, corresponding to a total metal loading of 8.33 wt%. The Ag and Ni precursors were introduced at an equimolar ratio (Ag : Ni = 1 : 1). Moreover, pristine SDBC, Ag-SDBC, and Ni-SDBC were prepared following the same synthesis protocol as that used for Ag/Ni-SDBC, with appropriate modifications. For the preparation of pristine SDBC, the pretreated SD powder was directly transferred to a lidded ceramic crucible and pyrolyzed under the same operating conditions without the impregnation step or the addition of any metal precursor. In contrast, Ag-SDBC and Ni-SDBC were synthesized by introducing only AgNO_3_ or NiCl_2_·6H_2_O, respectively, with all other synthesis conditions remaining unchanged.

### 2.3. Characterization methods

Herein, the prepared catalysts were characterized using several techniques, including X-ray diffraction (XRD), Fourier transform infrared (FT-IR) spectroscopy, scanning electron microscopy (SEM), transmission electron microscopy coupled with energy-dispersive X-ray spectroscopy (TEM-EDX), the Brunauer–Emmett–Teller (BET) and Barrett–Joyner–Halenda (BJH) methods, and point of zero charge (pH_pZC_) analysis. Detailed information on the instrumentation and characterization categories is described in Text S2.

### 2.4. Experimental procedures

The degradation of LEV in the developed system was conducted in batch mode using 100 mL of the reaction solution in a 250 mL glass beaker placed on a magnetic stirrer. All experiments were carried out with an initial LEV concentration of 10 mg L^−1^, an oxidant concentration of 2 mM, a catalyst dosage of 0.5 g L^−1^ at neutral pH (pH = 7) and 25 °C under continuous magnetic stirring at 250 rpm. The solution's pH was adjusted, when required, using 0.1 mol L^−1^ NaOH or HCl solutions before the reaction was initiated. The oxidation reaction was initiated by simultaneously adding the catalyst and the oxidant, and the reaction was allowed to proceed for 100 min. Samples were collected at 10 min intervals throughout the reaction period. At each sampling interval, a 2 mL aliquot was withdrawn using a 3 mL syringe, transferred to a 5 mL centrifuge tube, and filtered through a 0.22 µm membrane to remove suspended catalyst particles. To terminate the reaction, 1 mL of the filtrate was promptly transferred into an amber sample vial preloaded with 1 mL of 100 mM sodium thiosulfate (Na_2_S_2_O_3_), ensuring rapid quenching of the residual oxidant. The residual LEV and oxidant concentrations were subsequently analyzed, while the degree of mineralization was evaluated by measuring the total organic carbon (TOC).

To distinguish the contribution of adsorption from that of catalytic oxidation, the adsorption performance of pristine SDBC, Ag-SDBC, Ni-SDBC, and Ag/Ni-SDBC was first evaluated in the absence of PI to determine the intrinsic adsorption capacity of each synthesized material toward LEV. Subsequently, the catalytic performances of the corresponding PI activation systems (SDBC/PI, Ag-SDBC/PI, Ni-SDBC/PI, and Ag/Ni-SDBC/PI) were explored to investigate the effectiveness of each catalyst in activating PI and promoting the generation of reactive oxidative species for LEV degradation. The oxidation performance of PI alone was also examined to assess its direct oxidation capability in the absence of a catalyst. Furthermore, the catalytic performance of Ag/Ni-SDBC was evaluated using different oxidants (H_2_O_2_, PMS, PS, and PI) under the same operating conditions to identify the most efficient oxidation system. In addition, the influence of key operating parameters on LEV degradation in the Ag/Ni-SDBC/PI system was systematically investigated using the one-factor-at-a-time (OFAT) approach. The effects of the initial solution pH (3–9), PI concentration (0.5–5 mM), Ag/Ni-SDBC dosage (0.1–1.0 g L^−1^), and initial LEV concentration (5–20 mg L^−1^) were individually evaluated, while maintaining all other experimental variables constant. After identifying the optimal value of each operating parameter, subsequent experiments were carried out under the optimal condition. Further, the influence of common water matrix constituents on the degradation performance was evaluated by examining the effects of NOM and selected inorganic anions. Each component was individually added to the pollutant solution prior to initiating the oxidation reaction. Humic acid (HA) was employed as a representative of NOM, while the investigated inorganic anions included chloride (Cl^−^), nitrate (NO_3_^−^), sulfate (SO_4_^2−^), and bicarbonate (HCO_3_^−^). To assess the fate of iodine species during the PI-based oxidation process, the concentrations of IO_4_^−^ ions, iodate ions (IO_3_^−^), hypoiodous acid (HOI), iodide ions (I^−^), molecular iodine (I_2_), and triiodide ions (I_3_^−^) were monitored throughout the reaction. Monitoring these species is essential to evaluate the environmental safety of the developed system. Furthermore, the reusability of the catalyst was evaluated by conducting five consecutive reaction cycles. After each reaction cycle, the catalyst was separated from the reaction mixture by vacuum filtration and sequentially washed with deionized water and ethanol to eliminate residual oxidant and adsorbed pollutant molecules from its surface. The recovered material was subsequently dried at 70 °C to constant weight and reused in the next cycle. To investigate the degradation mechanism of the developed system, scavenging experiments were conducted using several quenching agents. Phenol, *tert*-butyl alcohol (TBA), *p*-benzoquinone (*p*-BQ), furfuryl alcohol (FFA), and l-histidine (l-His) were separately added to the reaction solution to selectively quench specific reactive oxidative species. The influence of each scavenger on antibiotic degradation was evaluated to identify the predominant reactive species responsible for the oxidation process, elucidate their generation pathways, and quantify their respective contributions to the overall degradation mechanism. Moreover, the versatility of the developed system was evaluated by investigating the degradation of structurally diverse antibiotics, including chloramphenicol (CAP), sulfamethoxazole (SMX), tetracycline (TC), and ciprofloxacin (CIP). To examine the practical applicability of the proposed oxidation process, degradation experiments were conducted using multiple water matrices (deionized water, tap water, river water, lake water, seawater, domestic wastewater, and untreated pharmaceutical industrial wastewater). The collecting sites of these matrices are provided in Table S1. The collected water samples were left undisturbed for 24 h to facilitate the settling of suspended solids. The resulting supernatants were carefully decanted and subsequently spiked with LEV to an initial concentration of 10 mg L^−1^ before the degradation experiments. All experiments were performed in triplicate, and the results were reported as the mean values of three independent measurements.

The pollutant degradation efficiency was calculated according to eqn (1) in Table S2, the oxidant utilization rate was determined (Table S2 and eqn (2)) to assess the efficiency of oxidant consumption during the reaction, and the TOC removal efficiency was estimated using eqn (3) in Table S2. The adsorption capacity of the synthesized catalysts was calculated (Table S2 and eqn (4)) to assess their affinity for LEV adsorption. To elucidate the degradation kinetics of the developed system, zero-order (Table S2 and eqn (5)), pseudo-first-order (Table S2 and eqn (6)), and pseudo-second-order (Table S2 and eqn (7)) kinetic models were applied to the experimental data. The performance of each model was assessed using the coefficient of determination (*R*^2^) derived from the corresponding kinetic plots. A higher *R*^2^ value indicates better agreement between the experimental observations and model predictions, reflecting a more accurate description of the antibiotic degradation kinetics. The interaction between the catalysts and PI was quantitatively evaluated by calculating the synergy index (SI) from the pseudo-first-order rate constants of the adsorption, PI-alone, and catalyst/PI systems according to eqn (8) in Table S2. The SI was determined to assess the extent to which catalytic PI activation enhanced LEV degradation beyond the individual contributions of adsorption and direct PI oxidation, thereby providing a quantitative measure of the interaction between the catalyst and the oxidant.

### 2.5. Analytical methods

The concentrations of the target antibiotics (LEV, CAP, SMX, TC, and CIP) and iodine species (IO_4_^−^, IO_3_^−^, and I^−^) were determined using validated chromatographic methods. HOI was identified indirectly through phenol iodination, while the formation of I_2_/I_3_^−^ was qualitatively evaluated using the starch colorimetric method. Residual oxidants (PS, PMS, and H_2_O_2_) were quantified using established iodometric and colorimetric methods. The concentrations of leached silver and nickel were quantified using an inductively coupled plasma mass spectrometer (ICP-MS; 7700x, Agilent Technologies, Santa Clara, CA, USA). Water quality parameters, including TOC, chemical oxygen demand (COD), total suspended solids (TSS), total dissolved solids (TDS), pH, and turbidity, were analyzed according to Standard Methods. Detailed analytical procedures, operating conditions, and calibration protocols are provided in Text S3.

## 3. Results and discussion

### 3.1. Characterization results

Fig. 1 presents the XRD patterns of pristine SDBC and the metal-modified biochars. The broad peak centered at nearly 20° and 44.6° are ascribed to the (002) and (100) diffraction planes of the disordered graphitic carbon, respectively, while other sharp peaks are assigned to the inorganic mineral phases, such as silicon dioxide (SiO_2_), potassium chloride (KCl), and calcium carbonate (CaCO_3_), respectively.^28,29^ In the case of Ni-SDBC, the broad graphitic carbon peak shifts slightly to approximately 22°, indicating a change in the carbon structure following Ni incorporation. In addition, a diffraction peak observed at 44.7° is assigned to the (111) crystal plane of metallic Ni.^30^ The absence of other characteristic Ni reflections is likely attributable to the low Ni loading and/or the overlap with the diffraction peaks of the carbon matrix and inorganic mineral phases. The remaining sharp peaks are similarly associated with the inherent mineral constituents of the biochar. In the Ag-SDBC pattern, the broad graphitic carbon peak shifts to approximately 21.5°. Distinct diffraction peaks located at 38.2°, 44.3°, 64.5°, and 77.4° are indexed to the (111), (200), (220), and (311) planes of face-centered cubic metallic Ag, respectively, whereas the remaining peaks originate from the inorganic minerals present in the biochar.^31^ For Ag/Ni-SDBC, the broad peak of amorphous carbon can be clearly identified with sharp reflections of metallic Ag. The diffraction peak at 44.3° is likely associated with the overlapping reflections of Ag and Ni.

**Fig. 1:**
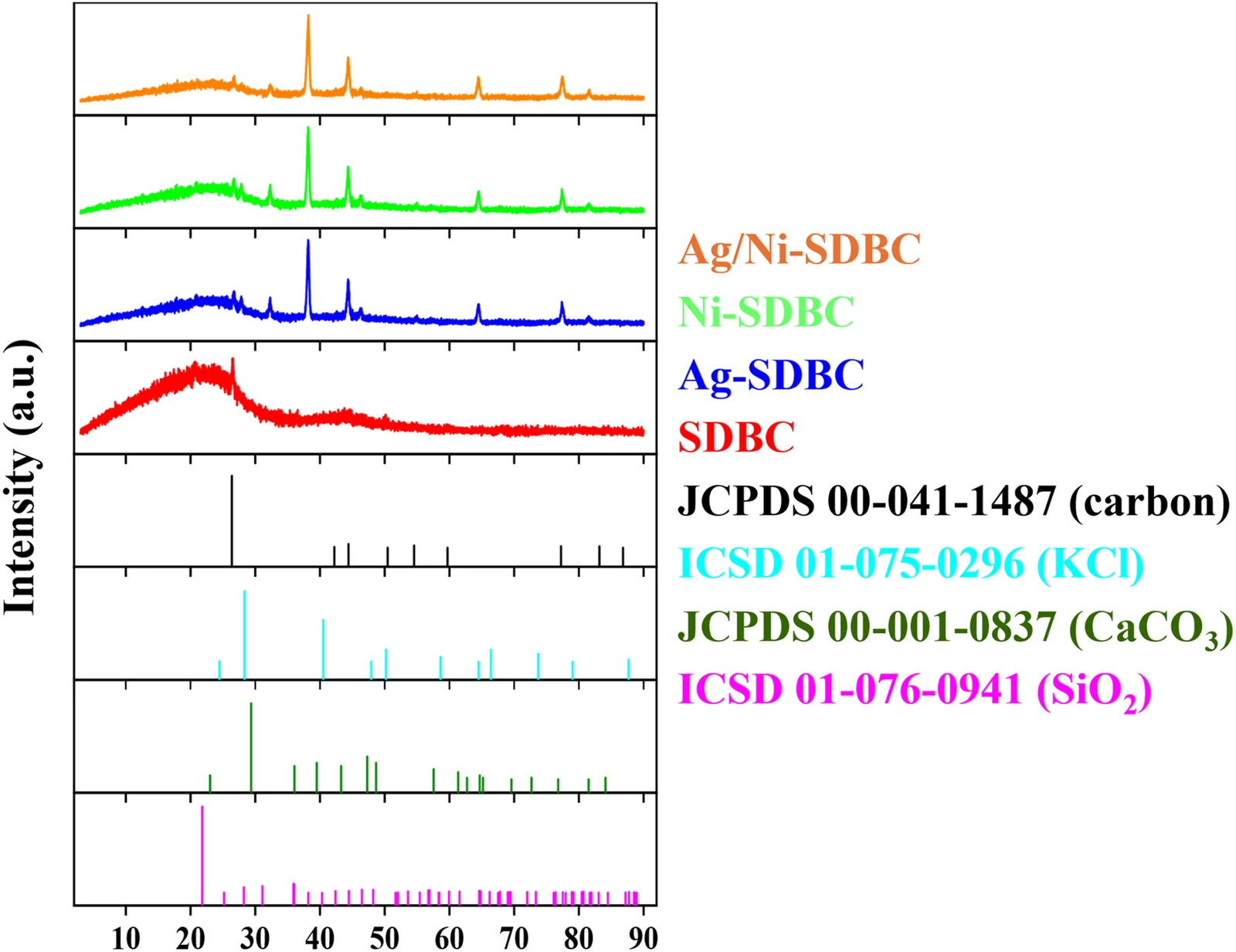
Comparison of the XRD patterns of pristine SDBC, Ag-SDBC, Ni-SDBC, and Ag/Ni-SDBC with standard reference patterns.

The FTIR spectra of intact SDBC and the fabricated metal-modified biochars (Ni-SDBC, Ag-SDBC, and Ag/Ni-SDBC) are presented in Fig. 2. FTIR spectrum of SDBC exhibits a broad absorption band centered at approximately 3030 cm^−1^, corresponding to the stretching vibration of hydroxyl groups (–OH). The absorption bands located at approximately 1700 cm^−1^ and (875 and 1575 cm^−1^) are assigned to the stretching vibrations of C=O and C–O functional groups, respectively.^32^ In addition, the peaks observed at 795 and 750 cm^−1^ are attributed to the out-of-plane bending vibrations of C–H groups related to the biochar's aromatic structure (Katiyar and Katiyar, 2025). Following metal loading, the FTIR spectra of both the monometallic- and bimetallic-modified biochars exhibit slight shifts in the characteristic absorption bands, indicating interactions between the incorporated metal species and the surface functional groups of the biochar.

**Fig. 2:**
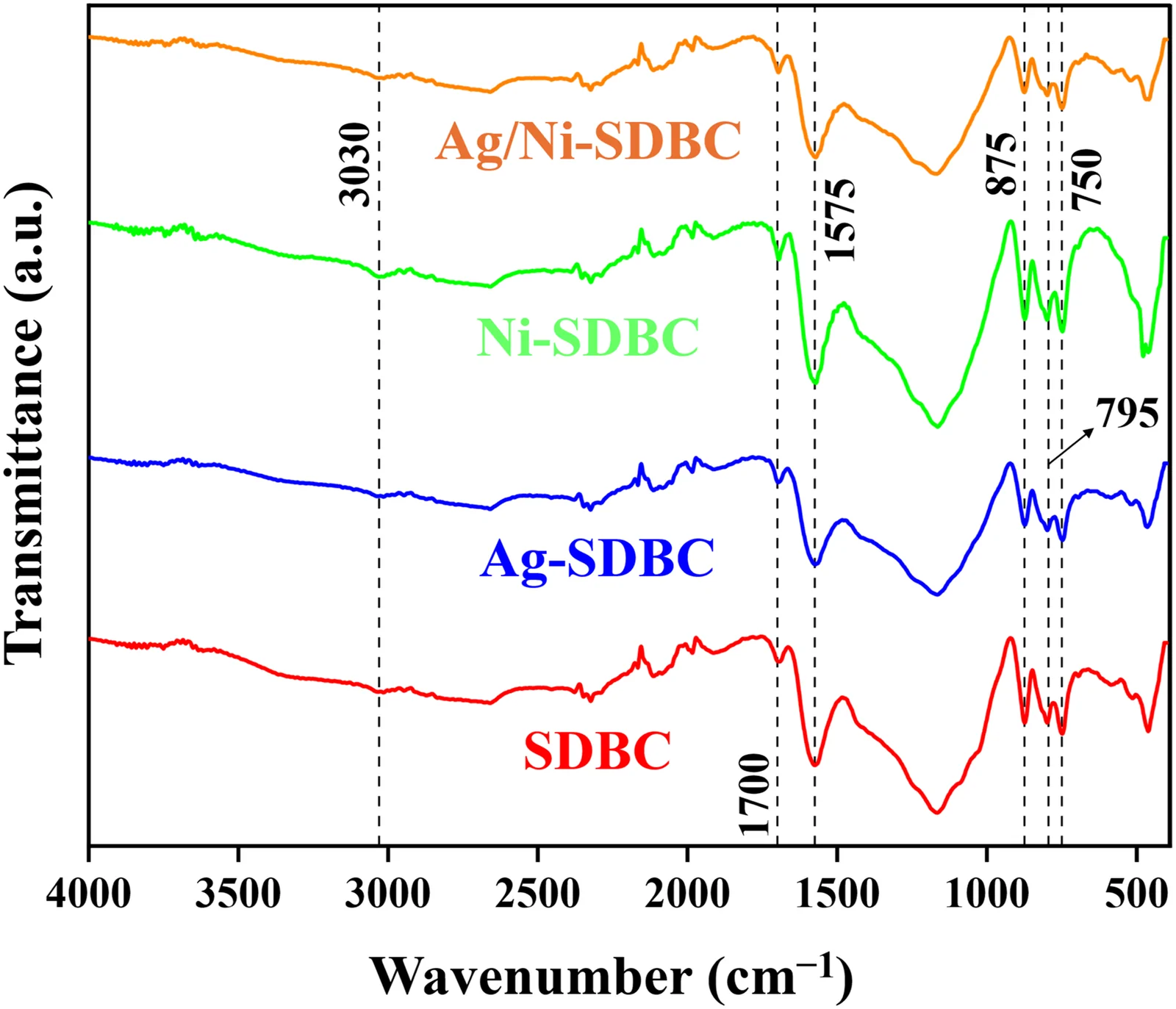
FT-IR spectra of the synthesized SDBC-based materials.

SEM micrographs of pristine SDBC, Ag-SDBC, Ni-SDBC, and Ag/Ni-SDBC at different magnifications are presented in Fig. 3. The unmodified biochar demonstrates a rough and heterogeneous structure, comprising irregular and plate-like morphologies with sharp edges and interparticle pores. The morphology of SDBC was largely retained after the incorporation of Ag or Ni, indicating that metal loading did not significantly alter the carbon framework. However, numerous bright, finely dispersed particles appeared on the biochar surface, confirming the successful deposition of Ag and Ni species. Similarly, Ag/Ni-SDBC largely retained the morphology of pristine SDBC, while exhibiting a higher density of finely dispersed particles across the biochar surface, confirming the successful co-deposition of Ag and Ni species. The higher metal loading in the bimetallic-loaded biochar provides more available active sites and promotes stronger interactions between the metal species and the carbon matrix, thereby ameliorating its activation potential towards PI. The preservation of the biochar morphology after metal loading confirms the successful immobilization of the metal species on the biochar surface without compromising its morphology. Furthermore, the TEM images of SDBC, Ag-SDBC, Ni-SDBC, and Ag/Ni-SDBC at different magnifications (Fig. 4(a–d)) reveal the heterogeneous morphology of the pristine biochar. In the metal-loaded samples, Ag and Ni species are distributed on the SDBC's surface, confirming the successful loading of both metals.

**Fig. 3:**
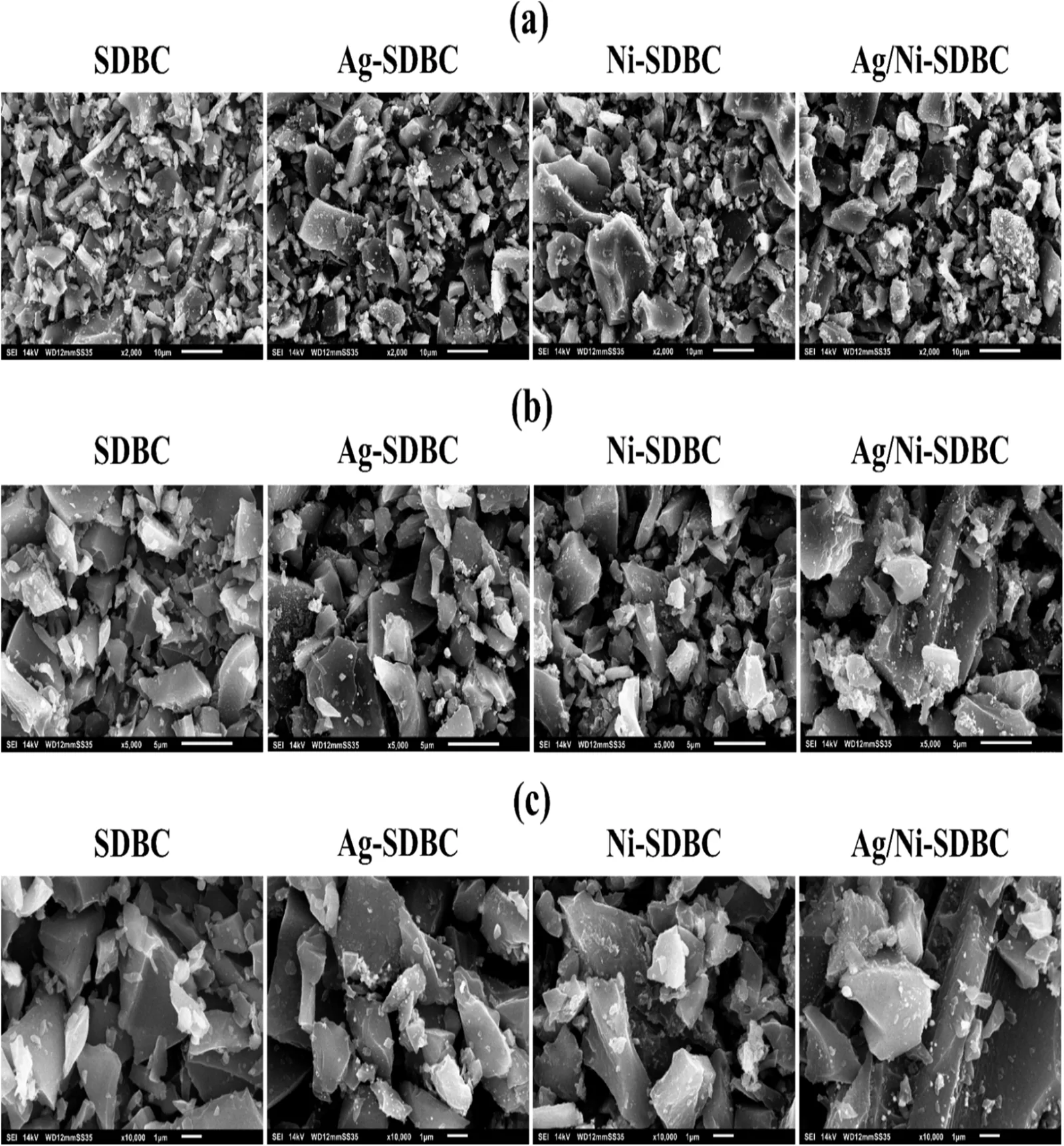
SEM images of pristine SDBC and the modified SDBC-based materials at different magnifications: (a) 2000×, (b) 5000×, and (c) 100 00×.

**Fig. 4:**
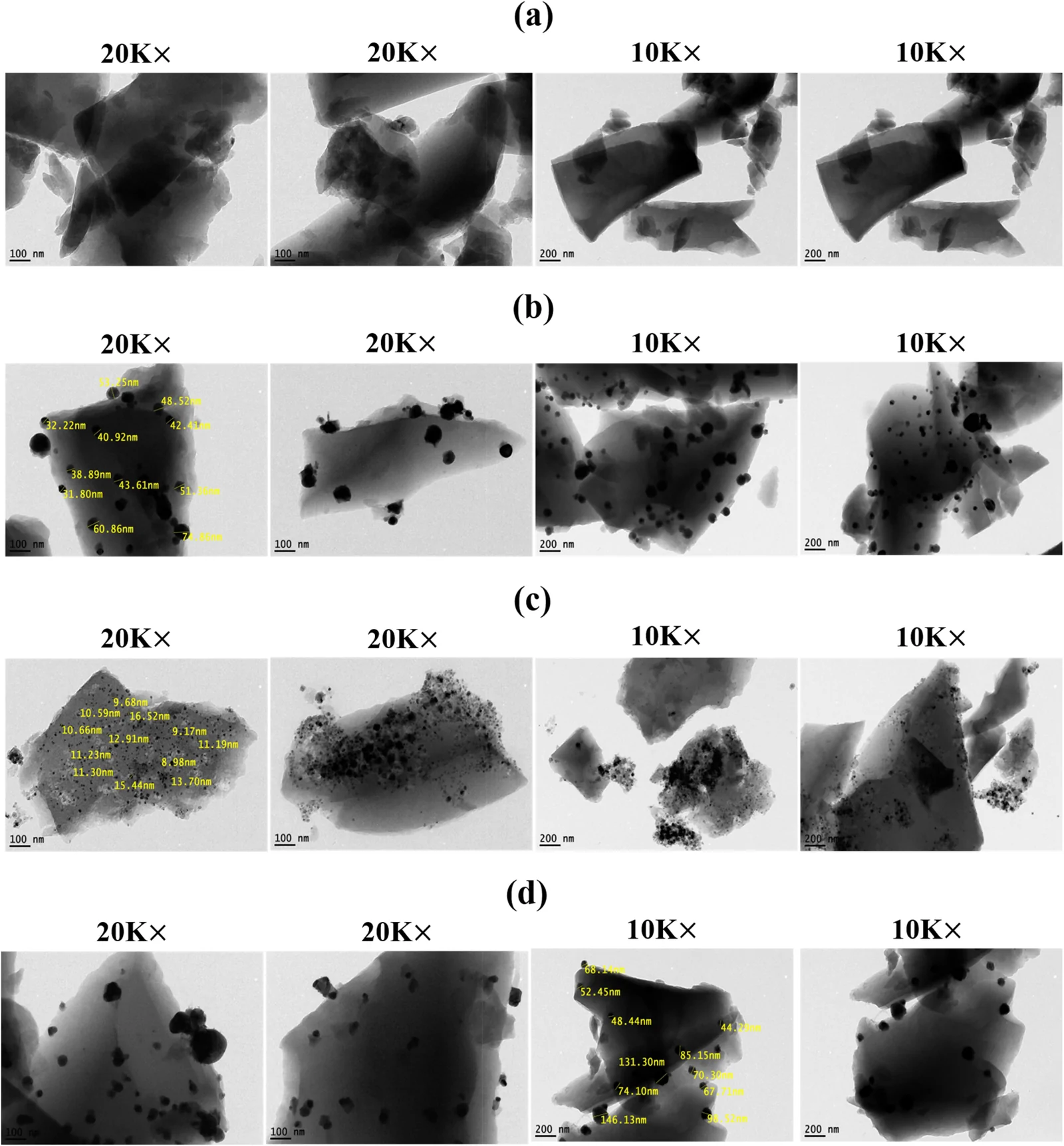
TEM images of (a) pristine SDBC, (b) Ag-SDBC, (c) Ni-SDBC, and (d) Ag/Ni-SDBC.

The chemical composition of the prepared materials is investigated using EDX, as shown in Fig. 5. The main components of the pristine SDBC are carbon (96.43%) and oxygen (2.41%), with trace ratios of inorganic minerals, whereas Ag-SDBC mainly contains carbon (82.18%) and oxygen (3.27%), with a significant Ag weight ratio (12.5%). Regarding Ni-SDBC, Ni with a weight ratio of 3.31% is noticed, as well as weight ratios of 88.43% and 2.68% for carbon and oxygen, respectively. In the case of Ag/Ni-SDBC, both Ni and Ag with weight ratios of 0.12% and 1.88% can be detected, with carbon (94.49%) and oxygen (2.7%) as the main components. The presence of Ag and Ni in the EDX pattern of the Ag/Ni-SDBC affirms the excellent loading of metals on the SDBC's surface. Fig. (S1–S4) demonstrate the EDX elemental mapping of the prepared catalysts, which reaffirms the excellent dispersion of metals on the SDBC's surface. The difference between weight ratios of Ag and Ni obtained by EDX analysis and nominal loading could be due the incomplete loading of metals on the biochar's surface. Further, metal losses could take place during the preparation through several washing.

**Fig. 5:**
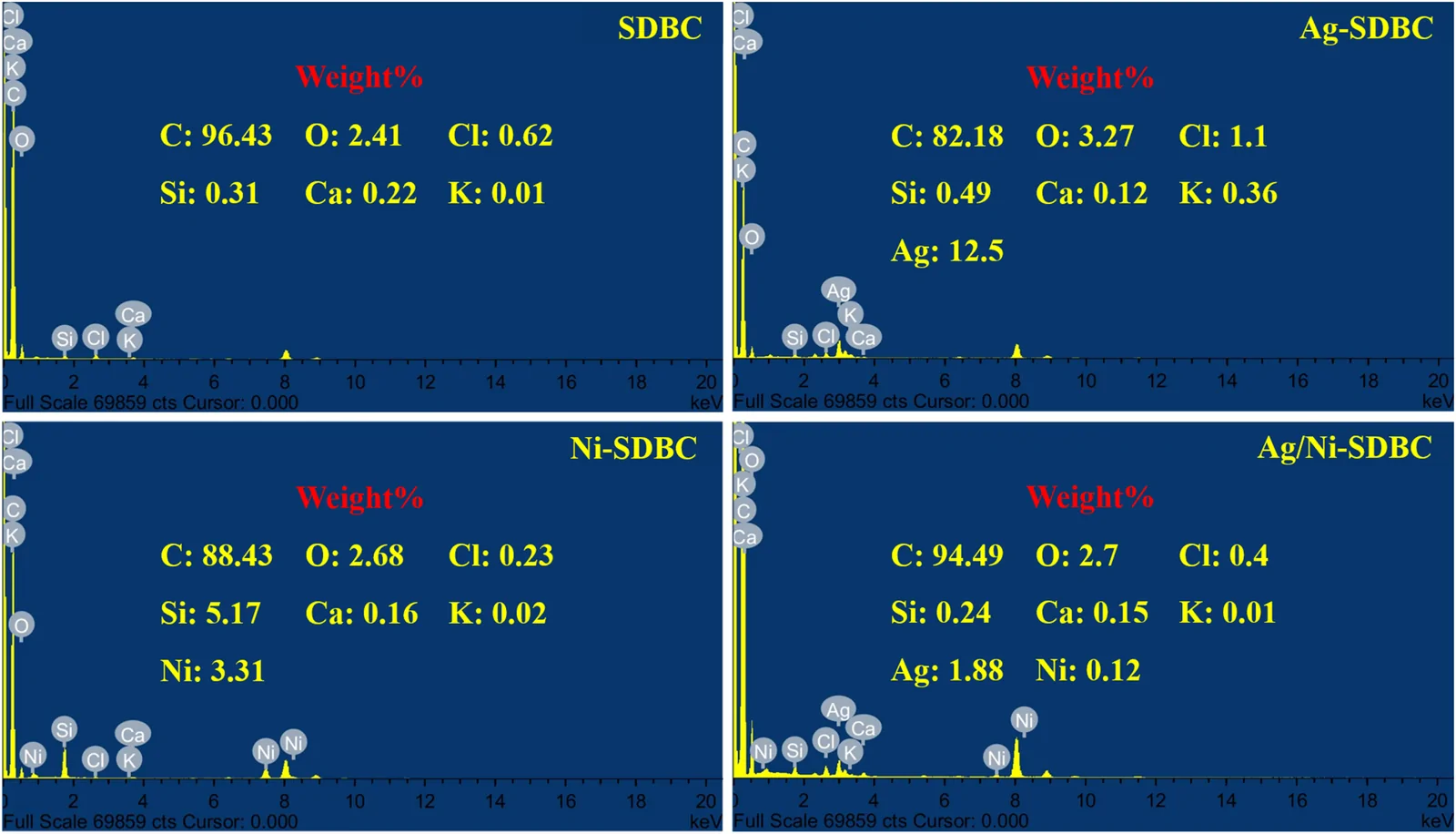
EDX spectra of unloaded and metal-loaded biochar.

The textural features of the pristine SDBC and metal-loaded SDBC are listed in Table 1, whereas the adsorption–desorption isotherms and pore size distributions are portrayed in Fig. 6 and 7, respectively. The prepared materials exhibited a sharp rise in the amount of adsorbed nitrogen at very low relative pressure followed by a limited rise in nitrogen uptake with increasing the relative pressure, exhibiting the abundance of micropores. Further, the adsorption and desorption lines nearly overlap with a negligible hysteresis loop, reconfirming the dominance of micropores with limited participation of mesopores. The unloaded SDBC owned a surface area (SA) of 401.57 m^2^ g^−1^, a total pore volume (*V*_p_) of 0.199 cm^3^ g^−1^, a micropore volume of 0.1217 cm^3^ g^−1^, and a micropore area of 344.71 m^2^ g^−1^, indicating that micropores constitute most of the surface area. In the case of Ag-SDBC and Ni-SDBC, the SA increased to 448.56 and 467.52 m^2^ g^−1^, respectively, while the micropore area decreased to 181.77 and 203.51 m^2^ g^−1^, accompanied by a reduction in micropore volume. Moreover, the *V*_p_ of Ag-SDBC and Ni-SDBC increases to 0.2518 and 0.2269 cm^3^ g^−1^, respectively. The reduction in micropore area and volume can be attributed to the partial occupation or blockage of micropores by the incorporated metal species. However, the loaded metals generate additional interparticle pores or widen the existing pores during metal impregnation and pyrolysis. A similar increase in SA following metal loading has been reported by Zhang *et al.*, who attributed the enhanced SA of water hyacinth-derived biochar after MnO_2_ incorporation to the formation of additional pores.^33^ In contrast, the SA of Ag/Ni-SDBC is 417.8 m^2^ g^−1^, which is lower than that of the monometallic-loaded biochars and higher than that of the pristine SDBC. Besides, the *V*_p_ decreased compared to Ag-SDBC and Ni-SDBC, while the micropore area and volume are the lowest among all materials. The decline in SA, pore volume, and micropore area can be attributed to the greater occupation and blockage of micropores by the co-incorporated Ni and Ag species than in the monometallic-loaded biochars.

**Table 1:** Physicochemical textural characteristics of the prepared SDBC-based materials

Parameter	SDBC	Ag-SDBC	Ni-SDBC	Ag/Ni-SDBC
SA (m^2^ g^−1^)	401.57	448.56	467.52	417.8
*V*_P_ (cm^3^ g^−1^)	0.199	0.2518	0.2269	0.2048
Micropore area (m^2^ g^−1^)	344.71	181.77	203.51	190.8
Micropore volume (cm^3^ g^−1^)	0.1217	0.1125	0.0842	0.0759
Average pore diameter (*D*_P_, nm)	1.4121	2.4757	1.6544	1.591

**Fig. 6:**
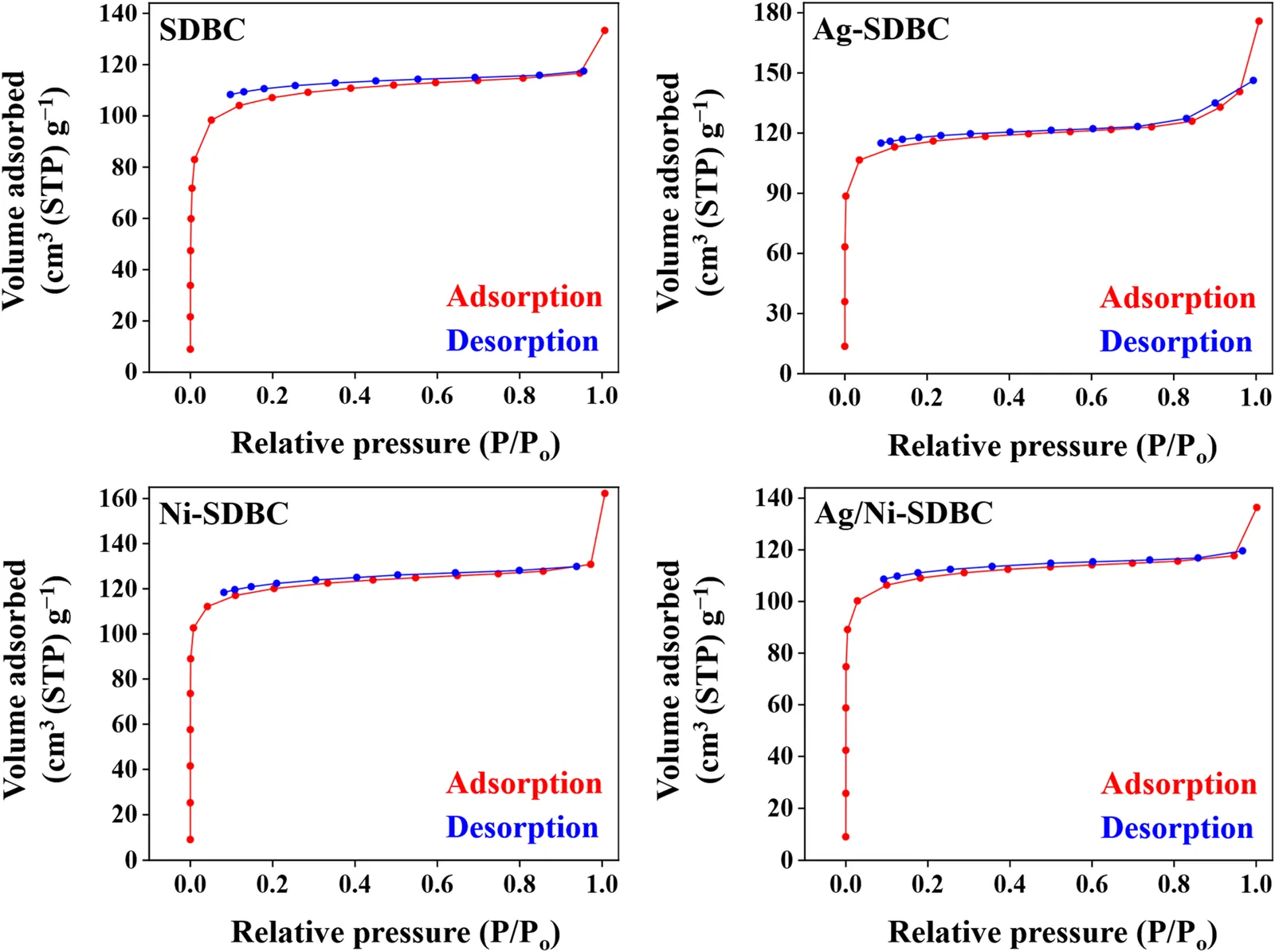
Adsorption–desorption isotherms of the synthesized materials.

**Fig. 7:**
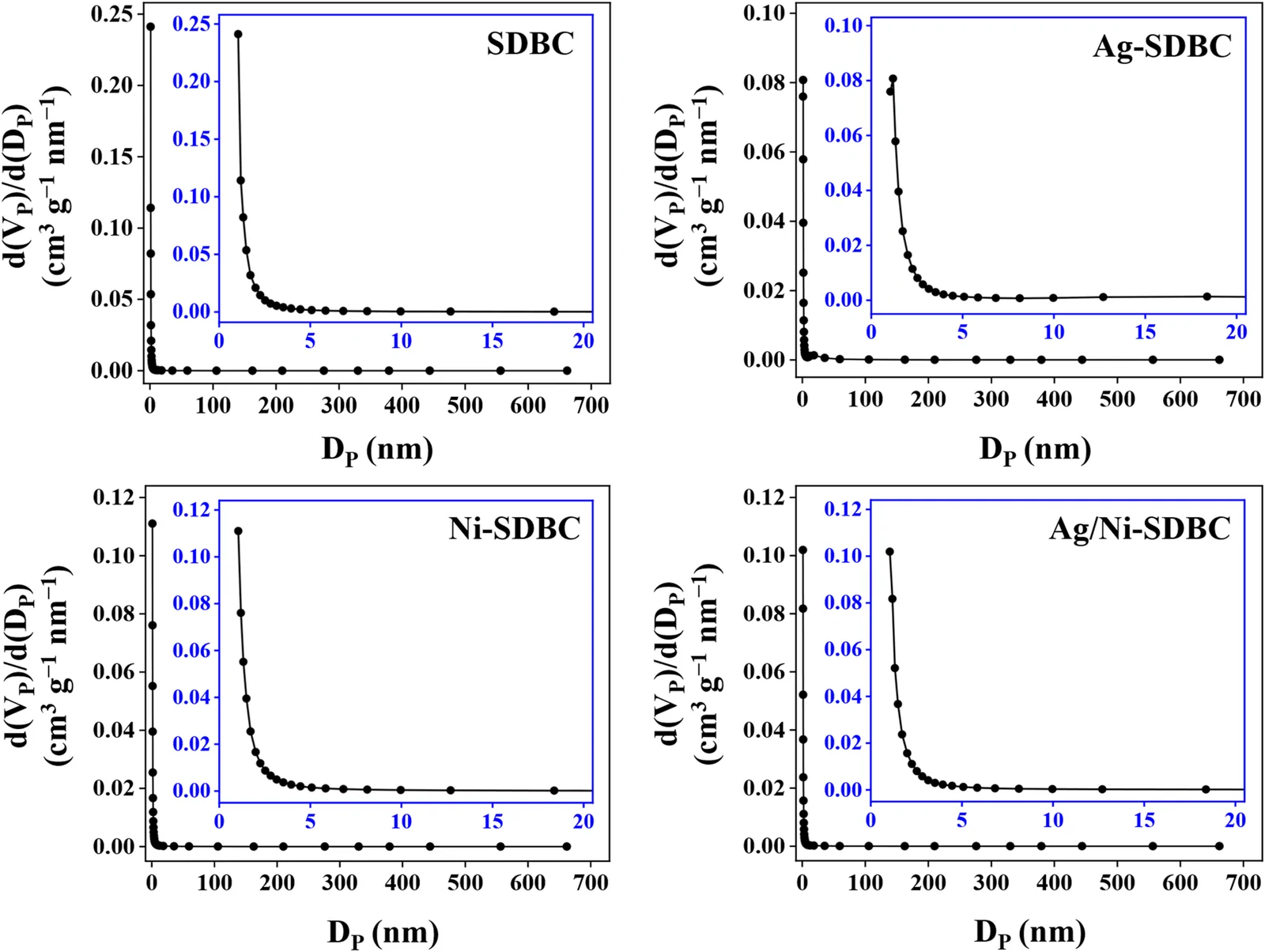
Pore size distributions of pristine SDBC, Ag-SDBC, Ni-SDBC, and AG/Ni-SDBC (insets: enlarged views of the 1–20 nm pore size range).

### 3.2. Evaluation of the catalytic performance of the Ag/Ni-SDBC/PI system

#### 3.2.1. Adsorption experiments

The adsorption experiments of the synthesized catalysts toward LEV were conducted in the absence of PI at an initial LEV concentration of 10 mg L^−1^, a catalyst dosage of 0.5 g L^−1^, neutral pH, room temperature, and a contact time of 100 min. As shown in Fig. 8(a) and Table S3, Ag/Ni-SDBC exhibited the highest adsorption capability among the tested materials, achieving a LEV removal efficiency of 28.35%, accompanied by a pseudo-first-order rate constant (*k*_1_) of 0.004 min^−1^ and an adsorption capacity of 14.18 mg g^−1^. The adsorption performance decreased for Ni-SDBC, which removed 22.8% of LEV with a *k*_1_ value of 0.0032 min^−1^ and an adsorption capacity of 11.4 mg g^−1^. Further, Ag-SDBC achieved 19.13% LEV removal, corresponding to a *k*_1_ value of 0.0026 min^−1^ and a capacity of 9.57 mg g^−1^. In contrast, pristine SDBC showed the weakest adsorption behavior, yielding only 15.23% LEV removal, with a *k*_1_ value of 0.0019 min^−1^ and an adsorption capacity of 7.62 mg g^−1^. The results revealed that the adsorption capacities of the prepared materials followed the order Ag/Ni-SDBC > Ni-SDBC > Ag-SDBC > SDBC. The higher specific surface areas (SA) of Ni-SDBC and Ag-SDBC (Table 1) provided a greater number of accessible adsorption sites and promoted greater contact between LEV molecules and the adsorbent surface, resulting in improved LEV adsorption capacities relative to pristine SDBC.^34^ Interestingly, Ag/Ni-SDBC achieved the highest adsorption capacity despite possessing a lower SA than both Ni-SDBC and Ag-SDBC (Table 1). This enhancement is likely associated with the loading of Ag and Ni, which modified the physicochemical properties of the biochar surface. Such modifications may have facilitated interactions between metal species and surface functional groups, leading to the formation of more reactive binding sites.^35,36^

**Fig. 8:**
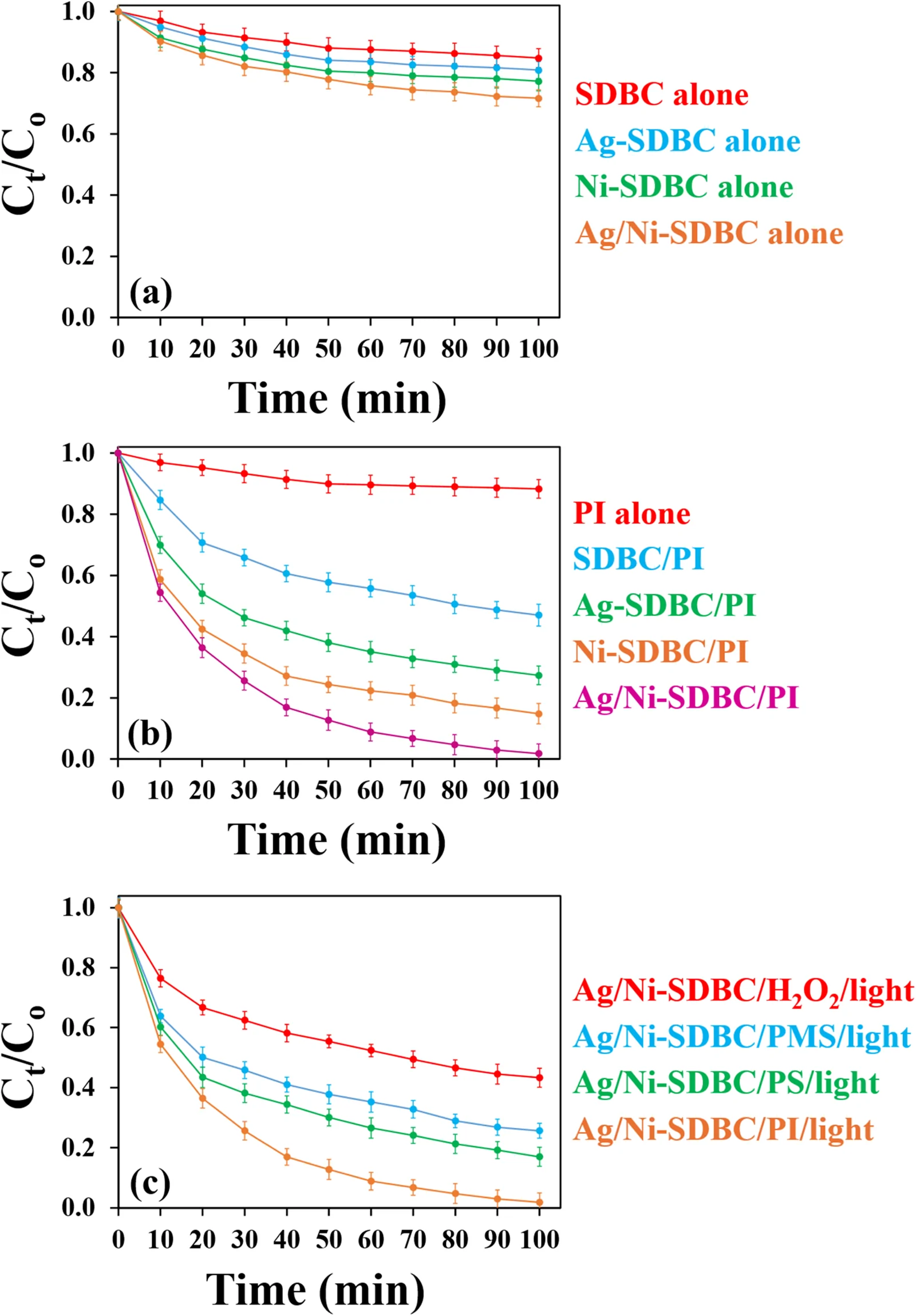
(a)–(c) LEV removal efficiency in different reaction systems. Experimental conditions: [LEV]_o_ = 10 mg L^−1^, [oxidant]_o_ = 2 mM, [catalyst]_o_ = 0.5 g L^−1^, pH = 7, *T* = 25 °C, and reaction time = 100 min.

The adsorption behavior of LEV can be further interpreted by considering the relationship between its acid–base speciation and the surface charge characteristics of the synthesized materials. LEV is an amphoteric compound with p*K*_a_ values of approximately 5.83 and 8.75. Consequently, under the neutral pH conditions employed in these experiments, LEV predominantly existed in its zwitterionic form, containing both negatively charged carboxylate and positively charged piperazinyl groups.^37^ The pH_pZC_ values of SDBC, Ag-SDBC, Ni-SDBC, and Ag/Ni-SDBC were determined to be 6.5, 6.4, 6.55, and 6.17, respectively, as shown in Fig. S5. Since the experimental pH exceeded the pH_pZC_ of all synthesized materials, their surfaces were expected to carry a net negative charge.^38^ Under these conditions, strong electrostatic attraction was unlikely because zwitterionic LEV possesses both positive and negative charge centers. Consequently, the SDBC-based materials exhibited only moderate LEV adsorption efficiencies, indicating that electrostatic attraction was not the dominant adsorption mechanism. Nevertheless, the positively charged piperazinyl group of LEV could still interact electrostatically with negatively charged surface sites, thereby contributing to adsorption to some extent. In addition to these limited electrostatic interactions, several non-electrostatic mechanisms likely played significant roles in the adsorption process, such as π–π electron donor–acceptor interactions between the aromatic rings of LEV and the graphitic domains of the biochars, hydrogen bonding involving oxygen-containing surface functional groups, and surface complexation with Ag and Ni active sites.^35,39^

#### 3.2.2. Catalytic PI activation systems

The catalytic degradation experiments were performed at an initial LEV concentration of 10 mg L^−1^, a PI concentration of 2 mM, a catalyst dosage of 0.5 g L^−1^, neutral pH, and room temperature for 100 min. As illustrated in Fig. 8(b) and Table S3, the uncatalyzed PI (PI-alone) system exhibited limited oxidative activity toward LEV, achieving a removal efficiency of only 11.69% and a *k*_1_ value of 0.0015 min^−1^ under the investigated conditions. This limited performance is mainly due to the relatively low redox potential of PI (1.6 V *vs.* NHE). Accordingly, efficient PI activation is required to promote the formation of reactive radical species, thereby improving the oxidation process and enhancing pollutant degradation.^40^ Thus, combining PI with the synthesized SDBC-based catalysts resulted in a substantial improvement in degradation performance. LEV removal efficiencies increased to 52.95%, 72.63%, 85.2%, and 98.2% in the SDBC/PI, Ag-SDBC/PI, Ni-SDBC/PI, and Ag/Ni-SDBC/PI systems, respectively, while the corresponding *k*_1_ values rose to 0.0089, 0.0155, 0.0225, and 0.04 min^−1^. Moreover, the SI values increased progressively from 2.62 for the SDBC/PI system to 3.78 for Ag-SDBC/PI, 4.79 for Ni-SDBC/PI, and 7.27 for Ag/Ni-SDBC/PI, indicating progressively stronger interactions between PI and the modified biochar catalysts. Notably, the Ag/Ni-SDBC/PI system exhibited the highest SI value, confirming that loading of Ag and Ni attained the greatest enhancement in catalytic performance by promoting more efficient PI activation and reactive species generation during redox cycling of Ag and Ni than either single-metal-loaded or pristine biochar.

In the presence of PI, LEV elimination resulted from the combined contributions of adsorption and catalytic oxidation. In contrast, in the absence of PI, pollutant removal was solely attributable to adsorption onto the catalysts' surfaces. Therefore, LEV degradation in the catalyst-mediated PI activation systems exhibited substantially higher LEV degradation efficiencies than the corresponding adsorption systems. This improved performance highlights the pivotal role of catalytic PI activation in driving pollutant degradation and can be ascribed to the sustained production of ROS and RIS, which increase the overall oxidative potential of the system and facilitate the efficient degradation of LEV. FT-IR analysis (Fig. 2) revealed that the SDBC surface is enriched with oxygen-containing functional groups, which can act as electron transfer mediators, thereby promoting PI activation and the formation of both 
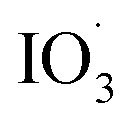
 and HO˙ radicals (eqn (1) and (2)).^11,41^ In addition, the presence of IO_4_^−^ ions can initiate a series of redox reactions that sustain the generation and accumulation of highly reactive species. These reactions can lead to the formation of 
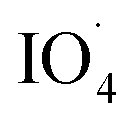
 radicals (eqn (3) and (4)),^41,42^ O_2_˙^−^ radicals (eqn (5) and (6)),^43,44^ and ^1^O_2_ (eqn (7) and (8)).^45,46^ Additional 
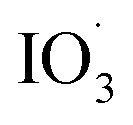
 radicals can be generated through the oxidation of IO_3_^−^ ions by HO˙ radicals (eqn (9)).^47^ On the other hand, O_2_˙^−^ radicals can react with HO˙ radicals to generate more ^1^O_2_ and (eqn (10)) and with H_2_O molecules to produce both ^1^O_2_ and H_2_O_2_ (eqn (11)).^41,45,48^ H_2_O_2_ can also be generated through a sequence of protonation and disproportionation reactions involving O_2_˙^−^ radicals, as outlined in eqn (12)–(15).^47–49^ The *in situ*-generated H_2_O_2_ can participate in secondary oxidation reactions by reacting with O_2_˙^−^ radicals to produce HO˙ radicals and ^1^O_2_ (eqn (16)). In addition, H_2_O_2_ can activate PI, leading to the formation of 
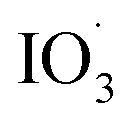
 and O_2_˙^−^ radicals (eqn (17)).^47^1
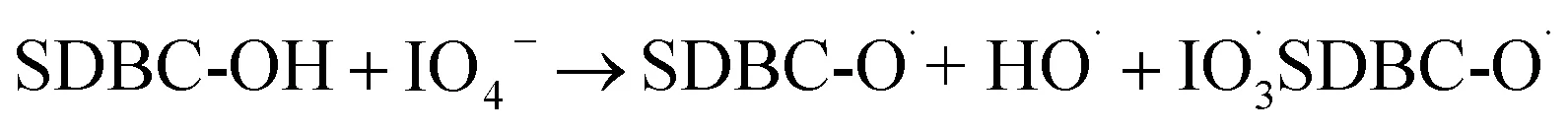
2
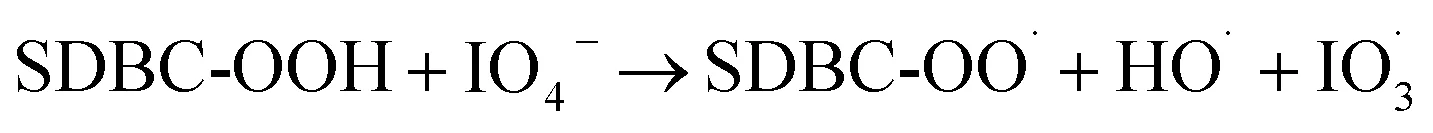
3
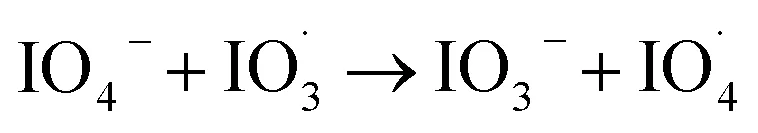
4
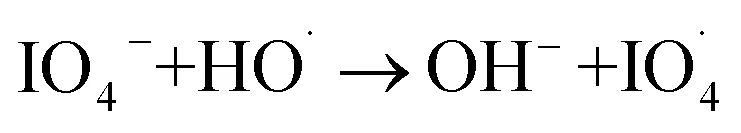

5 3IO^−^_4_ + 2OH^−^ → H_2_O + 3IO^−^_3_ + 2O_2_˙^−^

6 IO^−^_4_ + 2OH^−^ + O_2_ → H_2_O + IO^−^_3_ + 2O_2_˙^−^

7 IO^−^_4_ + 2O_2_˙^−^ + H_2_O → IO^−^_3_ + 2OH^−^ + ^1^O_2_ + ^1^O_2_

8 IO_4_^−^ + IO_4_^−^ → 2IO_3_^−^ + ^1^O_2_ + ^1^O_2_

9
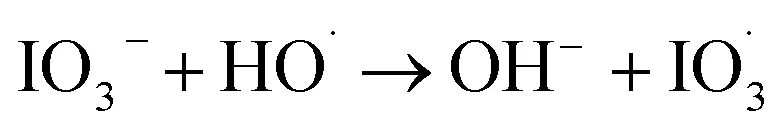

10 O_2_˙^−^ + HȮ → OH^−^ + ^1^O_2_

11 2O_2_˙^−^ + 2H_2_O → 2OH^−^ + H_2_O_2_ + ^1^O_2_

12
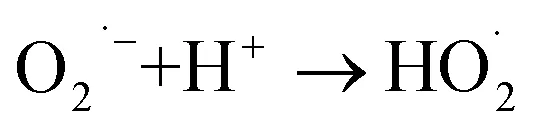
13
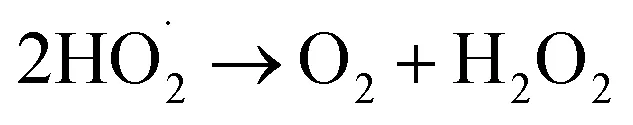
14
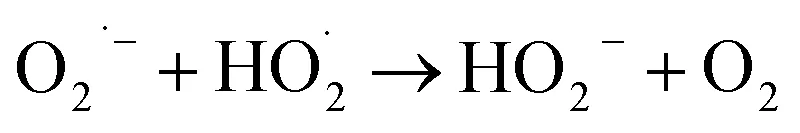

15 HO_2_^−^ + H^+^ → H_2_O_2_

16 O_2_˙^−^ + H_2_O_2_ → OH^−^ + HO˙ +^1^O_2_

17
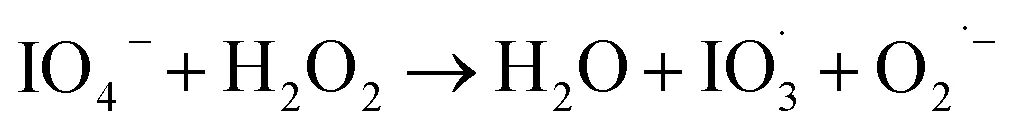


The enhanced degradation performance of the metal-loaded SDBC/PI systems relative to the pristine SDBC/PI system is primarily attributed to the incorporation of Ag and Ni, which introduced additional redox-active sites for PI activation. During the catalytic cycle in the metal-loaded SDBC/PI systems, the oxidation of reduced metal species can regenerate higher oxidation-state metal centers promoting electron transfer which initiates additional PI activation pathways,^50^ thereby promote the formation of HO˙ and 
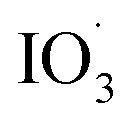
 radicals (eqn (18–21)).^46,48^ Moreover, the released electrons can be captured by dissolved oxygen (O_2_), resulting in the generation of O_2_˙^−^ radicals and H_2_O_2_ through the pathways described in eqn (22) and (23). Furthermore, the generated H_2_O_2_ can accept additional electrons to produce more HO˙ radicals (eqn (24)).^46,47,49,51^ Besides, ^1^O_2_ can undergo one-electron reduction to produce additional O_2_˙^−^ radicals (eqn (25)).^45^ As a result, the metal-loaded SDBC/PI systems can generate larger amounts of reactive species than pristine SDBC/PI system, resulting in significantly improved LEV degradation. Notably, the Ni-SDBC/PI system exhibited higher activity than the Ag-SDBC/PI system. The Ag/Ni-SDBC/PI system exhibited higher LEV degradation efficiency than that of monometallic-loaded systems. The simultaneous presence of both metals can enhance the availability of redox-active sites and facilitate continuous electron transfer, leading to more effective PI activation and increased generation of reactive species compared with the corresponding monometallic-loaded systems.

18 IO_4_^−^ + e^−^ → IO_3_^−^ + Ȯ^−^

19 O˙^−^ + H^+^ ↔ HO˙

20 O˙^−^ + H_2_O ↔ OH^−^ + HO˙

21
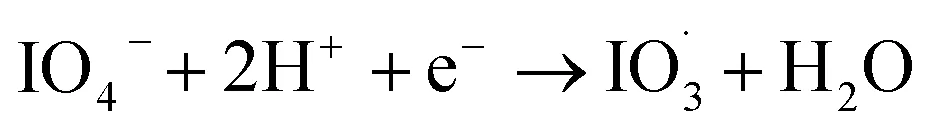

22 O_2_ + e^−^ → O_2_˙^−^

23 O_2_ + 2H^+^ + 2e^−^ → H_2_O_2_

24 H_2_O_2_ + e^−^ → OH^−^ + HO˙

25 ^1^O_2_ + e^−^ → O_2_˙^−^

#### 3.2.3. Evaluation of different oxidants for catalytic LEV degradation using Ag/Ni-SDBC

The effectiveness of different oxidants (H_2_O_2_, PMS, PS, and PI) for LEV degradation using the Ag/Ni-SDBC catalyst was assessed under identical experimental conditions (initial LEV concentration = 10 mg L^−1^, oxidant concentration = 2 mM, catalyst dosage = 0.5 g L^−1^, neutral pH, room temperature, and reaction time = 100 min). As illustrated in Fig. 8(c) and Table S4, PI exhibited the highest activity, achieving 98.2% LEV removal with a *k*_1_ value of 0.04 min^−1^. In comparison, PS attained 83.03% LEV degradation with a *k*_1_ value of 0.0203 min^−1^, whereas PMS and H_2_O_2_ yielded lower removal efficiencies of 74.38% and 56.75%, corresponding to *k*_1_ values of 0.0161 and 0.0099 min^−1^, respectively. The results revealed that the pollutant degradation efficiency and kinetic rates were markedly influenced by the oxidant type, following the order PI > PS > PMS > H_2_O_2_. Moreover, the effective activation of all investigated oxidants by Ag/Ni-SDBC reflects its broad catalytic applicability and demonstrates its potential for implementation in a wide range of advanced oxidation processes. Further insight was provided by the oxidant consumption data summarized in Table S4. The oxidant utilization rates followed the same trend observed for LEV degradation and reaction kinetics: PI (91.35%) > PS (82.62%) > PMS (76.93%) > H_2_O_2_ (63.84%). The close agreement between oxidant consumption, pollutant degradation efficiency, and kinetic rate constants outlines that enhanced oxidant utilization promoted more effective activation pathways and greater generation of reactive oxidative species. Consequently, the increased availability of these reactive species accelerated LEV degradation and improved the overall treatment performance of the system.

The superior performance of the PI-based system compared with the PS-, PMS-, and H_2_O_2_-based systems can be attributed to the relatively long I–O bond length in PI (approximately 1.78 Å), which facilitates bond cleavage and enables more efficient activation of PI.^52,53^ Consequently, PI can be more readily converted into a variety of highly reactive radical and non-radical species than other examined oxidants. The PS-based system exhibited the second-highest activity among the investigated oxidants. This performance can be attributed to its relatively long O–O bond length (approximately 1.497 Å), which renders the oxidant more susceptible to activation and promotes the generation of sulfate radicals (SO_4_˙^−^) and HO˙ radicals.^54^ However, the O–O bond in PS is still considerably shorter than the I–O bond in PI, making PS less susceptible to bond cleavage and activation. Consequently, PS generates lower reactive species than that of the PI-based system. In the case of PMS-based system, the degradation efficiency further decreased compared to PS owing to its shorter O–O bond length (approximately 1.46 Å).^55^ H_2_O_2_ showed the poorest performance among all oxidants, exhibiting the lowest degradation efficiency, reaction rate constant, and oxidant utilization. In contrast to PI, PS-, and PMS-based systems, H_2_O_2_ activation produces a comparatively limited spectrum of reactive species, restricting its oxidation potential.^56^ Its relatively short O–O bond length (approximately 1.453 Å) further hinders activation, leading to less efficient reactive species production.^55^ In addition, hydroxyl radicals, the primary oxidizing species in H_2_O_2_-based systems, possess shorter lifetimes and lower selectivity than sulfate radicals and reactive iodine species, resulting in comparatively lower degradation efficiency.^44,52^ Collectively, these findings indicated that the Ag/Ni-SDBC/PI system provided the most favorable conditions for oxidant activation and reactive species generation among the investigated processes. Owing to its superior degradation performance and efficient oxidant utilization, this system was employed in all subsequent LEV degradation investigations.

### 3.3. Effect of operating conditions on LEV degradation in the Ag/Ni-SDBC/PI system

Previous studies on PI-based advanced oxidation systems have shown that operational parameters, including the initial solution pH, PI concentration, catalyst dosage, and pollutant concentration, play critical roles in determining the degradation efficiency and reaction kinetics of organic contaminants.^51,57^ Accordingly, the influence of these parameters on LEV degradation in the Ag/Ni-SDBC/PI system was systematically evaluated by varying one factor at a time while keeping all other experimental conditions unchanged. The experiments were carried out at room temperature for a reaction period of 100 min.

To investigate the impact of pH, LEV degradation by the Ag/Ni-SDBC/PI system was examined over a pH range of 3–9, and the corresponding results are shown in Fig. 9(a) and Table S5. The most favorable degradation performance was obtained under acidic conditions. Complete LEV removal was achieved within 60 and 80 min at pH 3 and 5, respectively, with *k*_1_ values of 0.065 and 0.0509 min^−1^. When the initial pH was increased to 7, the system still exhibited excellent activity, achieving 98.2% LEV degradation after 100 min, although the reaction rate constant declined to 0.04 min^−1^. A further increase in pH to 9 resulted in a noticeable deterioration in catalytic performance, with the degradation efficiency decreased to 82.31% and the rate constant dropped to 0.0195 min^−1^. Similar pH-dependent trends were reported for other PI-based oxidation systems.^12,50,58^ Although the degradation rate decreased with increasing pH, the Ag/Ni-SDBC/PI process maintained high LEV removal throughout the investigated pH range, demonstrating its operational flexibility and potential applicability under broad pH range. The superior performance observed under acidic and neutral conditions can be attributed to the predominance of IO_4_^−^ ions at pH values below 8, which is more susceptible to activation and subsequent reactive species generation.^58^ Moreover, acidic conditions provide an elevated concentration of hydrogen ions (H^+^), which can react with unpaired electrons on the Ag/Ni-SDBC surface generated by abundant oxygen-containing functional groups. This process promotes the generation of hydrogen radicals (H˙), as illustrated in eqn (26).^59^ The H˙ radicals can subsequently react with IO_4_^−^ ions to produce highly reactive species, including 
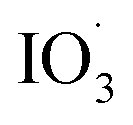
 and HO˙ radicals, thereby accelerating LEV degradation (eqn (27) and (28)).^12,58^ The reduced degradation efficiency observed at pH values above 8 is likely related to a shift in PI speciation. Under alkaline conditions, H_2_I_2_O_10_^4−^ becomes the predominant oxidant species, replacing IO_4_^−^ ions. Because H_2_I_2_O_10_^4−^ exhibits a considerably lower oxidation potential (0.7 V *vs.* NHE) than IO_4_^−^ ions (1.6 V *vs.* NHE), its ability to generate reactive species is diminished, resulting in lower oxidation efficiency and slower LEV degradation.^40^ Considering both its high degradation efficiency and practical applicability, pH 7 was selected for all subsequent experiments. Operation under neutral pH offers significant practical benefits by limiting the requirement for chemical pH regulation, which can lower treatment costs and improve the overall feasibility and sustainability of the process for wastewater treatment applications. To further elucidate the degradation behavior of LEV, the experimental data were also fitted using zero-order and pseudo-second-order kinetic models (Fig. S6). A comparison of the corresponding *R*^2^ values revealed that the pseudo-first-order model consistently produced the best fit across the entire pH range investigated. Accordingly, the pseudo-first-order kinetic model was selected to evaluate and compare the degradation performance in all subsequent experiments.

26 H^+^ + e_aq_^−^ → H˙

27
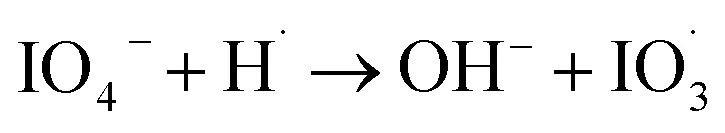

28 IO_4_^−^ + H˙ → IO_3_^−^ + HO˙

**Fig. 9:**
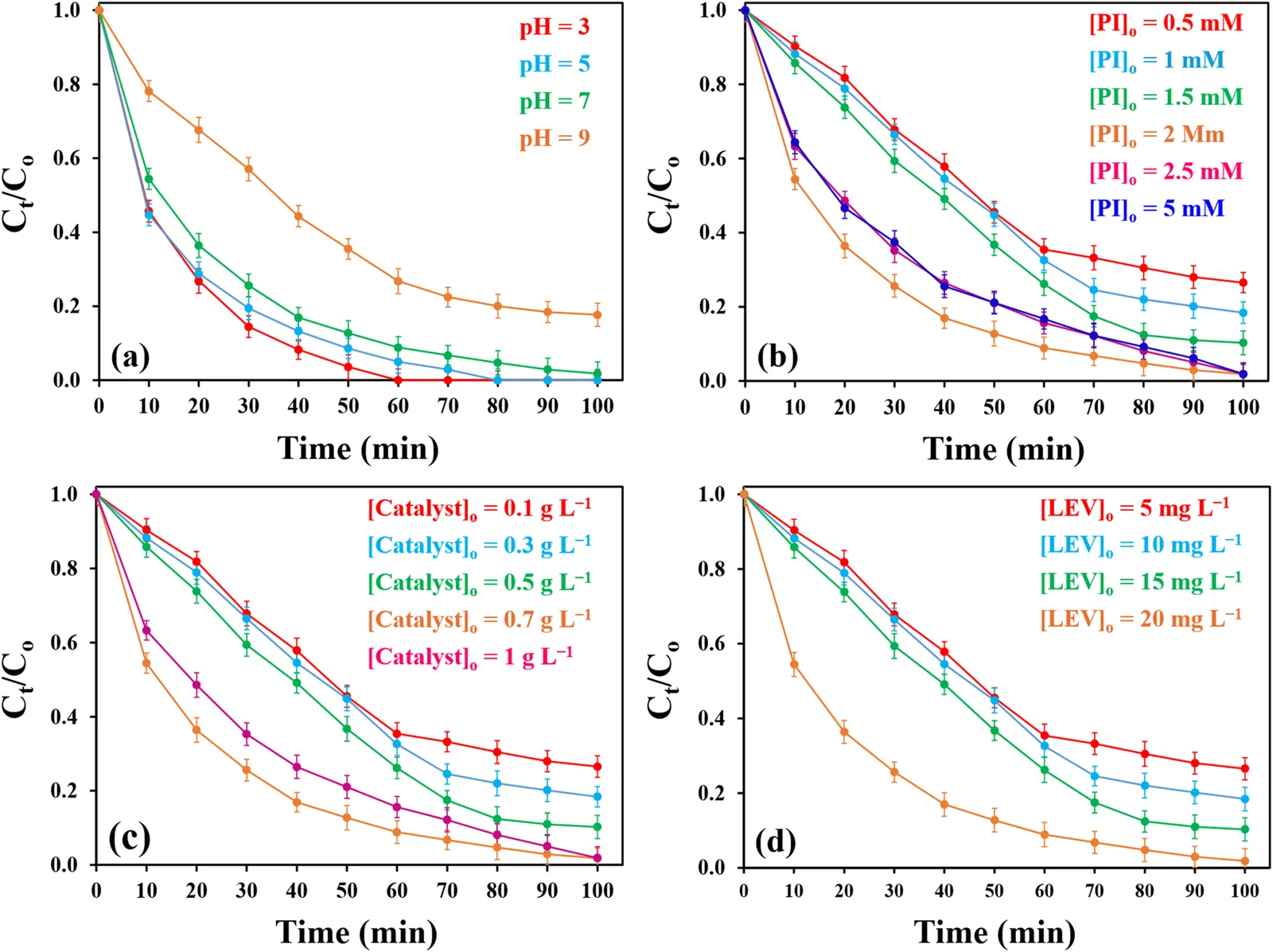
Effects of (a) initial solution pH, (b) initial PI concentration, (c) Ag/Ni-SDBC dosage, and (d) initial LEV concentration on LEV degradation in the Ag/Ni-SDBC/PI system. Conditions: [LEV]_o_ = 10 mg L^−1^, [PI]_o_ = 2 mM, [Ag/Ni-SDBC]_o_ = 0.5 g L^−1^, pH = 7, *T* = 25 °C, and reaction time = 100 min.

The role of PI concentration in LEV degradation within the Ag/Ni-SDBC/PI system was evaluated by varying the oxidant dosage from 0.5 to 5 mM while keeping all other operating conditions constant. As illustrated in Fig. 9(b) and summarized in Table S5, increasing the PI concentration from 0.5 to 2 mM substantially improved degradation performance from 73.47% to 98.2%, while the *k*_1_ value rose from 0.0145 to 0.04 min^−1^. The observed enhancement is likely due to the higher concentration of PI available for activation by the developed system, which facilitates the formation of reactive oxidizing species and consequently accelerates LEV degradation.^46^ Further increasing the PI concentration to 2.5 and 5 mM did not provide additional benefits. LEV removal efficiencies remained essentially unchanged, whereas the corresponding *k*_1_ values slightly declined to 0.034 and 0.0331 min^−1^. This behavior can be attributed to the excessive production of reactive species at elevated PI dosages, which favors radical–radical recombination reactions and leads to the formation of more stable, less reactive products that contribute negligibly to pollutant oxidation, as described in eqn (29–31).^47^ Moreover, excess PI may act as a radical scavenger, promoting competitive side reactions that consume 
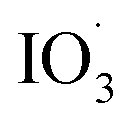
 and HO˙ radicals and reduce their availability for LEV degradation, as represented by eqn (3) and (4). Based on these findings, a PI concentration of 2 mM was identified as the optimum dosage for efficient LEV degradation in the Ag/Ni-SDBC/PI system.29
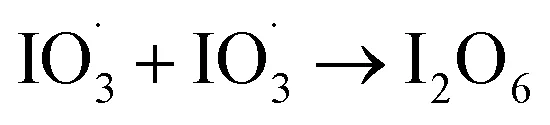
30
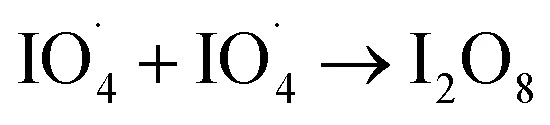

31 HO˙ + HO˙ → H_2_O_2_

The effect of Ag/Ni-SDBC dosage on LEV degradation in the developed system was evaluated over the range of 0.1–1.0 g L^−1^ (Fig. 9(c) and Table S5). Increasing the catalyst dosage from 0.1 to 0.5 g L^−1^ markedly improved the degradation performance, with the LEV removal efficiency raised from 63.63% to 98.2% and the *k*_1_ value increased from 0.0099 to 0.04 min^−1^. These findings highlight the crucial role of Ag/Ni-SDBC dosage in promoting PI activation and accelerating LEV degradation. The enhanced performance at higher catalyst loadings can be attributed to the larger accessible surface area, increased availability of active sites, and improved adsorption capacity, all of which facilitate more efficient PI activation and the generation of reactive species responsible for LEV oxidation.^41,60^ Further increasing the catalyst dosage to 0.7 and 1.0 g L^−1^ resulted in a decline in degradation efficiency to 86.93 and 81.87%, respectively. A similar trend was observed for the kinetic rate constants, which decreased to 0.0241 and 0.0202 min^−1^. The reduced catalytic performance at higher catalyst loadings can be attributed to particle aggregation, which diminishes the accessible surface area and the availability of active sites.^12,61^ Based on the combined consideration of degradation efficiency and reaction kinetics, a catalyst dosage of 0.5 g L^−1^ was selected as the optimum condition for subsequent experiments.

The influence of the initial LEV concentration on the degradation performance of the proposed system was evaluated over the concentration range of 5–20 mg L^−1^ (Fig. 9(d) and Table S5). The degradation efficiencies and kinetic rate constants decreased as the initial LEV concentration increased. The highest degradation performance was observed at an initial LEV concentration of 5 mg L^−1^, where complete pollutant removal was achieved within 60 min and a *k*_1_ value of 0.0682 min^−1^ was obtained. In contrast, increasing the LEV concentration to 10, 15, and 20 mg L^−1^ decreased its removal efficiency to 98.2%, 88.65%, and 74.14%, respectively, after 100 min of reaction. Correspondingly, the *k*_1_ values declined to 0.04, 0.0251, and 0.0169 min^−1^. The observed reduction in degradation performance at higher LEV concentrations can be attributed to the limited number of reactive species generated during PI activation, which becomes insufficient to efficiently oxidize the greater number of pollutant molecules. Furthermore, elevated LEV concentrations increase competition between LEV molecules and their degradation intermediates for both the available reactive species and the active sites on the catalyst surface. The accumulation of intermediate products may further impede mass transfer and partially block catalytic sites, thereby suppressing catalytic activity and slowing the overall degradation process.^34,62^ Although complete LEV degradation was attained at 5 mg L^−1^ within 60 min, an initial concentration of 10 mg L^−1^ was selected for subsequent experiments. This concentration provided a more rigorous assessment of the Ag/Ni-SDBC/PI system under relatively high pollutant loading while still maintaining excellent degradation performance (98.2% removal).

### 3.4. Performance comparison of Ag/Ni-SDBC/PI with recent catalytic systems

To highlight the advancements achieved in this study, Table 2 summarizes and compares the performance of the Ag/Ni-SDBC/PI system against existing pristine and functionalized sawdust-derived biochar materials utilized in oxidant-driven AOPs for the removal of antibiotics and pharmaceutical contaminants. The Ag/Ni-SDBC/PI system achieved 98.2% LEV removal within 100 min at neutral pH and room temperature using a catalyst dosage of 0.5 g L^−1^ and a PI concentration of 2 mM. This performance compares favorably with zerovalent iron-loaded sawdust biochar/PS, which required a substantially higher catalyst dosage (1.8 g L^−1^), a longer reaction time (120 min), and greater oxidant consumption (2 g L^−1^). Despite being evaluated at a lower initial pollutant concentration (5.6 mg L^−1^) and a slightly elevated reaction temperature (27 °C), the zerovalent iron-loaded sawdust biochar/PS system achieved a comparatively lower removal efficiency of only 93.2% for paracetamol.^63^ These operational requirements have important economic, environmental, and practical implications for large-scale implementation. The need for higher catalyst dosage and oxidant loading directly increases chemical consumption and operating costs, while the extended reaction time necessitates larger reactor volumes, thereby increasing capital and infrastructure expenditures. From an environmental perspective, the excessive use of chemical oxidants may result in the accumulation of residual inorganic species and the formation of secondary reaction by-products, potentially necessitating additional post-treatment processes prior to effluent discharge. Similarly, increasing the catalyst dosage increases material consumption and may generate larger quantities of spent catalyst that require recovery, regeneration, or disposal. Consequently, this can impose additional waste management challenges and increase the overall environmental and operational burden of the process. The longer treatment duration also contributes to higher energy consumption associated with mixing, pumping, and system operation. Furthermore, the superior performance of the reported system was achieved at a lower pollutant concentration and a slightly elevated temperature, both of which generally favor contaminant degradation kinetics and oxidant activation. Therefore, its lower removal efficiency under these comparatively advantageous conditions highlights the enhanced catalytic activity, oxidant utilization efficiency, and reaction kinetics of the developed Ag/Ni-SDBC/PI system. Collectively, these benefits translate into reduced operational expenditures, decreased chemical and energy demands, a lower environmental footprint, and improved process scalability, highlighting the potential of the current system for wastewater treatment applications. Although the molybdate-doped magnetic sawdust biochar/PS system achieved a comparable degradation efficiency (98.1%) for sulfamethazine at a similar oxidant demand (2 mM) and a higher initial pollutant concentration (20 mg L^−1^), its operation required acidic conditions (pH 5.5) and a longer reaction time (120 min), despite employing a lower catalyst dosage (0.1 g L^−1^).^64^ The higher pollutant concentration employed in the reported study is noteworthy, as elevated contaminant loadings generally increase oxidant demand and competition for reactive species, thereby making degradation more challenging. Consequently, the ability of the system to maintain high pollutant removal under these conditions highlights its strong oxidative capacity and effective utilization of reactive species. Nevertheless, the present system maintains a notable practical advantage by achieving efficient degradation under neutral pH conditions without the need for pH adjustment. This eliminates acid addition and subsequent neutralization steps, thereby reducing chemical consumption, operational costs, and equipment corrosion, and overall process complexity. These findings demonstrate that the Ag/Ni-SDBC/PI system combines high degradation efficiency with operationally favorable conditions, highlighting its potential for sustainable pharmaceutical wastewater treatment. The potassium-doped magnetic sawdust-derived biochar/PS system achieved a comparable removal efficiency (98.4%) for metronidazole under the same catalyst dosage (0.5 g L^−1^); however, it required a longer reaction time (180 min) despite operating at a higher initial pollutant concentration (20 mg L^−1^) and a lower oxidant dosage (1 mM).^65^ In contrast, the Ag/Ni-SDBC/PI system attained a close pollutant removal efficiency within only 100 min, demonstrating markedly faster degradation kinetics. Likewise, cobalt–nitrogen co-embedded hierarchical porous sawdust biochar activated PMS efficiently, achieving 97% chloroquine phosphate removal at a pollutant concentration of 10 mg L^−1^ using 1 mM oxidant and 0.2 g L^−1^ catalyst within 45 min.^66^ While this system exhibited faster degradation kinetics and required lower oxidant and catalyst dosages, the Ag/Ni-SDBC/PI system achieved a slightly higher removal efficiency under the same pollutant concentration and temperature conditions. Xu *et al.* demonstrated the efficient activation of H_2_O_2_ using aluminum-doped magnetic sawdust biochar, achieving 99.7% sulfamethazine removal at an initial pollutant concentration of 20 mg L^−1^ with an oxidant dosage of 1 mM and a catalyst loading of 0.8 g L^−1^ under acidic conditions (pH 5.5) within 120 min.^27^ Despite treating a lower pollutant concentration with a higher oxidant demand, the present system achieved efficient pollutant degradation under neutral pH conditions and within a considerably shorter treatment period, thereby eliminating the need for pH adjustment and enhancing its operational feasibility for wastewater treatment applications. Pristine sawdust-derived biochar demonstrated the ability to activate PMS for doxycycline degradation, achieving 90% removal at an initial pollutant concentration of 20 mg L^−1^ under neutral pH conditions within a reaction time of 40 min. However, achieving this performance necessitated an oxidant dosage 1.5 times higher (3 *vs.* 2 mM) and a catalyst loading six times greater (3 *vs.* 0.5 g L^−1^) than those employed in the present study.^67^ Although the present system required a longer reaction time and was evaluated at a lower pollutant concentration, its markedly superior degradation efficiency achieved with significantly reduced catalyst loading and oxidant dose highlights the enhanced catalytic activity of Ag/Ni-SDBC.

**Table 2:** Comparative evaluation of the Ag/Ni-SDBC/PI system with pristine and modified sawdust-derived biochar systems for the degradation of antibiotics and pharmaceutical contaminants *via* oxidant-based AOPs

Catalyst	Oxidant	Target pollutant	Operational conditions	Removal ratio (%)	Reference
Ag/Ni-SDBC	PI	LEV	[Pollutant]_o_ = 10 mg L^−1^, [Oxidant]_o_ = 2 mM, [Catalyst]_o_ = 0.5 g L^−1^, *T* = 25 °C, pH = 7, and reaction time = 100 min	98.2	This study
Zerovalent iron-loaded sawdust biochar	PS	Paracetamol	[Pollutant]_o_ = 5.6 mg L^−1^, [Oxidant]_o_ = 2 g L^−1^, [Catalyst]_o_ = 1.8 g L^−1^, *T* = 27 °C, pH = 6.5, and reaction time = 120 min	93.2	63
Molybdate-doped magnetic sawdust biochar	PS	Sulfamethazine	[Pollutant]_o_ = 20 mg L^−1^, [Oxidant]_o_ = 2 mM, [Catalyst]_o_ = 0.1 g L^−1^, *T* = 25 °C, pH = 5.5, and reaction time = 120 min	98.1	64
Potassium-doped magnetic sawdust-derived biochar	PS	Metronidazole	[Pollutant]_o_ = 20 mg L^−1^, [Oxidant]_o_ = 1 mM, [Catalyst]_o_ = 0.5 g L^−1^, *T* = 25 °C, and reaction time = 180 min	98.4	65
Cobalt–nitrogen co-embedded hierarchical porous sawdust biochar	PMS	Chloroquine phosphate	[Pollutant]_o_ = 10 mg L^−1^, [Oxidant]_o_ = 1 mM, [Catalyst]_o_ = 0.2 g L^−1^, *T* = 25 °C, and reaction time = 45 min	97	66
Aluminum-doped magnetic sawdust biochar	H_2_O_2_	Sulfamethazine	[Pollutant]_o_ = 20 mg L^−1^, [Oxidant]_o_ = 1 mM, [Catalyst]_o_ = 0.8 g L^−1^, *T* = 25 °C, pH = 5.5, and reaction time = 120 min	99.7	27
Pristine sawdust-derived biochar	PMS	Doxycycline	[Pollutant]_o_ = 20 mg L^−1^, [Oxidant]_o_ = 3 mM, [Catalyst]_o_ = 3 g L^−1^, pH = 7, and reaction time = 40 min	90	67

The Ag/Ni-SDBC/PI system also compares favorably with recently reported Ag-, Ni-, and biochar-based catalysts for PI activation and the degradation of various organic pollutants, as summarized in Table 3. A single-atom Ag porous tubular carbon nitride catalyst achieved 100% sulfamethoxazole removal within 25 min using 0.3 mM PI and 0.3 g L^−1^ catalyst. However, its performance was evaluated at a lower pollutant concentration of only 2 mg L^−1^ and required visible-light irradiation.^68^ In contrast, the Ag/Ni-SDBC/PI system operates without light irradiation and achieves high pollutant removal (98.2%) at a higher initial concentration of 10 mg L^−1^. Yao *et al.*^69^ achieved 100% complete removal of Orange II within 40 min using a manganese–nickel sulfide catalyst supported on graphite felt, with 0.65 mM PI and 20 mg L^−1^ Orange II. The nickel–manganese bimetallic sulfide grown *in situ* on nickel foam, achieved only 67.4% tetracycline hydrochloride removal after 120 min using a lower PI concentration of 1 mM and a higher initial pollutant concentration of 20 mg L^−1^.^70^ In comparison, the Ag/Ni-SDBC/PI system utilizes SDBC as a renewable carbonaceous support, promoting biomass waste valorization and offering potential cost advantages over graphite-based and metallic supports. Its comparable removal performance further highlights the potential of SDBC as a sustainable support for metal-based PI activation. The potassium ferrate modified biochar derived from waste wine lees biomass achieved complete tetracycline removal but required higher catalyst dosage (1.09 g L^−1^), higher PI concentration (3.29 mM), strong acidic conditions (pH 3), and longer reaction time (240 min).^71^ The magnetite nanoparticle supported on water lettuce-derived biochar achieved a slightly higher pollutant removal of 99.64% within 60 min at pH 7, however it required higher catalyst and PI dosages of 0.83 g L^−1^ and 2.05 mM, respectively.^57^ Similarly, Mulukhiya stalks-derived biochar achieved 99.7% sulfamethazine removal within 80 min, but at a higher PI concentration of 2.8 mM, despite using a lower catalyst dosage of 0.42 g L^−1^.^53^ Overall, the comparison demonstrates that although some catalysts outperform Ag/Ni-SDBC in individual parameters, the developed system combines high pollutant removal efficiency with moderate catalyst and PI requirements, neutral-pH operation, and a relatively short treatment time. These characteristics, together with the use of sawdust-derived biochar as a catalyst support, underscore its potential for practical application in antibiotic-contaminated wastewater treatment.

**Table 3:** Comparison of the catalytic performance of the proposed Ag/Ni-SDBC/PI system with recent Ag-, Ni-, and biochar-based PI activation systems for organic pollutant degradation

Catalyst	Target pollutant	Operational conditions	Removal ratio (%)	Reference
Ag/Ni-SDBC	LEV	[Pollutant]_o_ = 10 mg L^−1^, [PI]_o_ = 2 mM, [Catalyst]_o_ = 0.5 g L^−1^, *T* = 25 °C, pH = 7, and reaction time = 100 min	98.2	This study
Single-atom Ag porous tubular carbon nitride with N-vacancies/visible light	Sulfamethoxazole	[Pollutant]_o_ = 2 mg L^−1^, [PI]_o_ = 0.3 mM, [Catalyst]_o_ = 0.3 g L^−1^, and reaction time = 25 min	100	68
Nickel–manganese bimetallic sulfide grown *in situ* on nickel foam	Tetracycline hydrochloride	[Pollutant]_o_ = 20 mg L^−1^, [PI]_o_ = 1 mM, [Catalyst]_o_ = one piece, *T* = 25 °C, pH = not adjusted, and reaction time = 120 min	67.4	70
Manganese–nickel sulfide supported on graphite felt	Orange II	[Pollutant]_o_ = 20 mg L^−1^, [PI]_o_ = 0.65 mM, *T* = 25 °C, pH = 6.5, and reaction time = 40 min	100	69
Magnetite nanoparticles supported on water lettuce-derived biochar	Tetracycline	[Pollutant]_o_ = 16.52 mg L^−1^, [PI]_o_ = 2.05 mM, [Catalyst]_o_ = 0.83 g L^−1^, *T* = 25 °C, pH = 7, and reaction time = 60 min	99.64	57
Mulukhiyah stalks-derived biochar	Sulfamethazine	[Pollutant]_o_ = 6.7 mg L^−1^, [PI]_o_ = 2.8 mM, [Catalyst]_o_ = 0.42 g L^−1^, pH = 7, and reaction time = 80 min	99.7	53
Potassium ferrate-modified biochar derived from waste wine lees biomass	Tetracycline	[Pollutant]_o_ = 20.3 mg L^−1^, [PI]_o_ = 3.29 mM, [Catalyst]_o_ = 1.09 g L^−1^, pH = 3, and reaction time = 240 min	100	71

### 3.5. Influence of inorganic anions and natural organic matter

Due to the extensive presence of NOM, and inorganic anions (*e.g.* Cl^−^, NO_3_^−^, SO_4_^2−^, and HCO_3_^−^) in natural water bodies and wastewater matrices, their potential influence on LEV degradation in the Ag/Ni-SDBC/PI system was systematically evaluated. These coexisting constituents can interfere with oxidation processes through multiple pathways, such as scavenging reactive species, competing with target pollutants for catalytic active sites, and inhibiting oxidant activation, thereby diminishing the overall treatment performance.^44,72^ The experiments were conducted under the optimum conditions (neutral pH, Ag/Ni-SDBC dosage = 0.5 g L^−1^, PI concentration = 2 mM, and initial LEV concentration = 10 mg L^−1^) at room temperature for 100 min. The tested anions were individually introduced at concentrations of 1, 5, 10, and 20 mM, while of HA was examined at levels of 1, 5, 10, and 20 mg L^−1^. The presence of Cl^−^, NO_3_^−^, and SO_4_^2−^ anions resulted in a limited impact on the catalytic performance of the Ag/Ni-SDBC/PI system. Even at the highest investigated concentration (20 mM), LEV removal efficiencies remained above 94%, with only slight reductions in the corresponding reaction rates (Fig. 10 and Table S6). These results demonstrate the strong tolerance of the Ag/Ni-SDBC/PI system to interfere from these common inorganic anions and highlight its potential applicability in complex aqueous matrices. The minor suppression of LEV degradation observed after the introduction of these inorganic anions can be primarily attributed to their radical-scavenging behavior. By reacting with highly reactive species, these anions generate less reactive species, leading to a reduction in the overall oxidative capacity of the system (Table S7 and eqn (1)–(12)). In addition, these anions may compete with LEV for catalyst surface sites, thereby reducing pollutant adsorption and limiting access to active sites.^54^ Moreover, the accumulation of inorganic anions on the catalyst surface may lead to the formation of a thin passivation layer, which can hinder mass transfer between the catalyst, oxidant, and pollutant molecules, thereby reducing the efficiency of oxidant activation.^59^ In contrast, HCO_3_^−^ anions exhibited a significant inhibitory effect on LEV degradation, particularly at higher concentrations. Increasing the concentration of HCO_3_^−^ anions from 1 to 20 mM reduced the LEV removal efficiency from 90.09% to 69.07%, accompanied by a marked decline in the reaction rate constant from 0.0243 to 0.0129 min^−1^. The inhibitory effect of HCO_3_^−^ anions can be primarily associated with its ability to quench highly reactive species, such as HO˙ radicals, 
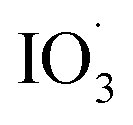
 radicals, and ^1^O_2_. These interactions promote the conversion of the reactive species into carbonate radicals (CO_3_˙^−^) and bicarbonate radicals 
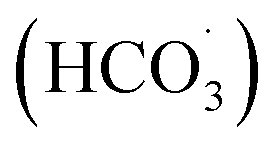
 (Table S7 and eqn (13)–(17)), which possess markedly lower redox potential, ultimately diminishing the degradation performance of the Ag/Ni-SDBC/PI system. At elevated concentrations of HCO_3_^−^ anions, the generated CO_3_˙^−^ radicals are susceptible to further transformation through self-recombination and reactions with H_2_O molecules (Table S7, eqn (18) and (19)). These secondary pathways lead to the depletion of CO_3_˙^−^ radicals and the formation of species with lower oxidative reactivity. Moreover, HCO_3_^−^ anions may interfere with the catalytic activation of PI through interactions with catalytically active sites, promoting the formation of aqueous complexes or precipitated species that hinder reactive species generation and decrease their participation in the degradation process.^52,73^ These findings underscore the importance of removal or reducing HCO_3_^−^ levels before treatment to alleviate its inhibitory effects and preserve the degradation efficiency of the Ag/Ni-SDBC/PI system.

**Fig. 10:**
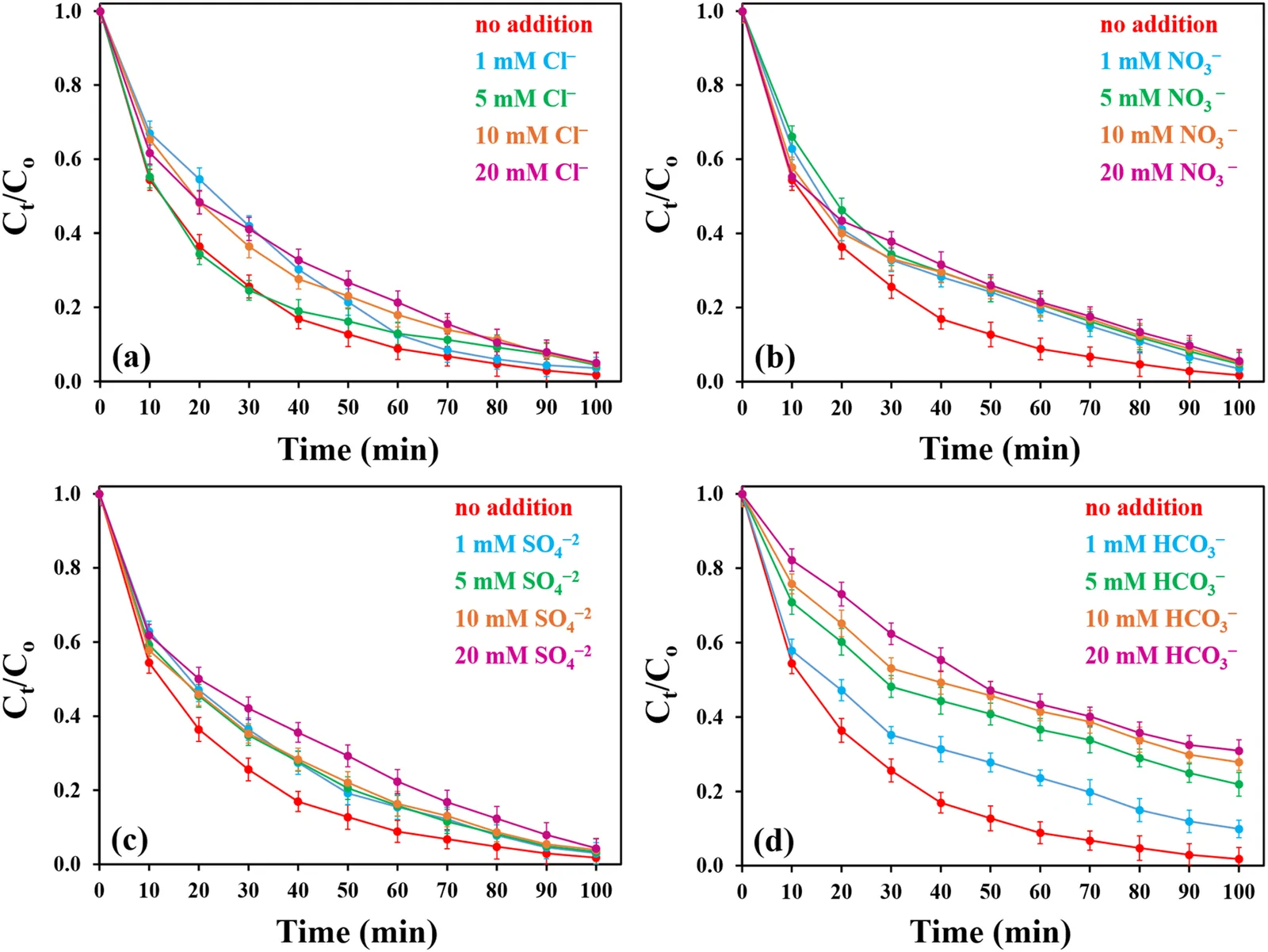
Influence of inorganic anions at different concentrations (1, 5, 10, and 20 mM) on LEV degradation in the Ag/Ni-SDBC/PI system: (a) Cl^−^, (b) NO_3_^−^, (c) SO_4_^2−^, and (d) HCO_3_^−^. Experimental conditions: [LEV]_o_ = 10 mg L^−1^, [PI]_o_ = 2 mM, [Ag/Ni-SDBC]_o_ = 0.5 g L^−1^, pH = 7, *T* = 25 °C, and reaction time = 100 min.

As shown in Fig. 11(a) and Table S6, the addition of HA at a concentration of 1 mg L^−1^ resulted in a slight decrease in LEV removal efficiency from 98.2% to 95.06%, accompanied by a reduction in the *k*_1_ values from 0.04 to 0.0278 min^−1^. Interestingly, further increases in HA concentration gradually restored the degradation performance. The LEV removal efficiency increased to 97.82%, 98.06%, and 98.83% at HA concentrations of 5, 10, and 20 mg L^−1^, respectively, accompanied by corresponding increases in the *k*_1_ values to 0.0334, 0.0378, and 0.0377 min^−1^. These results demonstrate that the Ag/Ni-SDBC/PI system exhibits strong resistance to NOM interference and can maintain high catalytic activity across the investigated concentration range. The negligible impact of HA on LEV degradation in the Ag/Ni-SDBC/PI system can be attributed to its dual role during the oxidation process. On one hand, HA may inhibit degradation by scavenging reactive species and competing with LEV for catalytic active sites.^13,74^ On the other hand, HA can exert a promotional effect. The aromatic functional groups within HA may interact with the quinolone structure of LEV through π–π interactions, facilitating electron transfer and enhancing the susceptibility of LEV to oxidative degradation.^58^

**Fig. 11:**
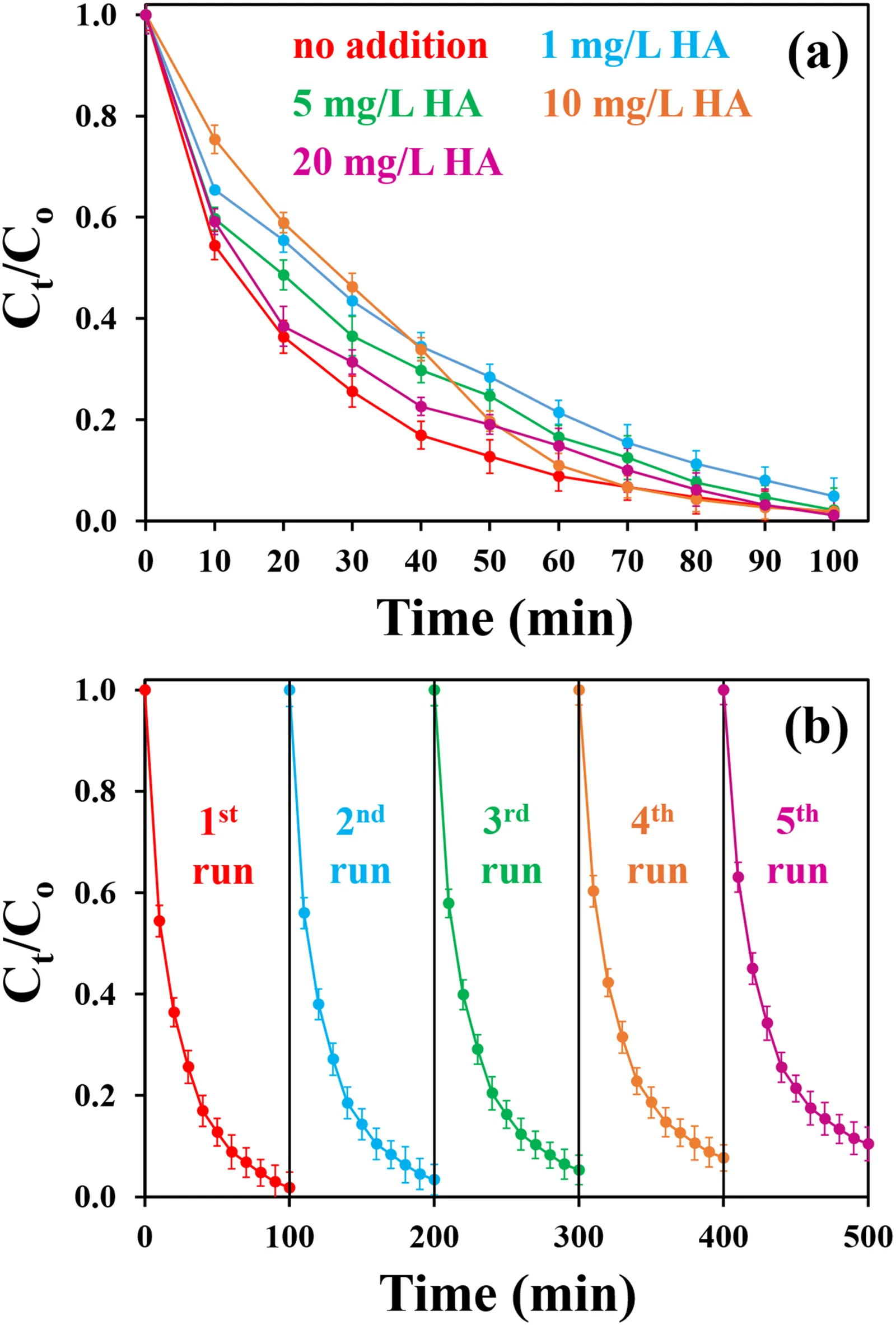
Assessment of the Ag/Ni-SDBC/PI system for (a) LEV degradation in the presence of different concentrations of HA (1, 5, 10, and 20 mg L^−1^) and (b) catalyst stability during five consecutive reuse cycles. Conditions: [LEV]_o_ = 10 mg L^−1^, [PI]_o_ = 2 mM, [Ag/Ni-SDBC]_o_ = 0.5 g L^−1^, pH = 7, *T* = 25 °C, and reaction time = 100.

### 3.6. Fate of iodine species during LEV degradation in the Ag/Ni-SDBC/PI system

A key concern associated with PI-based AOPs is the potential generation of reactive iodine species (*e.g.* HOI, I^−^, I_2_, and I_3_^−^), during the degradation of organic compounds. These species are environmentally significant because they can react with organic matter to form iodinated disinfection by-products, which are often highly toxic, persistent, and resistant to biodegradation. Moreover, exposure to such compounds has been linked to cytotoxic, genotoxic, and carcinogenic effects, raising concerns regarding their environmental and public health impacts.^75,76^ To evaluate the fate of iodine species in the Ag/Ni-SDBC/PI system, the concentrations of IO_4_^−^ ions, IO_3_^−^, HOI, I^−^, I_2_, and I_3_^−^ were monitored during a 100 min treatment period at room temperature using an initial PI concentration of 2 mM, a catalyst dosage of 0.5 g L^−1^, an initial LEV concentration of 10 mg L^−1^, and neutral pH conditions. As shown in Fig. S7, the gradual consumption of IO_4_^−^ ions during the reaction was accompanied by a nearly stoichiometric accumulation of IO_3_^−^ ions. In contrast, HOI, I^−^, I_2_, or I_3_^−^ remained below the detection limits throughout the reaction. These results indicate that PI activation in the Ag/Ni-SDBC/PI system proceeds primarily through a selective reduction pathway that favors the formation of IO_3_^−^ ions, a stable iodine species with minimal environmental concern.^77^ The absence of detectable HOI, I^−^, I_2_, or I_3_^−^ further demonstrates the environmental compatibility of the Ag/Ni-SDBC/PI process, with promising potential for scale-up and practical deployment.

### 3.7. Evaluation of the cyclic performance of the treatment system

The operational reusability of the Ag/Ni-SDBC/PI system were assessed through five consecutive cycling experiments conducted under the optimum reaction conditions ([PI]_o_ = 2 mM, [catalyst]_o_ = 0.5 g L^−1^, [LEV]_o_ = 10 mg L^−1^, and neutral pH). Each cycle was performed at room temperature with a reaction duration of 100 min. As shown in Fig. 11(b) and summarized in Table S8, the Ag/Ni-SDBC/PI system maintained a high LEV removal efficiency of 89.57% after five successive cycles, corresponding to an efficiency loss of only 8.79% relative to the initial removal efficiency of 98.2%. In addition, the *k*_1_ value decreased from 0.04 to 0.026 min^−1^ over the same period. The sustained degradation performance over successive cycles highlights the recyclability of the catalyst and its ability to maintain effective catalytic performance during repeated use. Moreover, the reusability performance of Ag/Ni-SDBC compared favorably with those of numerous pristine and modified sawdust-derived biochars and other lignocellulosic biochar-based catalysts utilized in different oxidant-driven AOPs for the degradation of antibiotics and pharmaceutical contaminants (Table 4).

**Table 4:** Comparison of the recyclability performance of Ag/Ni-SDBC with previously reported catalysts for the degradation of antibiotics and pharmaceutical contaminants *via* different oxidant-based AOPs

Catalyst/System	Target pollutant	Reusability runs	Initial pollutant RE (%)	Final pollutant RE (%)	Efficiency loss (%)	Reference
Ag/Ni-SDBC/PI	LEV	5	98.2	89.57	8.79	This study
Fe^0^-SDB/PS^a^	Paracetamol	3	93.02	88	5.58	63
Co-N@BC/PMS^b^	Chloroquine phosphate	4	97	∼90	∼7.22	66
BC/PMS^c^	Doxycycline	5	90	80.96	10.04	67
WGBC/PS^d^	Sulfamethoxazole	3	98	25	74.49	78
GBC/PS^e^	Tetracycline	3	78.7	55.2	29.86	79
NBC/PMS^f^	Pefloxacin	5	93.23	80.67	13.47	80
MMBC/PS^g^	Tetracycline	4	93.2	∼75	∼19.53	81

a Fe^o^-SDB: zerovalent iron-loaded sawdust biochar.

b Co-N@BC: cobalt-nitrogen co-embedded hierarchical porous sawdust biochar.

c BC: sawdust-derived biochar.

d WGBC: graphitized biochar derived from wood chips.

e GBC: graphitic biochar derived from wood chips.

f NBC: N-doped moso bamboo biochar.

g MMBC: Mn-doped magnetic biochar derived from bamboo residues.

These results further demonstrate the long-term recyclability of the Ag/Ni-SDBC catalyst during repeated operation. As presented in Table S9, the Ag/Ni-SDBC catalyst exhibited a gradual mass loss during the repeated operation cycles. The catalyst dosage decreased from 0.5 g L^−1^ in the first cycle to 0.3735 g L^−1^ after the fifth cycle, corresponding to a cumulative mass loss ratio of 25.3%. Such a decrease in catalyst loading could reduce the number of available active sites participating in the PI activation process, thereby contributing to the observed decline in catalytic performance during repeated operation.^78^ Furthermore, the accumulation of LEV molecules and their degradation intermediates on the catalyst surface may have progressively blocked or fouled active sites, limiting their accessibility and further impairing catalytic activity.^43^ Besides, metal leaching from the Ag/Ni-SDBC catalyst was assessed to evaluate its recyclability during repeated operation. As shown in Table S9, the highest concentrations of leached Ag and Ni were detected after the fifth cycle, reaching 0.0538 and 0.0123 mg L^−1^, respectively. These values remained well below the maximum permissible limits established by the World Health Organization (WHO) for drinking water (0.1 mg L^−1^ for Ag and 0.07 mg L^−1^ for Ni).^79^ The relatively limited extent of metal release indicates the strong immobilization of the metal species within the biochar matrix of the bimetallic catalyst throughout repeated use. Furthermore, the low concentrations of leached metals indicate a minimal risk of secondary contamination, underscoring the environmental safety and practical applicability of the Ag/Ni-SDBC catalyst for sustainable wastewater treatment.

### 3.8. Generation of reactive species and possible PI activation mechanism

As discussed previously, the Ag/Ni-SDBC/PI system can promote the production of diverse ROS (HO^•^ radicals, O_2_˙^−^ radicals, and ^1^O_2_) and RIS (
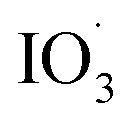
 and 
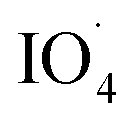
 radicals), thereby establishing a highly oxidative environment conducive to efficient LEV degradation. Identifying the predominant reactive species is crucial for elucidating the underlying degradation mechanism. Therefore, a series of scavenging experiments using selective quenchers were performed under the optimum reaction conditions (an initial PI concentration of 2 mM, a catalyst loading of 0.5 g L^−1^, neutral Ph, and an initial LEV concentration of 10 mg L^−1^) at room temperature over a 100 min treatment period. Phenol was used as a selective scavenger to investigate the overall contribution of HO˙, 
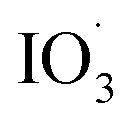
 and 
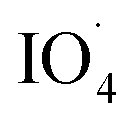
 radicals to the oxidation of LEV by simultaneously inhibiting their reactivity.^80^ TBA, which reacts rapidly with HO˙ radicals (quenching rate constant (*k*_q_) = 6 × 10^8^ M^−1^ s^−1^), was used as a selective quencher of HO˙ radicals.^40,47^*P*-BQ was utilized to capture O_2_˙^−^ radicals, with a reported *k*_q_ value of 9.8 × 10^8^ M^−1^ s^−1^.^47^ The involvement of singlet oxygen ^1^O_2_ was investigated using FFA and l-His, which exhibit *k*_q_ values of 1.2 × 10^8^ and 3.2 × 10^7^ M^−1^ s^−1^, respectively.^40^

As illustrated in Fig. 12(a) and Table S10, the addition of TBA at concentrations of 10 and 100 mM partially suppressed LEV degradation, decreasing the removal efficiency from 98.2% (in case of no scavenger) to 68.36% and 62.63%, corresponding to losses in removal efficiency of 30.39% and 36.22%, respectively. Correspondingly, the *k*_1_ values declined from 0.04 min^−1^ (without scavenger) to 0.0137 and 0.0115 min^−1^, respectively. These results indicate that HO˙ radicals participate in LEV degradation within the Ag/Ni-SDBC/PI system. Moreover, the addition of phenol significantly suppressed LEV degradation, reducing the removal efficiency to 34.51%, 20.65%, and 15.79% at phenol concentrations of 1, 5, and 10 mM, corresponding to losses in removal efficiency of 64.86%, 79.06%, and 83.92%, respectively. Correspondingly, the *k*_1_ value decreased to 0.0048, 0.0028, and 0.0020 min^−1^, respectively (Fig. 12(a); Table S10). A noteworthy finding is that the extent of inhibition observed with phenol was substantially greater than that obtained with excess TBA, demonstrating that 
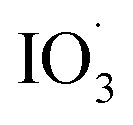
 and 
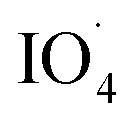
 radicals may also participate in LEV degradation process within the Ag/Ni-SDBC/PI system. According to eqn (32), IO_3_^−^ ions can be generated during the oxidation of LEV by 
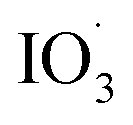
 radicals.^11^ Meanwhile, the interaction between 
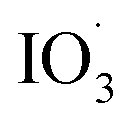
 radicals and IO_4_^−^ ions can lead to the formation of 
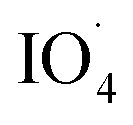
 radicals, as described in eqn (3). Consequently, the formation of IO_3_^−^ ions can serve as indirect evidence for the contribution of 
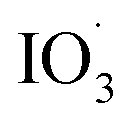
 radicals to LEV degradation, while simultaneously indicating the participation of 
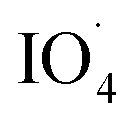
 radicals formed from their conversion. As shown in Fig. S7, the gradual depletion of IO_4_^−^ ions was accompanied by the concurrent accumulation of IO_3_^−^ ions in a nearly stoichiometric manner, supporting the generation of both 
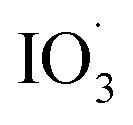
 and 
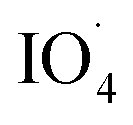
 radicals in the Ag/Ni-SDBC/PI system.32
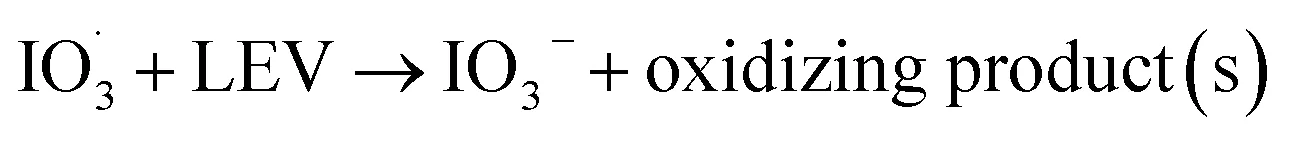


**Fig. 12:**
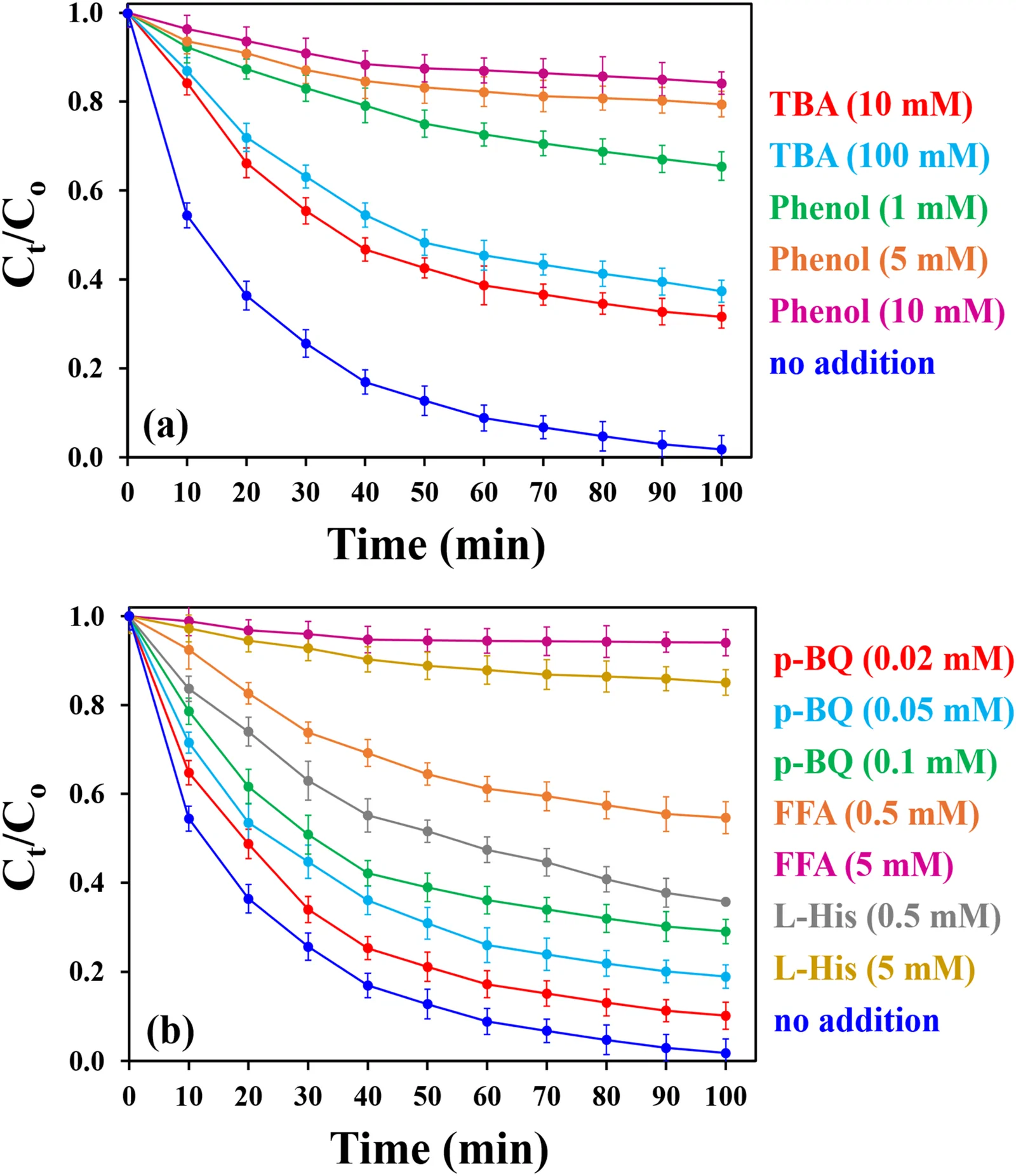
(a) and (b) Impacts of radical scavengers on LEV degradation in the Ag/Ni-SDBC/PI system. Experimental conditions: [LEV]_o_ = 10 mg L^−1^, [PI]_o_ = 2 mM, [Ag/Ni-SDBC]_o_ = 0.5 g L^−1^, pH = 7, *T* = 25 °C, and reaction time = 100 min.

The introduction of *p*-BQ at concentrations of 0.02, 0.05, and 0.1 mM resulted in only a moderate decline in LEV degradation efficiency, with LEV removal decreasing to 89.85%, 81.08%, and 70.98%, reflecting decreases of 8.5%, 17.43%, and 27.72%, respectively. Correspondingly, *k*_1_ values decreased to 0.0262, 0.0196, and 0.0149 min^−1^ (Fig. 12(b) and Table S10). The relatively weak inhibitory effect of *p*-BQ indicates that O_2_˙^−^ radicals were generated only to a limited extent in the Ag/Ni-SDBC/PI system. Furthermore, FFA exhibited a concentration-dependent inhibitory effect on LEV degradation. The addition of 0.5 mM FFA led to a moderate reduction in the removal efficiency to 45.37%, equivalent to a 53.8% decrease, whereas increasing the FFA concentration to 5 mM markedly suppressed LEV degradation, decreasing the removal efficiency to only 5.94%, corresponding to a 93.95% decrease. These changes were accompanied by pronounced decreases in the *k*_1_ values to 0.0071 and 0.0008 min^−1^, respectively (Fig. 12(b) and Table S10). A similar trend was observed for l-His, with LEV removal efficiencies declining to 64.28% and 14.92% in the presence of 0.5 and 5 mM l-His, corresponding to decreases of 34.54% and 84.81%, respectively. Likewise, the corresponding *k*_1_ values decreased to 0.0115 and 0.0019 min^−1^, respectively (Fig. 12(b) and Table S10). These results clearly indicate that ^1^O_2_ was the predominant reactive species responsible for LEV degradation in the Ag/Ni-SDBC/PI system. Numerous metal-loaded biochar/PI systems studies have also identified ^1^O_2_ as the primary oxidizing species responsible for the degradation of organic contaminants.^81–83^

Elucidating the generation and transformation pathways of reactive species is crucial for gaining a comprehensive understanding of the reaction mechanisms and PI activation in the Ag/Ni-SDBC/PI system. The generated H_2_O_2_ can serve as an additional precursor that contributes to the generation of several reactive species in the Ag/Ni-SDBC/PI system, including HO˙ radicals and ^1^O_2_ (eqn (16)), 
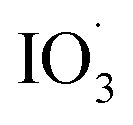
 and O_2_˙^−^ radicals (eqn (17)), and HO˙ radicals (eqn (24)). Several possible pathways for H_2_O_2_ generation have been proposed based on previously reported reactions As described in eqn (11), the reaction of O_2_˙^−^ radicals with H_2_O molecules can produce both ^1^O_2_ and H_2_O_2_. In addition, O_2_˙^−^ radicals undergo protonation to form hydroperoxyl radicals 
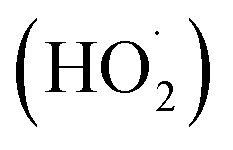
 (eqn (11)). These 
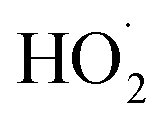
 radicals may either recombine to generate H_2_O_2_ and O_2_ (eqn (13)) or react with O_2_˙^−^ radicals to produce hydroperoxyl ions (HO_2_^−^) (eqn (14)), which can be subsequently protonated to yield additional H_2_O_2_ (eqn (15)). The oxidation states of Ag and Ni may be varied during their reactions with periodate and the generated reactive species.^84^ Further, possible electron exchange between Ag and Ni may facilitate fluctuations in their oxidation states.^85^ Thus, the redox cycling of Ag and Ni can promote electron transfer, which may facilitate the reduction of dissolved O_2_ in the presence of H^+^ ions, providing another route for H_2_O_2_ formation (eqn (23)). The recombination of highly reactive HO˙ radicals can also contribute to H_2_O_2_ production (eqn (31)). These reactions represent proposed mechanistic pathways based on previously reported literature and were not individually verified experimentally in the present study. However, the ability of Ag/Ni-SDBC to generate H_2_O_2_ was experimentally verified by monitoring H_2_O_2_ formation in 100 mL of deionized water in the absence of both the target pollutant and PI. As shown in Fig. S8, the H_2_O_2_ concentration increased progressively with reaction time, providing direct experimental evidence for the continuous *in situ* generation of H_2_O_2_ by the Ag/Ni-SDBC system.

To elucidate the contribution of dissolved O_2_ to the generation of O_2_˙^−^ radicals in the Ag/Ni-SDBC/PI system (eqn (22)), the reaction solution was purged with high-purity nitrogen gas for 30 min prior to reaction and continuously maintained under N_2_ purging conditions throughout the reaction to eliminate dissolved O_2_. Under these conditions, the LEV degradation efficiency decreased from 98.2% under ambient air conditions to 77.65% (Fig. S9), providing experimental evidence that dissolved O_2_ contributes to the generation of O_2_˙^−^ radicals. In contrast, continuously purging the solution with high purity oxygen gas produced only a slight improvement in LEV degradation despite the increased availability of dissolved oxygen (Fig. S9). This limited enhancement can be attributed to competition between dissolved O_2_ and IO_4_^−^ ions for reduction during the redox cycling of Ag and Ni. Besides reducing dissolved O_2_ to O_2_˙^−^ radicals, reduced Ag and Ni metals can also activate PI to generate 
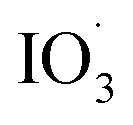
 radicals (eqn (21)). Therefore, although elevated O_2_ concentrations favor the production of O_2_˙^−^ radicals, they may simultaneously divert electrons away from PI reduction, thereby suppressing the formation of 
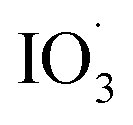
 radicals. The proposed competition between dissolved O_2_ and IO_4_^−^ ions for electron transfer during Ag/Ni redox cycling is based on the established redox reactions reported in the literature. As a result, the beneficial effect of the increased generation of O_2_˙^−^ radicals is largely counterbalanced by the diminished production of 
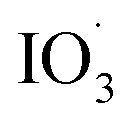
 radicals, leading to only a marginal increase in LEV degradation efficiency. These observations suggest that 
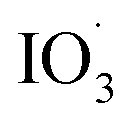
 radicals play a more dominant role than O˙^−^ radicals in LEV oxidation within the Ag/Ni-SDBC/PI system, consistent with the radical quenching results. To further clarify the origin of O_2_˙^−^ radicals in the Ag/Ni-SDBC/PI system, 10 mM *p*-BQ, a selective scavenger for O_2_˙^−^ radicals, was added under nitrogen-purged conditions. The introduction of *p*-BQ led to a further decrease in LEV degradation efficiency, from 77.65% in the nitrogen-purged system to 56.68%, experimentally confirming that O_2_˙^−^ radicals were still generated and involved in LEV degradation even in the absence of dissolved O_2_. This observation suggests that, in addition to the reduction of dissolved O_2_ during the redox cycling of Ag and Ni (eqn (22)), alternative pathways can also contribute to the formation of O_2_˙^−^ radicals, as outlined in eqn (5), (6), (17), and (25).

The predominance of ^1^O_2_ in the Ag/Ni-SDBC/PI system can be attributed to the presence of diverse concurrent routes for its generation. The formation of ^1^O_2_ is closely linked to the generation and subsequent transformation of O_2_˙^−^ radicals. As described in eqn (10), (11), and (16), O_2_˙^−^ radicals can react with HO˙ radicals, H_2_O molecules, and H_2_O_2_ to produce ^1^O_2_. An additional route involves the reaction between O_2_˙^−^ radicals and IO_4_^−^ ions (eqn (7)). However, according to Ling *et al.*, the conversion of O_2_˙^−^ radicals into ^1^O_2_ through eqn (10), (11), and (16) is thermodynamically more favorable than its direct reaction with IO_4_^−^ ions.^86^ Furthermore, ^1^O_2_ can also be produced through the self-reaction of PI ions, as represented by eqn (8). These pathways are inferred from previously reported reactions in the literature and were not independently validated in the present study. Experimentally, the dominant role of ^1^O_2_ was supported by the quenching experiments (Fig. 12(b)). Moreover, the proposed interconversion between ^1^O_2_ and O_2_˙^−^ radicals (eqn (25)) provides a possible explanation for the continued generation of O_2_˙^−^ radicals, although this specific pathway remains literature-based.

The generation of 
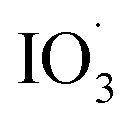
 radicals in the Ag/Ni-SDBC/PI system can occur through several parallel processes. One important route involves the activation of PI by the oxygen-containing functional groups on SDBC, as described in eqn (1) and (2). In addition, 
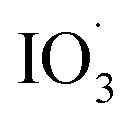
 radicals can be formed through the oxidation of IO_3_^−^ ions by HO˙ radicals (eqn (9)), as well as through the reaction between IO_4_^−^ ions and H_2_O_2_ (eqn (17)). IO_4_^−^ ions can generate 
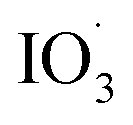
 radicals through an electron transfer pathway in which PI can act as an electron acceptor during the redox cycling of Ag and Ni, resulting in the formation of additional 
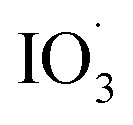
 radicals (eqn (21)). The coexistence of these complementary pathways is expected to sustain a relatively high concentration of 
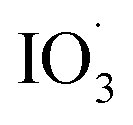
 radicals throughout the reaction, contributing significantly to the overall oxidative performance of the Ag/Ni-SDBC/PI system. On the other hand, 
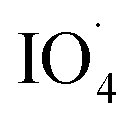
 radicals in the Ag/Ni-SDBC/PI system can be generated through the reaction of PI with 
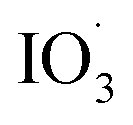
 radicals and HO˙ radicals, as described in eqn (3) and (4), respectively. These reaction pathways were proposed based on established mechanisms reported in the literature and were not independently validated in this study. However, the detection of IO_3_^−^ accompanied by the consumption of IO_4_^−^ (Fig. S7) provides experimental evidence supporting PI reduction and the formation of iodine-derived reactive intermediates, indirectly supporting the proposed generation of 
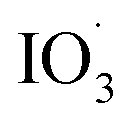
 and 
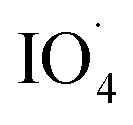
 radicals. The coexistence of these pathways may sustain the formation of reactive iodine species and contribute to the oxidative performance of the Ag/Ni-SDBC/PI system.

Based on the above findings, a plausible mechanism for LEV degradation by the Ag/Ni-SDBC/PI system is proposed, as depicted in Fig. 13. The degradation of LEV in the Ag/Ni-SDBC/PI system is governed by the effects of multiple ROS, including ^1^O_2_, HO˙ radicals, and O_2_˙^−^ radicals, together with RIS (
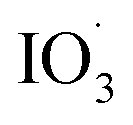
 and 
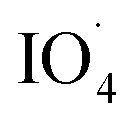
 radicals). These oxidizing species initiate the degradation process by attacking LEV molecules and converting them into smaller organic intermediates through a series of oxidation reactions. The resulting intermediates subsequently undergo further oxidative transformations, leading to progressive molecular fragmentation and structural breakdown, and ultimately to complete mineralization into CO_2_ and H_2_O (eqn (33)).^11^33
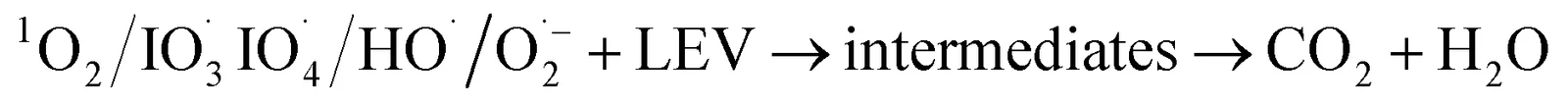


**Fig. 13:**
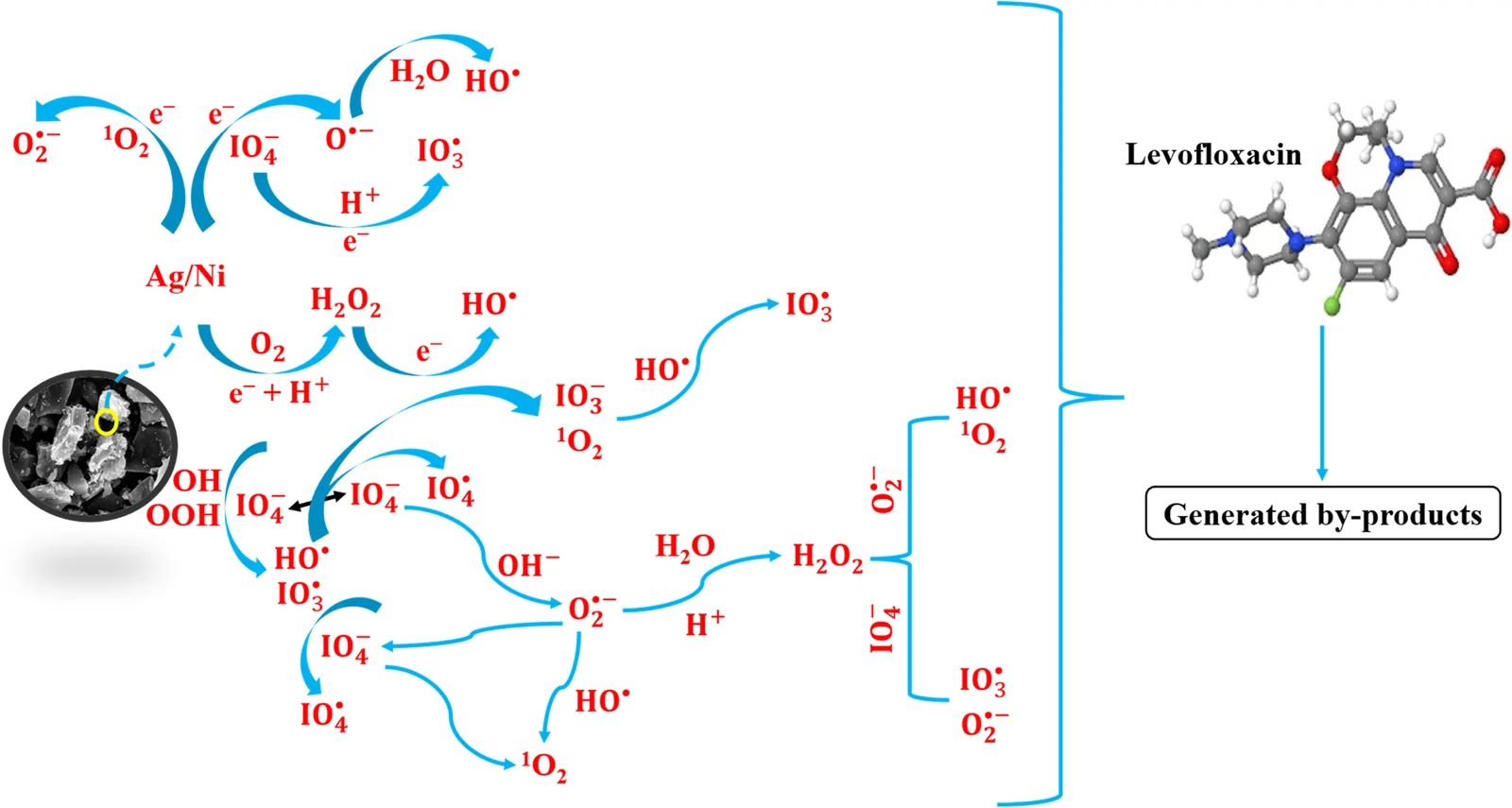
Proposed LEV degradation mechanism in the Ag/Ni-SDBC/PI system.

### 3.9. Degradation behavior of different antibiotics in the proposed system

To assess the versatility of the Ag/Ni-SDBC/PI system, four additional antibiotics (CAP, SMX, TC, and CIP) were selected as representative target compounds. These antibiotics exhibit distinct molecular structures and physicochemical characteristics, enabling a comprehensive evaluation of the developed system's applicability to a broad range of pharmaceutical pollutants. The experiments were conducted under the following experimental conditions: room temperature, neutral pH, catalyst dosage = 0.5 g L^−1^, PI concentration = 2 mM, and initial antibiotic concentration = 10 mg L^−1^. As shown in Fig. 14(a) and Table S11, removal efficiencies of 70.88%, 75.23%, 83.96%, and 91.29% were obtained for CAP, SMX, TC, and CIP, respectively, corresponding to *k*_1_ values of 0.0156, 0.0153, 0.0214, and 0.0124 min^−1^. Under the same conditions, LEV exhibited the highest degradation performance, with a removal efficiency of 98.2% and a rate constant of 0.04 min^−1^. Despite being optimized for LEV degradation, the Ag/Ni-SDBC/PI system demonstrated considerable efficacy toward the degradation of several structurally diverse antibiotics, highlighting the system's potential as an effective advanced oxidation technology for treating antibiotic-contaminated wastewater. Further optimization of the reaction conditions for CAP, SMX, TC, and CIP may lead to higher removal efficiencies and enhanced degradation kinetics and. In addition to pollutant degradation, the mineralization performance of the Ag/Ni-SDBC/PI system was evaluated through TOC analysis. The TOC removal efficiencies achieved for CAP, SMX, TC, and CIP were 41.28%, 48.43%, 53.85%, and 64.57%, respectively, whereas LEV exhibited a markedly higher TOC removal efficiency of 76.33% (Table S11). For all investigated antibiotics, TOC removal remained lower than the corresponding pollutant degradation efficiency. This phenomenon is commonly observed in AOPs, where the initial breakdown of target contaminants generates a range of partially oxidized intermediates that persist in solution and contribute to the residual organic carbon content.^87^ Nevertheless, the appreciable TOC reductions obtained for all tested antibiotics demonstrate the ability of the Ag/Ni-SDBC/PI system to achieve both pollutant degradation and partial mineralization.

**Fig. 14:**
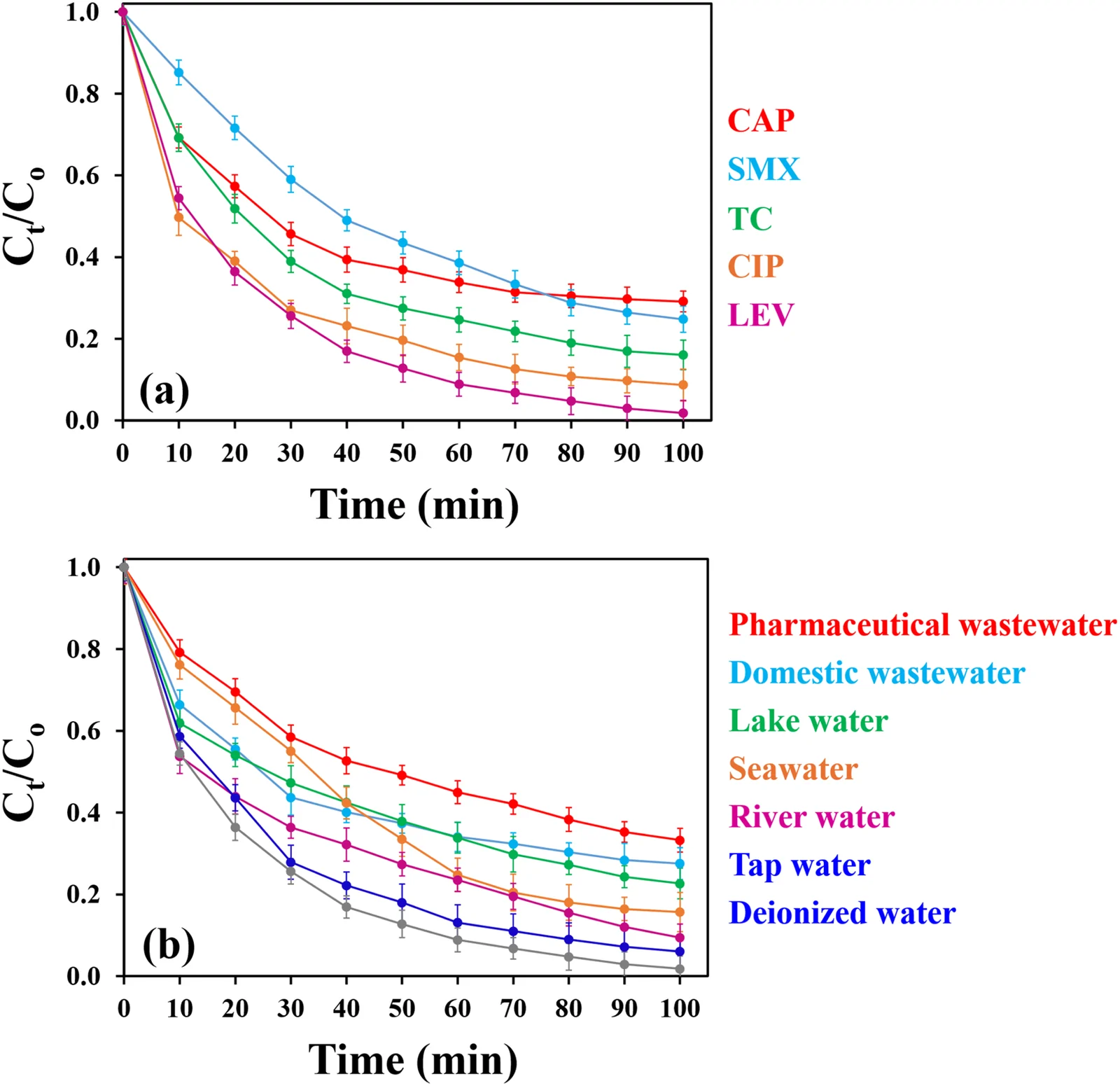
(a) Evaluation of the Ag/Ni-SDBC/PI system for (a) degradation of various antibiotics and (b) LEV degradation in various aqueous matrices. Conditions: [pollutant]_o_ = 10 mg L^−1^, [PI]_o_ = 2 mM, [Ag/Ni-SDBC]_o_ = 0.5 g L^−1^, pH = 7, *T* = 25 °C, and reaction time = 100 min.

### 3.10. Treatment performance of the proposed system in various water matrices

To evaluate the practical applicability of the Ag/Ni-SDBC/PI system under real environmental conditions, degradation experiments were conducted using various water matrices (tap water, river water, lake water, seawater, domestic wastewater, and untreated pharmaceutical industrial wastewater). Each water matrix was spiked with LEV to an initial concentration of 10 mg L^−1^ prior to treatment. The physicochemical characteristics of the investigated water matrices are summarized in Tables S12. The experiments were performed under the optimum operating conditions ([PI]_o_ = 2 mM, [catalyst]_o_ = 0.5 g L^−1^, and neutral pH) at room temperature for a reaction time of 100 min. As shown in Fig. 14(b) and Table S13, LEV removal efficiency decreased progressively from 98.2% in deionized water to 93.95%, 90.56%, 84.31%, 77.35%, 72.46%, and 66.76% in tap water, river water, seawater, lake water, domestic wastewater, and pharmaceutical industrial wastewater, respectively. A corresponding decline was observed in the *k*_1_ values, which decreased from 0.04 min^−1^ in deionized water to 0.031, 0.0244, 0.0207, 0.0169, 0.0158, and 0.0124 min^−1^ in the respective water matrices. The mineralization performance exhibited a similar trend, with TOC removal efficiencies decreasing from 76.33% in deionized water to 70.79%, 64.83%, 56.92%, 49.18%, 40.26%, and 32.48%, respectively. The results indicate that the effectiveness of the Ag/Ni-SDBC/PI system declined with increasing water matrix complexity. Accordingly, the highest LEV degradation and mineralization efficiencies were obtained in deionized water and tap water, whereas lower removal efficiencies were recorded in river water, lake water, seawater, domestic wastewater, and pharmaceutical industrial wastewater due to the greater complexity of these matrices (Tables S12). High TOC and COD levels observed in these matrices indicate an increased organic load, which can consume reactive species and compete with LEV for catalytic active sites, consequently lowering the fraction of oxidizing species available for target pollutant degradation.^43^ In addition, the increased concentrations of dissolved inorganic ions, as reflected by high TDS levels, may scavenge reactive radicals and convert them into less reactive species, thereby reducing the overall oxidation efficiency.^53^ These inhibitory effects were particularly pronounced in pharmaceutical industrial wastewater and domestic wastewater, which exhibited the highest matrix complexity among the investigated water matrices (Tables S12) and consequently showed the lowest LEV degradation and TOC removal efficiencies. A noticeable reduction in treatment performance was also observed in seawater, which can be largely attributed to its elevated TDS value. Despite the inhibitory effects of complex water constituents, the Ag/Ni-SDBC/PI system maintained appreciable degradation efficiencies in all tested matrices, underscoring its robustness and its potential for practical implementation in real wastewater treatment.

### 3.11. Evaluation of the economic feasibility of the degradation process

Most reports depend only on the removal efficiency attained by the degradation system as the main indicator for judging the applicability of the proposed degradation system, while ignoring the economic aspects. Decision makers always need feasibility study prior to the acceptance of new technologies for the real-world application. Thus, economic analysis was performed to appraise the financial performance of the degradation system. The techno-economic analysis represents a preliminary evaluation of the degradation system's profitability rather than evidence of its large-scale feasibility. The large-scale feasibility can be evaluated *via* the fabrication of a large-scale reactor for the degradation of real complex wastewater.

The total cost was estimated to include capital cost and operating cost based on the 2026 prices in Egypt obtained from national and international suppliers. The details of feasibility study are outlined in Table S14. Capital cost comprises reactor cost, equipment cost, and other costs. Reactor cost encompasses the expenses for construction, design, contractor, electrical works, and plumbing works. The reactor's capacity is 10 m^3^, which can operate four cycles per day. The time of one cycle is 2 h, and the total working hours per day (*t*_w_) is 8 h, while the number of working days per year (D) is 300 days. Thus, the yearly treated capacity of wastewater is 12 000 m^3^. Further, equipment cost comprises pumps, magnetic stirrers, oven, and muffle furnace. Three pumps and magnetic stirrers can be used during the reaction, and two pumps and magnetic stirrers can be kept as standby. Pumps will be utilized for controlling the flow rate and direction, whereas magnetic stirrers can be utilized for mixing solutions. Oven and muffle furnace will be employed for the preparation of the metal-loaded biochar. Regarding other costs, this item can cover variations in price, equipment outage, and other contingencies. The capital cost is 0.33 USD per m^3^.

For operating cost, it consists of chemicals, tax, labor, maintenance, and energy consumption. Chemicals cost covers the expenses for purchasing PI and metal salts *via* multiplying the chemical dose (kg m^−3^) by the unit price (USD per kg). The doses were estimated from the experimental work, and unit prices were determined by consulting suppliers in Egypt. Labor, taxes, and maintenance costs are measured as a fraction of the yearly capital cost as listed in Table S14. The unit operating cost is nearly 1 USD per m^3^, and the total cost is 1.33 USD per m^3^.

On the other hand, revenues can be realized by the proposed degradation system through TOC mitigation, reclaimed water reuse, and biochar commercialization. The shadow price of eliminating TOC is about 0.15 USD per Kg_TOC removed_, and the one cubic meter price of reclaimed water is 0.5 USD. Also, dissemination activities can be attained for marketing the modified biochar to industrial facilities for effectively treating their effluents before the release to aquatic bodies, thereby sticking to the environmental regulations. The expected yearly production of the modified biochar is 5000 kg with a unit price of 2.5 USD.

The net profit can be then calculated by subtracting total profits from operating cost. The payback period (PBP), net present value (NPV), internal rate of return (IRR), and profitability index were quantified to examine the economic performance and possible investment in the proposed system. The PBP is lower than the project's lifespan, which contributes to compensating capital cost and earning profits. Further, NPV is positive, and IRR is higher than the interest rate (6%) verifying that the investment by stakeholders in the proposed project is more effective than keeping their money in banks and gaining interests. Moreover, profitability index is higher than 1, ratifying the economic profitability of the proposed degradation system. A sensitivity analysis was also performed to examine the NPV under the variations of key parameters by ±20% such as capital cost, chemical cost, modified biochar price, and reclaimed water price (Fig. 15). The NPV remains positive, which confirms the degradation system's resilience towards prices' variation. In the future, the prepared catalyst can be employed in energy applications by fabricating a composite with a photocatalyst for hydrogen production and utilization as a conductive electrode in zinc-ion batteries.^88–90^ Further, the feasibility of these applications has to be evaluated to determine the most suitable catalyst application.

**Fig. 15:**
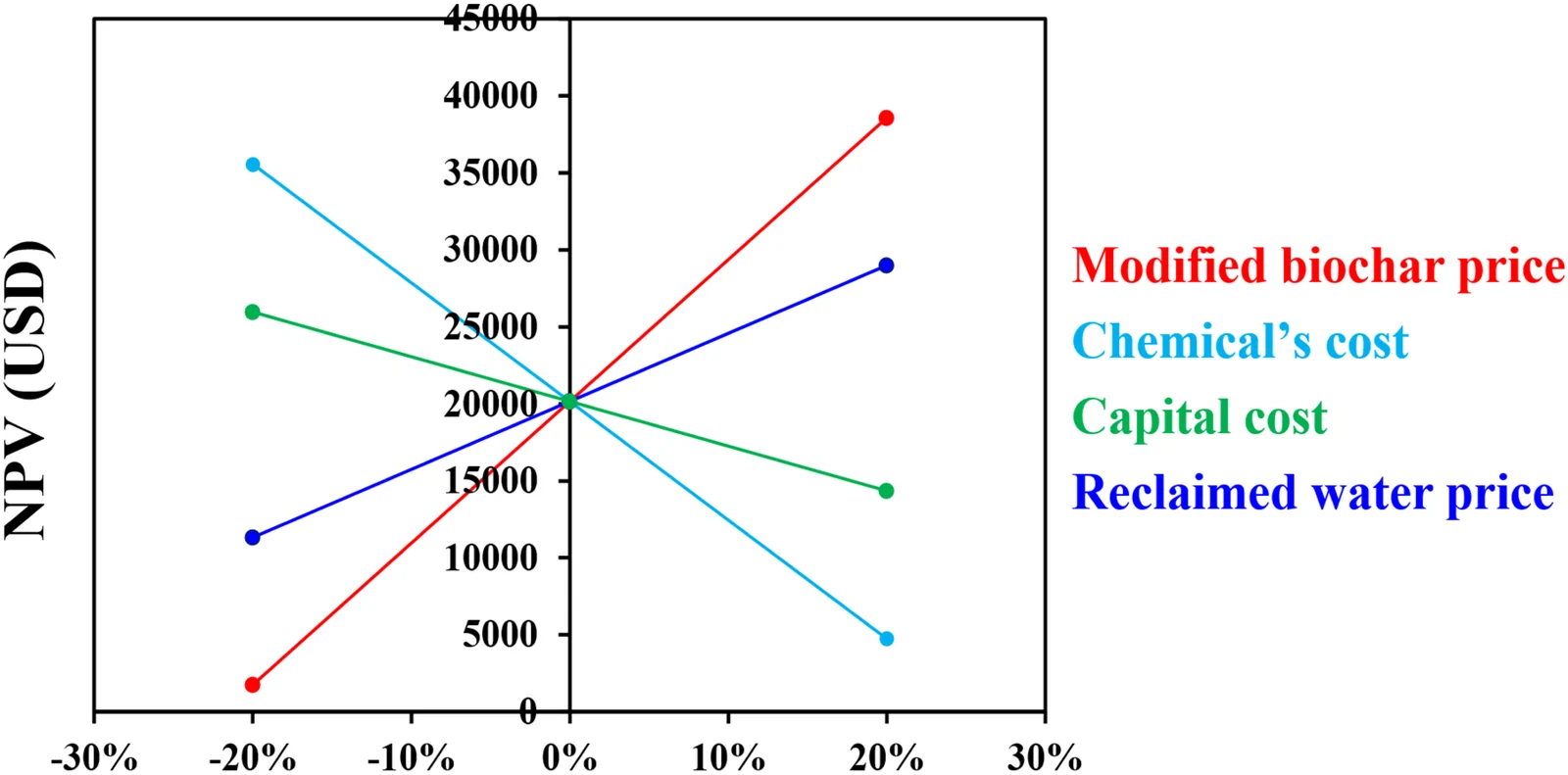
NPV response with the variations of chemicals' cost, modified biochar price, reclaimed water price, and capital cost.

## 4. Conclusions

The SDBC was successfully loaded with Ag and Ni to produce a bimetallic catalyst for PI activation to degrade the fluoroquinolone antibiotic LEV. Comprehensive characterization confirmed the successful loading of Ag and Ni on the SDBC's surface and the abundance of surface oxygen-containing functional groups. Compared with pristine SDBC and the corresponding monometallic-loaded SDBC, the bimetallic-modified SDBC exhibited superior catalytic performance. Under the optimum operating conditions (PI concentration = 2 mM, pH 7, catalyst dosage = 0.5 g^−1^, and initial LEV concentration = 10 mg L^−1^), the Ag/Ni-SDBC/PI system achieved 98.2% LEV degradation. The degradation efficiency of LEV decreased from 98.2% to 89.57% within five consecutive cycles, evidencing the recyclability of the catalyst. Mechanistic investigations revealed that LEV degradation proceeded through the generation of multiple reactive species, with singlet oxygen serving as the dominant oxidizing species. The developed system maintained high degradation efficiency in the presence of Cl^−^, NO_3_^−^, sulfate SO_4_^2−^, whereas HCO_3_^−^ exhibited a pronounced inhibitory effect. Furthermore, TOC mineralization efficiencies of 49.26% and 32.48% were achieved in real domestic and pharmaceutical wastewater, respectively. The absence of toxic iodinated by-products, together with the catalyst's reusability, high degradation efficiency, robustness in real wastewater and different water matrices, and favorable economic performance demonstrate the potential of the proposed Ag/Ni-SDBC/PI system for wastewater treatment applications. Future work will focus on conducting a life cycle assessment to comprehensively evaluate the environmental sustainability of the proposed process. The degradation intermediates can be identified, and their toxicity will be systematically assessed to confirm the environmental safety of the treated effluent. Further, X-ray photoelectron spectroscopy (XPS) analysis can be performed to identify the oxidation states and electronic characteristics of the loaded metals to deeply understand the electron-transfer mechanism, while electron paramagnetic resonance (EPR) can be employed to detect the generated reactive species. In addition, a continuous-flow reactor can be designed and implemented for integration into industrial wastewater treatment facilities to treat industrial effluent prior to its discharge to wastewater treatment plants or receiving water bodies.

## Author contributions

Abdullah S. Bostaji: formal analysis, investigation, methodology, visualization, and writing – original draft. Nasr Rashid: formal analysis, funding acquisition, investigation, project administration, resources, software, validation, and writing – review & editing. Bandar Alwushayh: investigation, methodology, validation, visualization, and writing – review & editing. Abdelhalim Azam: investigation, methodology, visualization, and writing – review & editing. Karim Amer: conceptualization, investigation, methodology, data curation, supervision, visualization, validation, writing – original draft, and writing – review & editing.

## Conflicts of interest

There are no conflicts to declare.

## Supplementary Material

RA-016-D6RA06933A-s001

## Data Availability

Data will be made available on request. Supplementary information (SI) is available. See DOI: https://doi.org/10.1039/d6ra06933a.
