# Supplementary material for: Novel Ag/Ni bimetallic-loaded sawdust-derived biochar as a periodate activator for the effective degradation of levofloxacin
Source: RSC Adv. 2026 Sep 14;16(44):55067–93. doi: 10.1039/d6ra06933a (PMC13573864; doi:10.1039/d6ra06933a)
Supplement: Supplementary file 1 [file RA-016-D6RA06933A-s001.pdf]

Supplementary Information (SI) for RSC Advances.
This journal is © The Royal Society of Chemistry 2026

Supplementary Information

Novel Ag/Ni bimetallic-loaded sawdust-derived biochar as a periodate activator for the effective degradation of levofloxacin

Abdullah S. Bostaji [1], Nasr Rashid [b, *], Bandar Alwushayh [c], Abdelhalim Azam [c], Karim Amer [d, *]

[a] Department of Civil Engineering, College of Engineering and Architecture, Umm Al-Qura University, Makkah 24381, Saudi Arabia; asbostaji@uqu.edu.sa

[b] Department of Electrical Engineering, College of Engineering, Jouf University, Sakaka 72388, Saudi Arabia; nasrrashid@ju.edu.sa

[c] Department of Civil Engineering, College of Engineering, Jouf University, Sakaka 72388, Saudi Arabia; baalwushayh@ju.edu.sa (B.A.); amazam@ju.edu.sa (A.A.)

[d] Technology Management Department, Egypt-Japan University of Science and Technology (E-JUST), P.O. Box 179, New Borg El-Arab City Postal Code 21934, Alexandria, Egypt; karim.amer@ejust.edu.eg

Corresponding authors:

* nasrrashid@ju.edu.sa (Nasr Rashid); karim.amer@ejust.edu.eg (Karim Amer).

Supplementary texts

Text S1. Chemicals

Levofloxacin (LEV, $C_{18}H_{20}FN_3O_4$, analytical standard), chloramphenicol (CAP, $C_{11}H_{12}Cl_2N_2O_5$, analytical standard), sulfamethoxazole (SMX, $C_{10}H_{11}N_3O_3S$, analytical standard), tetracycline (TC, $C_{22}H_{24}N_2O_8$, analytical standard), ciprofloxacin (CIP, C17H18FN3O$_3$, analytical standard), potassium periodate (KIO_4, 99.8%), p-benzoquinone (p-BQ, $C_6H_4O_2$, 97%), potassium iodide (KI, 99%), L-histidine (L-His, $C_6H_9N_3O_2$, ≥99.5%), ammonium molybdate ($(NH_4)_6Mo_7O_{24}$, ≥99%), and sodium iodate ($NaIO_3$, ≥ 99%) were supplied by Sigma-Aldrich (St. Louis, MO, USA). In addition, hydrogen peroxide (H_2O_2, 30%), ethanol (C_2H_6O, 99.8%), sodium hydroxide (NaOH, >97%), potassium chloride (KCl, 99.5%), potassium nitrate (KNO_3, 99%), sodium bicarbonate ($NaHCO_3$, 99.5%), acetonitrile (C_2H_3N, HPLC grade), phosphoric acid (H_3PO_4, HPLC grade), humic acid (HA, technical grade, average molecular weight = 3000, 95%), furfuryl alcohol (FFA, $C_5H_2O_2$, 98%), acetic acid glacial (CH_3COOH, HPLC grade), phenol (C_6H_5OH, ACS reagent grade, ≥98%), oxalic acid ($H_2C_2O_4$, ≥99.5%), amylopectin ($C_{30}H_{52}O_{26}$, 99.8%), methanol (CH_4O, HPLC grade), silver nitrate ($AgNO_3$, ≥99.9%), sodium sulfate (Na_2SO_4, 99%), and tert-butyl alcohol (TBA, $C_4H_{10}O$, 99.5%) were obtained from Fischer Scientific (Loughborough, UK). Moreover, hydrochloric acid (HCl, 37%), potassium persulfate ($K_2S_2O_8$, 99.5%), potassium peroxymonosulfate ($KHSO_5$, 99.9%), nickel (II) chloride hexahydrate ($NiCl_2 \cdot 6H_2O$, 99.9%), and sodium thiosulfate ($Na_2O_3S_2$, ≥97%) were purchased from Merck KGaA (Darmstadt, Germany).

Text S2. Characterization equipment and categorization

X-ray diffraction (XRD, XRD 6000, Shimadzu Corp., Tokyo, Japan) was utilized to ascertain the crystalline phases of the synthesized materials. The Fourier-transform infrared spectrum (FT-IR) was employed to detect the functional groups on the surface of the catalysts (FTIR-8400S,

Shimadzu Corp., Tokyo, Japan). The dimensions, microstructure, morphology, and elemental composition of the catalysts were examined by scanning electron microscopy (SEM, JSM-6010L, JEOL Ltd., Tokyo, Japan) and transmission electron microscopy (TEM, JEM 2100F, JEOL Ltd., Tokyo, Japan) equipped with energy-dispersive X-ray spectroscopy (EDX, 51XMX 1004, Oxford Instruments, UK). The specific surface area (SA) and pore properties of the catalysts were explored using a surface area analyzer (BELSORP-max, BEL Japan Inc., Osaka, Japan) by adopting the Brunauer–Emmett–Teller (BET) method and the Barrett–Joyner–Halenda (BJH) model, respectively. The point of zero charge (pH_{PZC}) of the synthesized materials was determined using the pH drift method to evaluate their surface charge characteristics and identify the pH at which the net surface charge is zero, thereby providing insight into pH-dependent electrostatic interactions during the adsorption process. A 0.01 M KCl solution was used as the background electrolyte to maintain a constant ionic strength. Briefly, 0.01 g of the material was added to 50 mL of KCl solution with initial pH values adjusted between 2 and 12. The resulting suspensions were stirred at 220 rpm for 48 h at 25 °C to allow equilibrium to be established between the solid and liquid phases. Subsequently, the final pH of each suspension was measured, and the pH_{PZC} value was determined by plotting the difference between the initial and final pH values (ΔpH) versus the initial pH. The pH corresponding to $\Delta pH = 0$ was taken as the pH_{PZC} value [1,2].

Text S3. Detailed analytical methods

The concentrations of LEV, CAP, SMX, TC, CIP, periodate (PI, IO_4^-), iodate ions (IO_3^-) ions, and iodide ions (I^-) were determined using a high-performance liquid chromatography (HPLC) system (LC–2040C, Shimadzu Corp., Tokyo, Japan) equipped with a diode array detector (DAD) and an automated sample injector. Chromatographic separation was achieved on an Agilent ZORBAX Eclipse XDB-C18 column (4.6 × 250 mm, 5 μm; Agilent Technologies, Santa Clara,

CA, USA). Prior to analysis, all samples were filtered through a 0.22 μm membrane to remove suspended catalyst particles. LEV concentrations were quantified according to the method of Alvarez-Serna et al. using a mobile phase of 0.1% (v/v) phosphoric acid and acetonitrile (85:15, v/v) at a flow rate of 1 mL min^{-1}, an injection volume of 20 μL, ambient column temperature, and detection at 295 nm [3]. CAP was analyzed following Saleh et al. using a mobile phase comprising deionized water (adjusted to pH 3.5 with phosphoric acid), acetonitrile, and methanol (60:19:21, v/v/v) at a flow rate of 1.57 mL min^{-1}, a 20 μL injection volume, and a detection wavelength of 285 nm [4]. SMX was determined according to Wang et al. using 0.1% (v/v) acetic acid in deionized water and acetonitrile (75:25, v/v) as the mobile phase at a flow rate of 1 mL min^{-1}, with a 20 μL injection volume, a column temperature of 30 °C, and detection at 270 nm [5]. TC concentrations were measured following Butovskaya et al. using 0.01 mol L^{-1} oxalic acid and acetonitrile under gradient elution at a flow rate of 1 mL min^{-1}, with a 10 μL injection volume and detection at 360 nm [6]. CIP was quantified according to Zhang et al. using 0.1% (v/v) phosphoric acid and acetonitrile (87:13, v/v) at a flow rate of 1 mL min^{-1}, with a 20 μL injection volume, a column temperature of 30 °C, and detection at 278 nm [7]. The concentrations of IO_4^-, IO_3^-, and I^- ions were determined by the external standard calibration method using KIO_4, $NaIO_3$, and KI standard solutions, respectively, over a concentration range of 0–500 mM [8,9]. Their chromatographic analysis followed the method of Xiong et al. using a mobile phase of 0.1% (v/v) phosphoric acid and acetonitrile (70:30, v/v) at a flow rate of 1 mL min^{-1}, a 10 μL injection volume, a column temperature of 30 °C, and detection at 228 nm [10]. Quantification of all analytes was performed using external calibration curves prepared from corresponding standard solutions and analyzed under their respective chromatographic conditions.

Hypoiodous acid (HOI) was detected indirectly by monitoring its reaction with phenol. In this analysis, a 0.5 mM phenol solution was used as the reaction medium instead of LEV. HOI readily iodinates phenol to form 2-iodophenol (2-IP) and 4-iodophenol (4-IP), which were identified by HPLC according to the method reported by Bichsel et al. [11]. Chromatographic separation was performed using a water/acetonitrile (60:40, v/v) mobile phase, and the products were detected at 286 nm. Qualitative identification of HOI was confirmed by comparing the retention times of the reaction products with those of authentic 2-IP and 4-IP standards.

The formation of molecular iodine (I_2), and triiodide ions (I_3^-) was evaluated using the starch colorimetric method, based on the equilibrium between I_3^- and its dissociation products, as described by Eq. (S1) [12]. A saturated starch stock solution was prepared by dispersing 1 g of branched starch (amylopectin) in 10 mL of deionized water, followed by gradual addition to 90 mL of boiling deionized water. The mixture was heated with continuous stirring and boiled for an additional 5 min, then cooled to room temperature (T ~ 25 °C) before use [13]. At predetermined reaction intervals, 0.5 mL aliquots were withdrawn, filtered through a 0.22 μm membrane, and transferred to clear vials. The starch stock solution was subsequently added to achieve a final starch concentration of 1 g L^{-1}, and the mixtures were thoroughly homogenized. The appearance of a blue-purple coloration, resulting from the starch–iodine complex, qualitatively confirmed the presence of I_2 and I_3^- ions. The color development was recorded after 12 h of incubation at room temperature [14].

$$I_3^- \leftrightarrow I_2 + I^- \quad \text{(S1)}$$

The residual persulfate (PS) concentrations during the oxidation process in the PS-based system was determined using a KI-based colorimetric method [15,16]. The color-developing reagent was prepared by dissolving 2 g of KI and 0.1 g of $NaHCO_3$ in 20 mL of deionized water. For each

analysis, a 0.1 mL aliquot of the filtered reaction sample was added to the reagent solution, mixed thoroughly, and allowed to react for 15 min at room temperature. In the presence of $NaHCO_3$, the reaction of PS with KI generated a yellow-colored complex. The intensity of the developed color, which is directly proportional to the residual PS concentration, was quantified by measuring the absorbance at 352 nm using a UV–Vis spectrophotometer (Evolution 350, Thermo Fisher Scientific, Waltham, USA).

In the peroxymonosulfate (PMS)-based system, residual PMS concentrations were quantified using the iodometric spectrophotometric method. During the reaction, aliquots were collected at predetermined time intervals, immediately filtered through a 0.22 μm membrane to remove suspended catalyst particles, and subsequently treated with an excess of KI (1 mol L^{-1}) in the presence of 0.1mol L^{-1} HCl to establish acidic conditions. Under these conditions, PMS quantitatively oxidized iodide ions to iodine, which readily reacted with excess iodide to form I_3^- ions. The resulting solution was allowed to stand in the dark for approximately 10 min to ensure complete color development before measuring the absorbance of I_3^- ions at 352 nm using a UV–Vis spectrophotometer. Residual PMS concentrations were subsequently determined from a calibration curve constructed using PMS standard solutions analyzed following the same procedure [17].

Residual H_2O_2 concentrations were determined using the N,N-diethyl-p-phenylenediamine (DPD) colorimetric method (Hach Method 10290), which provides a quantification range of 0.05–5 mg L^{-1} H_2O_2. The method is based on the oxidation of the DPD reagent by H_2O_2 in the presence of KI and ammonium molybdate, resulting in the formation of a stable, pink-colored product whose absorbance is directly proportional to the H_2O_2 concentration. For the analysis, 10 mL of the sample was transferred into a clean sample cell, followed by the addition of three drops of 20%

KI solution and three drops of ammonium molybdate reagent. The mixture was gently inverted to ensure complete mixing and allowed to react for 6 min. Subsequently, one DPD powder pillow (designed for 25 mL samples) was added, and the solution was shaken for approximately 20 s to facilitate complete dissolution. After a color development period of 30 s, the absorbance was measured at 530 nm using a portable spectrophotometer (DR1900, HACH Company, Loveland, USA), with a reagent blank prepared under identical conditions serving as the reference. The concentration of residual H_2O_2 was calculated from the measured absorbance and expressed in mg L^{-1}. To minimize H_2O_2 decomposition, all samples were analyzed immediately after collection without prior preservation. The DPD colorimetric method offers high sensitivity and selectivity for H_2O_2 determination in aqueous media while exhibiting negligible interference under near-neutral conditions (pH 6–7).

Total organic carbon (TOC) was quantified using the high-temperature combustion method according to the Standard Methods for the Examination of Water and Wastewater (SMWW) (APHA, 2017), specifically Method 5310B, with a TOC-L CPH/CPN total organic carbon analyzer (Shimadzu, Tokyo, Japan). Chemical oxygen demand (COD) was analyzed using the closed reflux colorimetric method (SMWW 5220D), in which samples were first digested in a thermal digester (RD 125, Lovibond, Dortmund, Germany) and subsequently analyzed with a portable spectrophotometer. The pH values were determined in accordance with the SMWW 4500-H^+B method using a PHC301 pH electrode connected to an HQ440d multiparameter meter (HACH Company, Loveland, USA). Total dissolved solids (TDS) and total suspended solids (TSS) were measured gravimetrically following the SMWW 2540C and SMWW 2540D methods by drying the samples at 180 °C and 103–105 °C, respectively. Turbidity was measured following the

nephelometric method (SMWW 2130B), employing a Turb 430T turbidimeter (WTW, Weilheim, Germany).

Supplementary tables

Table S1 Collection sources of water matrices used in the present study.

Water matrix	Source
Pharmaceutical industrial wastewater	Pharmaceutical facility (New Borg El-Arab City, Alexandria, Egypt)
Domestic wastewater	Domestic wastewater treatment plant (New Borg El-Arab City, Alexandria, Egypt)
Lake water	Lake Mariout (Alexandria, Egypt)
Seawater	Mediterranean Sea (Alexandria, Egypt)
River water	Nile River (Cairo, Egypt)
Tap water	Alexandria, Egypt
Deionized water	Lab water purifier (WG251, Yamato Scientific Co., Ltd., Tokyo, Japan)

Table S2 Summary of the equations used for performance evaluation and kinetic analysis.

Equation	No.
$\textbf{Pollutant RE}\ (\%) = \frac{[\textbf{pollutant}]_{\textbf{o}} - [\textbf{pollutant}]_{\textbf{t}}}{[\textbf{pollutant}]_{\textbf{o}}}\ \textbf{x 100}$	(1)
$\textbf{Oxidant utilization rate}\ (\%) = \frac{[\textbf{oxidant}]_{\textbf{o}} - [\textbf{oxidant}]_{\textbf{t}}}{[\textbf{oxidant}]_{\textbf{o}}}\ \textbf{x 100}$	(2)
$\textbf{TOC RE}\ (\%) = \frac{[\textbf{TOC}]_{\textbf{o}} - [\textbf{TOC}]_{\textbf{t}}}{[\textbf{TOC}]_{\textbf{o}}}\ \textbf{x 100}$	(3)
$\textbf{Q} = \frac{[\textbf{pollutant}]_{\textbf{o}} - [\textbf{pollutant}]_{\textbf{t}}}{\textbf{m}}\ \textbf{x V}$	(4)
$\mathbf{C_t = C_o - k_o \times t}$	(5)
$\mathbf{ln\left(\frac{C_o}{C_t}\right) = k_1 \times t}$	(6)
$\mathbf{\frac{1}{C_t} = \frac{1}{C_o} + k_2 \times t}$	(7)
$\mathbf{SI = \frac{k_{(catalyst + PI)}}{k_{(catalyst)} + k_{(PI)}}}$	(8)

RE: removal efficiency (%), t: reaction time (min), $[\text{pollutant}]_o$: initial pollutant concentration (mg L^{-1}), $[\text{pollutant}]_t$: pollutant concentration at reaction time t (mg L^{-1}), $[\text{oxidant}]_o$: initial oxidant concentration (mM), $[\text{oxidant}]_t$: oxidant concentration at reaction time t (mM), TOC_o: initial TOC concentration (mg L^{-1}), TOC_t: TOC at reaction time t (mg L^{-1}), Q: adsorption capacity (mg g^{-1}),V: volume of the solution (L), m: mass of the adsorbent (g), k_o: zero-order rate constant (mg L^{-1} min^{-1}), k_1: pseudo-first-order rate constant (min^{-1}), k_2: pseudo-second-order rate constant (L mg^{-1} min^{-1}), SI: synergy index, $k_{(catalyst + PI)}$: pseudo-first-order rate constant of the catalyst/PI system (min^{-1}), $k_{(catalyst)}$: pseudo-first-order rate constant of the catalyst-alone (adsorption) system (min^{-1}), $k_{(PI)}$: pseudo-first-order rate constant of the PI-alone (adsorption) system (min^{-1}).

Table S3 LEV removal efficiency, first-order kinetic rate constants, adsorption capacities, and synergistic factors across various treatment systems.

System	LEV RE (%)	k_1 (min^{-1})	Coefficient of determination (R^2)	Q ($mg\ g^{-1}$)	SI
[a] SDBC alone	15.23	0.0019	0.9649	7.62	–
[b] Ag-SDBC alone	19.13	0.0026	0.9548	9.57	–
[c] Ni-SDBC alone	22.8	0.0032	0.9376	11.4	–
[d] Ag/Ni-SDBC alone	28.35	0.004	0.9558	14.18	–
PI alone	11.69	0.0015	0.9549	–	–
SDBC/PI	52.95	0.0089	0.9628	–	2.62
Ag-SDBC/PI	72.63	0.0155	0.9552	–	3.78
Ni-SDBC/PI	85.2	0.0225	0.9577	–	4.79
Ag/Ni-SDBC/PI	98.2	0.04	0.997	–	7.27

[a] SDBC: Sawdust-derived biochar.

[b] Ag-SDBC: Silver-loaded sawdust-derived biochar.

[c] Ni-SDBC: Nickel-loaded sawdust-derived biochar.

[d] Ag/Ni-SDBC: Silver/nickel co-loaded sawdust-derived biochar.

Experimental conditions: $[LEV]_o = 10\ mg\ L^{-1}$, $[PI]_o = 2\ mM$, $[catalyst]_o = 0.5\ g\ L^{-1}$, pH = 7, T = 25 °C, and reaction time = 100 min.

Table S4 Pollutant degradation efficiency, first-order kinetic analysis, and oxidant utilization for LEV degradation in the Ag/Ni-SDBC/PI system using different oxidants.

Oxidant	LEV RE (%)	k_1 (min^{-1})	R^2	Oxidant utilization rate (%)
H_2O_2	56.75	0.0099	0.9581	63.84
PMS	74.38	0.0161	0.9536	76.93
PS	83.03	0.0203	0.9626	82.62
PI	98.2	0.04	0.997	91.35

Experimental conditions: $[LEV]_o$ = 10 mg L^{-1}, $[oxidant]_o$ = 2 mM, $[Ag/Ni\text{-}SDBC]_o$ = 0.5 g L^{-1}, pH = 7, T = 25 °C, and reaction time = 100 min.

Table S5 Influence of operational conditions on the LEV removal efficiency and pseudo-first-order degradation kinetics in the Ag/Ni-SDBC/PI system.

Parameter	Tested value	LEV RE (%)	k_1 (min^{-1})	R^2	Optimum value
pH	3	100 (after 60 min)	0.065	0.9985	7
	5	100 (after 80 min)	0.0509	0.9955	
	7	98.2	0.04	0.997	
	9	82.31	0.0195	0.9932	
Initial PI concentration (mM)	0.5	73.47	0.0145	0.9915	2
	1	81.6	0.0177	0.9922	
	1.5	89.72	0.0233	0.9895	
	2	98.2	0.04	0.997	
	2.5	98.11	0.034	0.9885	
	5	98.1	0.0331	0.9847	
Ag/Ni-SDBC dosage (g L^{-1})	0.1	63.63	0.0099	0.9907	0.5
	0.3	80.31	0.0189	0.9842	
	0.5	98.2	0.04	0.997	
	0.7	86.93	0.0241	0.9731	
	1	81.87	0.0202	0.9728	
Initial LEV concentration (mg L^{-1})	5	100 (after 60 min)	0.0682	0.9767	10
	10	98.2	0.04	0.997	
	15	88.65	0.0251	0.9654	
	20	74.14	0.0169	0.9394	

Experimental conditions: $[LEV]_o$ = 10 mg L^{-1}, $[PI]_o$ = 2 mM, $[Ag/Ni\text{-}SDBC]_o$ = 0.5 g L^{-1}, pH = 7, T = 25 °C, and reaction time = 100 min.

Table S6 Effect of common water constituents on LEV degradation by the Ag/Ni-SDBC/PI system in terms of efficiency and first-order kinetics.

Coexisting constituent	Tested value	LEV RE (%)	k_1 (min^{-1})	R^2
Without any additions	–	98.2	0.04	0.997
Chloride ions (Cl^-) (mM)	1	96.39	0.0339	0.9974
	5	95.58	0.0322	0.9812
	10	95.2	0.0292	0.9964
	20	94.99	0.0282	0.9954
Nitrate ions (NO_3^-) (mM)	1	96.46	0.0302	0.997
	5	95.2	0.0283	0.9892
	10	94.79	0.0278	0.9929
	20	94.4	0.0269	0.9873
Sulfate ions (SO_4^{2-}) (mM)	1	97.01	0.0332	0.9965
	5	96.65	0.0326	0.9968
	10	96.14	0.0315	0.9962
	20	95.71	0.0279	0.99
Bicarbonate ions (HCO_3^-) (mM)	1	90.09	0.0243	0.9858
	5	78.08	0.0162	0.9823
	10	72.16	0.014	0.9824
	20	69.07	0.0129	0.9922
HA (mg L^{-1})	1	95.06	0.0278	0.996
	5	97.82	0.0334	0.9888
	10	98.06	0.0378	0.9901
	20	98.83	0.0377	0.9843

Experimental conditions: $[LEV]_o$ = 10 mg L^{-1}, $[PI]_o$ = 2 mM, $[Ag/Ni\text{-}SDBC]_o$ = 0.5 g L^{-1}, pH = 7, T = 25 °C, and reaction time = 100 min.

Table S7 Summary of the reactions of inorganic anions with reactive species responsible for LEV degradation in the Ag/Ni-SDBC/PI system.

Inorganic anion	Reaction process: Equation	No.	Reference
Cl^-	$Cl^- + IO_3^\bullet \rightarrow IO_3^- + Cl^\bullet$	(1)	19
	$Cl^- + IO_4^- + H_2O \rightarrow IO_3^\bullet + Cl^\bullet + 2OH^-$	(2)	20
	$Cl^- + HO^\bullet \rightarrow ClHO^{\bullet -}$	(3)	14
	$ClHO^{\bullet -} \rightarrow OH^- + Cl^\bullet$	(4)	21
	$ClHO^{\bullet -} + H^+ \rightarrow H_2O + Cl^\bullet$	(5)	22
	$Cl^\bullet + Cl^\bullet \rightarrow Cl_2$	(6)	23
	$Cl^- + Cl^\bullet \rightarrow Cl_2^{\bullet -}$	(7)	14
	$Cl_2^{\bullet -} + Cl_2^{\bullet -} \rightarrow Cl_2 + 2Cl^-$	(8)	23
	$Cl_2 + H_2O \rightarrow HOCl + HCl$	(9)	23
SO_4^{2-}	$SO_4^{2-} + HO^\bullet \rightarrow OH^- + SO_4^{\bullet -}$	(10)	24
NO_3^-	$NO_3^- + IO_3^\bullet \rightarrow IO_3^- + NO_3^\bullet$	(11)	19
	$NO_3^- + HO^\bullet \rightarrow OH^- + NO_3^\bullet$	(12)	21
HCO_3^-	$HCO_3^- + IO_3^\bullet \rightarrow IO_3^- + HCO_3^\bullet$	(13)	19
	$HCO_3^- + IO_3^\bullet \rightarrow IO_3^- + H^+ + CO_3^{\bullet -}$	(14)	20
	$HCO_3^- + HO^\bullet \rightarrow OH^- + HCO_3^\bullet$	(15)	21
	$HCO_3^- + HO^\bullet \rightarrow H_2O + CO_3^{\bullet -}$	(16)	25
	$HCO_3^- + {}^1O_2 \rightarrow OH^- + CO_3^{\bullet -}$	(17)	26
	$CO_3^{\bullet -} + CO_3^{\bullet -} \rightarrow CO_2 + CO_4^{2-}$	(18)	22
	$2CO_3^{\bullet -} + H_2O \rightarrow 2CO_2 + HO_2^- + OH^-$	(19)	23

Table S8 LEV removal efficiency and first-order degradation rates across five consecutive operational cycles in the Ag/Ni-SDBC/PI system.

Run	LEV RE (%)	Efficincy loss (%)	k_1 (min^{-1})	Reduction in k_1 (%)	R^2
1st run	98.2	–	0.04	–	0.997
2nd run	96.63	1.6	0.0358	10.5	0.9934
3rd run	94.71	3.55	0.0322	19.5	0.9885
4th run	92.33	5.98	0.029	27.5	0.9835
5th run	89.57	8.79	0.026	35	0.9791

Experimental conditions: $[LEV]_o$ = 10 mg L^{-1}, $[PI]_o$ = 2 mM, $[Ag/Ni\text{-}SDBC]_o$ = 0.5 g L^{-1}, pH = 7, T = 25 °C, and reaction time = 100 min.

Table S9 Catalyst mass loss and metal leaching behavior of the Ag/Ni-SDBC catalyst over five repeated runs during LEV degradation in the Ag/Ni-SDBC/PI system.

Run	Catalyst dosage (g L^{-1})	Catalyst mass loss (%)	Leached Ag concentration (mg L^{-1})	Leached Ni concentration (mg L^{-1})
1st run	0.5	–	82×10^{-3}	3.86×10^{-5}
2nd run	0.4829	3.42	1.54×10^{-2}	1.25×10^{-4}
3rd run	0.4545	9.1	2.47×10^{-2}	5.33×10^{-3}
4th run	0.4147	17.06	3.66×10^{-2}	8.41×10^{-2}
5th run	0.3735	25.3	5.38×10^{-2}	1.23×10^{-2}

Experimental conditions: $[LEV]_o$ = 10 mg L^{-1}, $[PI]_o$ = 2 mM, pH = 7, T = 25 °C, and reaction time = 100 min.

Table S10 Effects of reactive species scavengers on LEV degradation and reaction kinetics in the Ag/Ni-SDBC/PI system under the optimum conditions.

Scavenger	LEV RE (%)	Efficincy loss (%)	k_1 (min^{-1})	Reduction in k_1 (%)	R^2
No scavenger	98.2	–	0.04	–	0.997
TBA (10 mM)	68.36	30.39	0.0137	65.75	0.9723
TBA (100 mM)	62.63	36.22	0.0115	71.25	0.977
Phenol (1 mM)	34.51	64.86	0.0048	88	0.9846
Phenol (5 mM)	20.56	79.06	0.0028	93	0.9525
Phenol (10 mM)	15.79	83.92	0.002	95	0.9604
p-BQ (0.02 mM)	89.85	8.5	0.0262	34.5	0.9813
p-BQ (0.05 mM)	81.08	17.43	0.0196	51	0.9761
p-BQ (0.1 mM)	70.98	27.72	0.0149	62.75	0.965
FFA (0.5 mM)	45.37	53.8	0.0071	82.25	0.9782
FFA (5 mM)	5.94	93.95	0.0008	98	0.9279
L-His (0.5 mM)	64.28	34.54	0.0115	71.25	0.9864
L-His (5 mM)	14.92	84.81	0.0019	95.25	0.9755

Experimental conditions: $[LEV]_o$ = 10 mg L^{-1}, $[PI]_o$ = 2 mM, $[Ag/Ni\text{-}SDBC]_o$ = 0.5 g L^{-1}, pH = 7, T = 25 °C, and reaction time = 100 min.

Table S11 Comparison of pollutant degradation efficiency, first-order kinetic rate constants, and mineralization efficiency for different antibiotics in the Ag/Ni-SDBC/PI system.

Pollutant	Pollutant RE (%)	k_1 (min^{-1})	R^2	Initial TOC ($mg\ L^{-1}$)	TOC RE (%)
CAP	70.88	0.0156	0.9472	4.16	41.28
SMX	75.23	0.0153	0.9952	4.65	48.43
TC	83.96	0.0214	0.975	5.87	53.85
CIP	91.29	0.0284	0.9713	6.24	64.57
LEV	98.2	0.04	0.997	5.73	76.33

Experimental conditions: $[Pollutant]_o$ = 10 mg L^{-1}, $[PI]_o$ = 2 mM, $[Ag/Ni\text{-}SDBC]_o$ = 0.5 g L^{-1}, pH = 7, T = 25 °C, and reaction time = 100 min.

Table S12 Physicochemical characteristics of different water matrices used for evaluating LEV degradation performance in the Ag/Ni-SDBC/PI system.

Water matrix	TOC (mg L^{-1})	COD (mg L^{-1})	pH	TDS (mg L^{-1})	TSS (mg L^{-1})	Turbidity (ntu)
Pharmaceutical wastewater	163.24	468.57	7.36	1182.6	236.84	50.28
Domestic wastewater	78.45	215.06	7.81	524.39	148.56	34.74
Lake water	13.72	36.76	8.35	182.24	21.37	6.81
Seawater	10.53	28.89	7.54	35288.75	18.62	5.26
River water	8.42	23.53	8	113.17	12.45	3.57
Tap water	3.68	9.54	7.72	62.36	1.24	0.84
Deionized water	0.05	0.12	6.95	1.87	[a] ND	0.02

[a] ND = Not detected.

Table S13 Effect of water matrices on LEV degradation ratios, pseudo-first-order rate constants, and TOC mineralization in the Ag/Ni-SDBC/PI system.

Water matrix	LEV RE (%)	k_1 (min^{-1})	R^2	Initial TOC concentration ($mg\ L^{-1}$)		TOC removal ratio (%)
				Before LEV addition	After LEV addition	
Pharmaceutical wastewater	66.75	0.0124	0.9817	163.24	168.34	32.48
Domestic wastewater	72.46	0.0158	0.9493	78.45	84.64	40.26
Lake water	77.35	0.0169	0.9699	13.72	19.51	49.18
Seawater	84.31	0.0207	0.9937	10.53	16.42	56.92
River water	90.56	0.0244	0.9834	8.42	14.22	64.83
Tap water	93.95	0.031	0.9877	3.68	8.32	70.79
Deionized water	98.2	0.04	0.997	0.05	5.84	76.33

Experimental conditions: $[LEV]_o = 10\ mg\ L^{-1}$, $[PI]_o = 2$ mM, $[Ag/Ni\text{-}SDBC]_o = 0.5\ g\ L^{-1}$, pH = 7, T = 25 °C, and reaction time = 100 min.

Table S14 Cost analysis details of the proposed degradation system.

Item	Unit	Cost	Justification
		Capital cost	
Reactor cost (X)	USD	16000	Cost of reinforced concrete = 600 USD/m^3, contractor cost = 2000 USD, design cost = 2000 USD, electrical works cost = 3000 USD, and plumbing works = 3000 USD
Equipment (Y)	USD	8000	Pump price = 400 USD, magnetic stirrer price = 200 USD, oven price = 2000 USD, and muffle furnace price = 3000 USD
Other costs (Z)	USD	5000	To account for contingencies such as the downtime of equipment
Total capital cost	USD	29000	The sum of X, Y, and Z
Yearly capital cost	USD/year	3918.92	Equals to total capital cost/($[1-(1+r)^{-t}]$/r), where r is the interest rate (6%), and t refers to the project's lifetime
Unit capital cost	USD/m^3	0.33	Yearly capital cost/yearly treated volume of wastewater
		Operating cost	
Chemical costs (A)	USD/year	10464	KIO_4 dose and unit price = 0.5 kg/m^3 and 1.0 USD/kg, $AgNO_3$ dose and unit price = 0.042 Kg/m^3, 6 USD/kg, and $NiCl_2.6H_2O$ dose and unite price = 0.06 Kg/m^3, 2 USD/kg
Energy cost (B)	USD/year	240	The average power for equipment (muffle furnace, oven, mixers, and pumps) = 500 W, EC = $E \times t_w \times D \times P_E$, where EC is the energy consumption cost in USD/year, E refers to the required power in kW, and PE is the energy unit price (0.2 USD/kW.h))
Labor cost (C)	USD/year	391.9	10% of yearly capital cost
Taxes (D)	USD/year	39.2	1% of yearly capital cost
Maintenance cost (E)	USD/year	783.8	20% of yearly capital cost
Total operating cost	USD/year	11919	Sum of A, B, C, D, and E
Unit operating cost	USD/m^3	1	Total operating cost/treated volume of wastewater per year

Revenues			
TOC abatement	USD/year	98.4	Mass of TOC removed per year (656.12 Kg/year) x shadow price (0.15 USD/$Kg_{TOC\ removed}$)
Reclaimed water	USD/year	6000	Price of reclaimed water per m^3 = 0.5 USD, revenues = 0.5 × 12000 = 6000 USD
Modified biochar price	USD/year	12500	Price of the biochar per kg = 2.5 USD/kg, the yearly production of the material = 5000 kg
Total profits	USD/year	18598.4	Summation of revenues
Feasibility analysis			
Net profit	USD/year	6679.4	Total profits – Total operating cost
Payback period	Years	4.3	Capital cost/Net profit
Net present value (NPV)	USD	20161	$NPV = \sum_{y=1}^{n} \frac{\text{Net profit}}{(1+r)^t} - \text{capital cost}$
Internal rate of return (IRR)	%	20.46	$\text{Capital cost} = \sum_{t=1}^{n} \frac{\text{Net profit}}{(1+IRR)^t}$
Profitability index	–	1.7	$\text{Profitability index} = \frac{\text{NPV+Capital cost}}{\text{Capital cost}}$

Supplementary figures

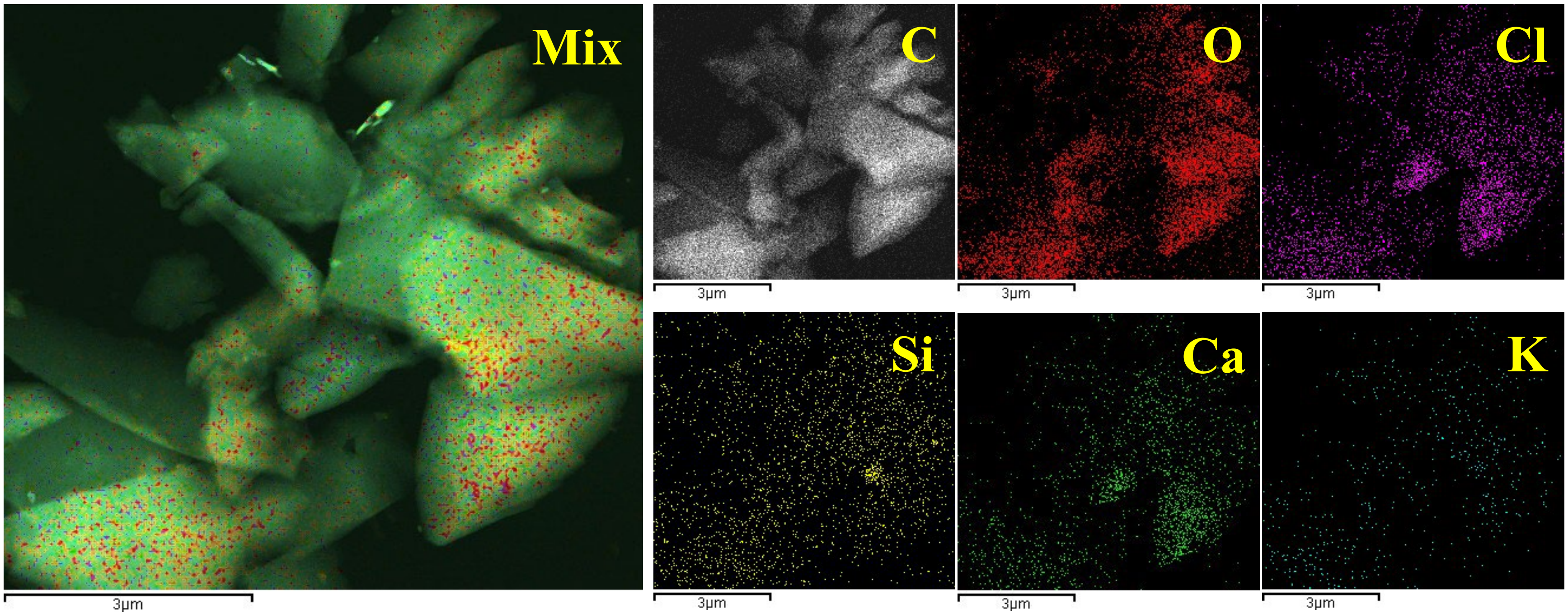

Fig. S1. TEM image and EDX elemental mapping of pristine SDBC.

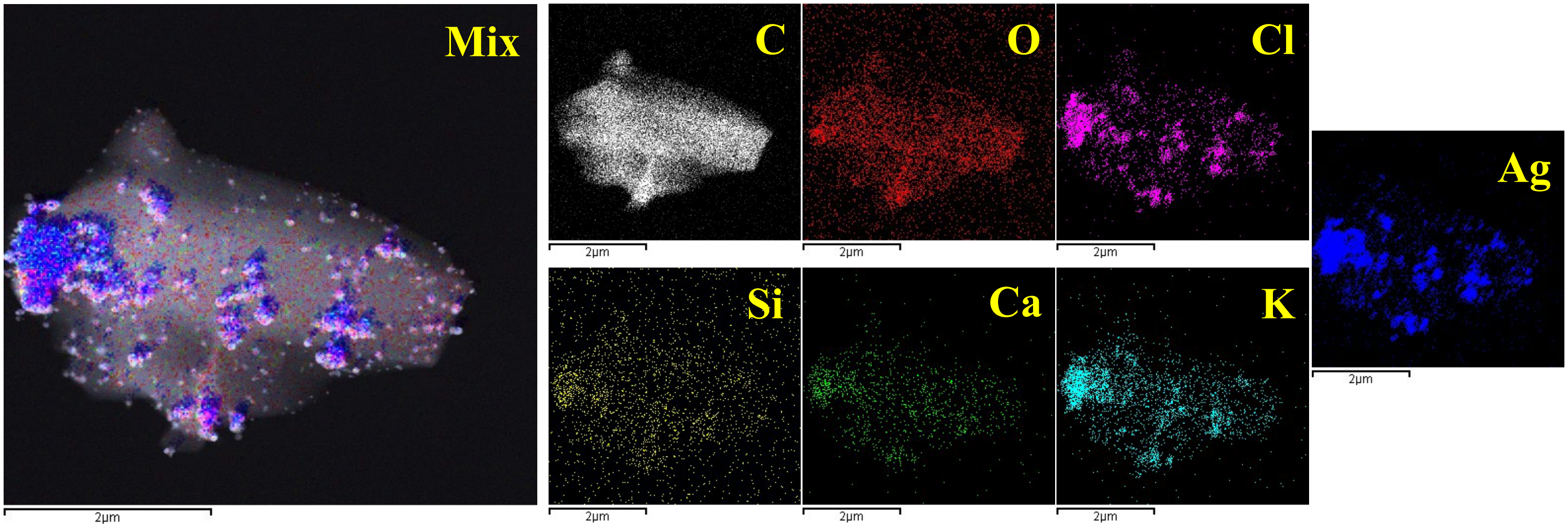

Fig. S2. Elemental mapping of Ag-SDBC by TEM–EDX analysis.

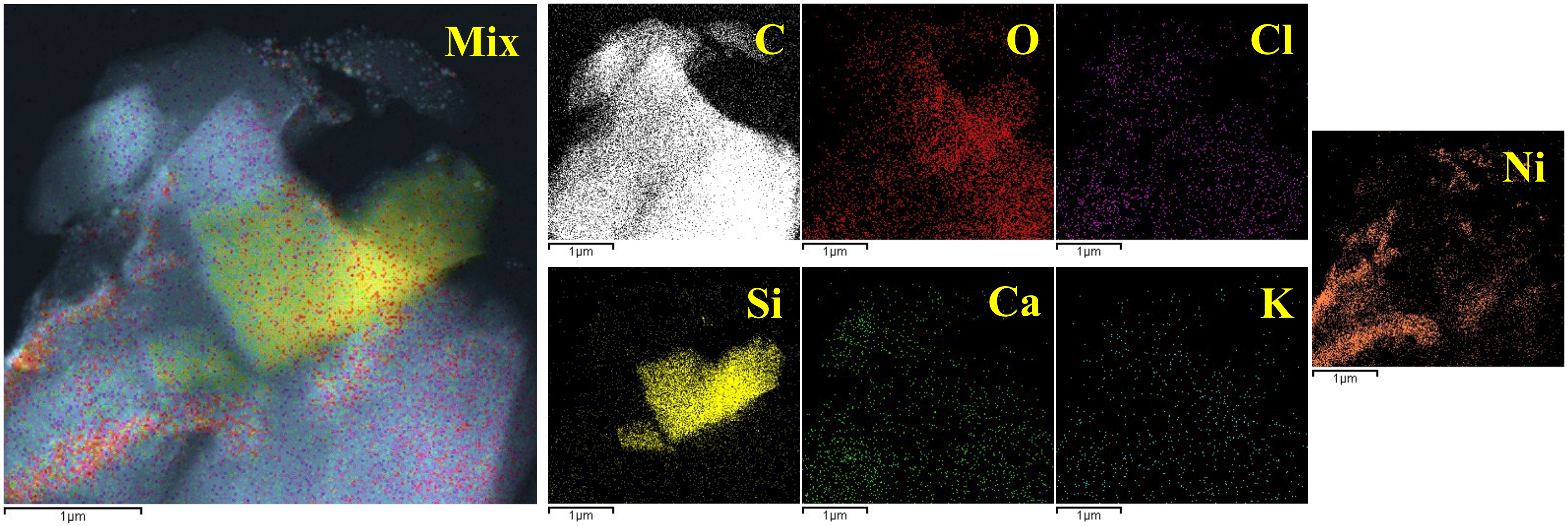

Fig. S3. TEM image and EDX elemental distribution maps of the Ni-SDBC catalyst.

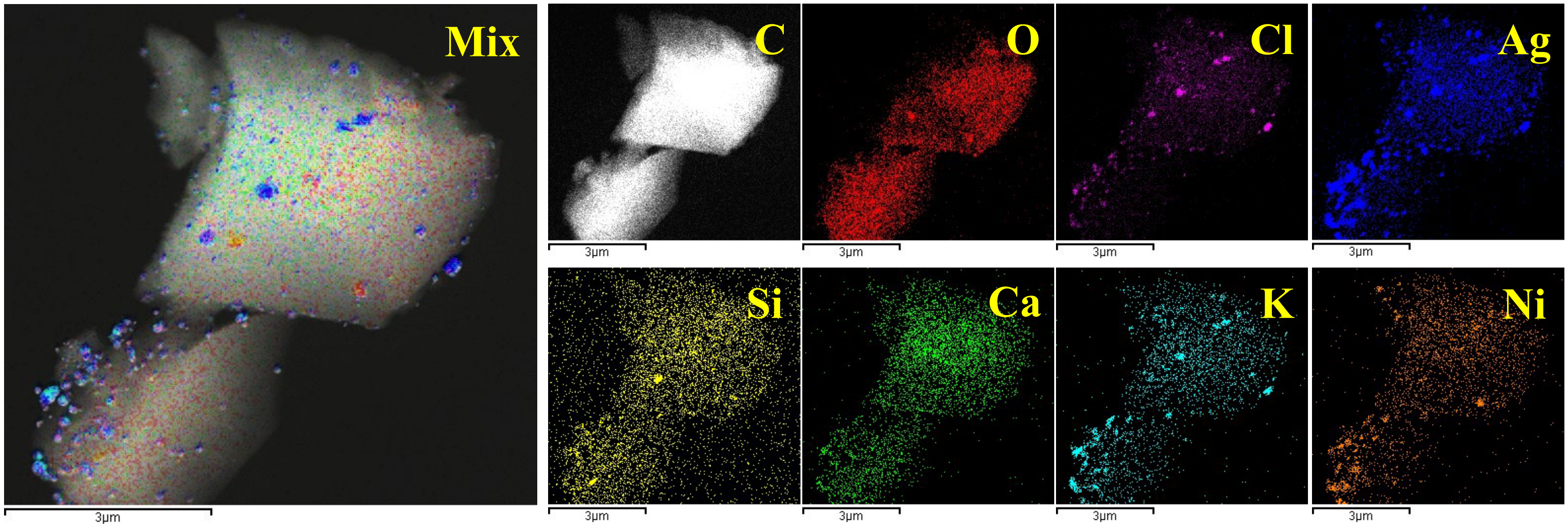

Fig. S4. TEM micrograph and corresponding EDX elemental maps of the bimetallic-loaded biochar (Ag/Ni-SDBC).

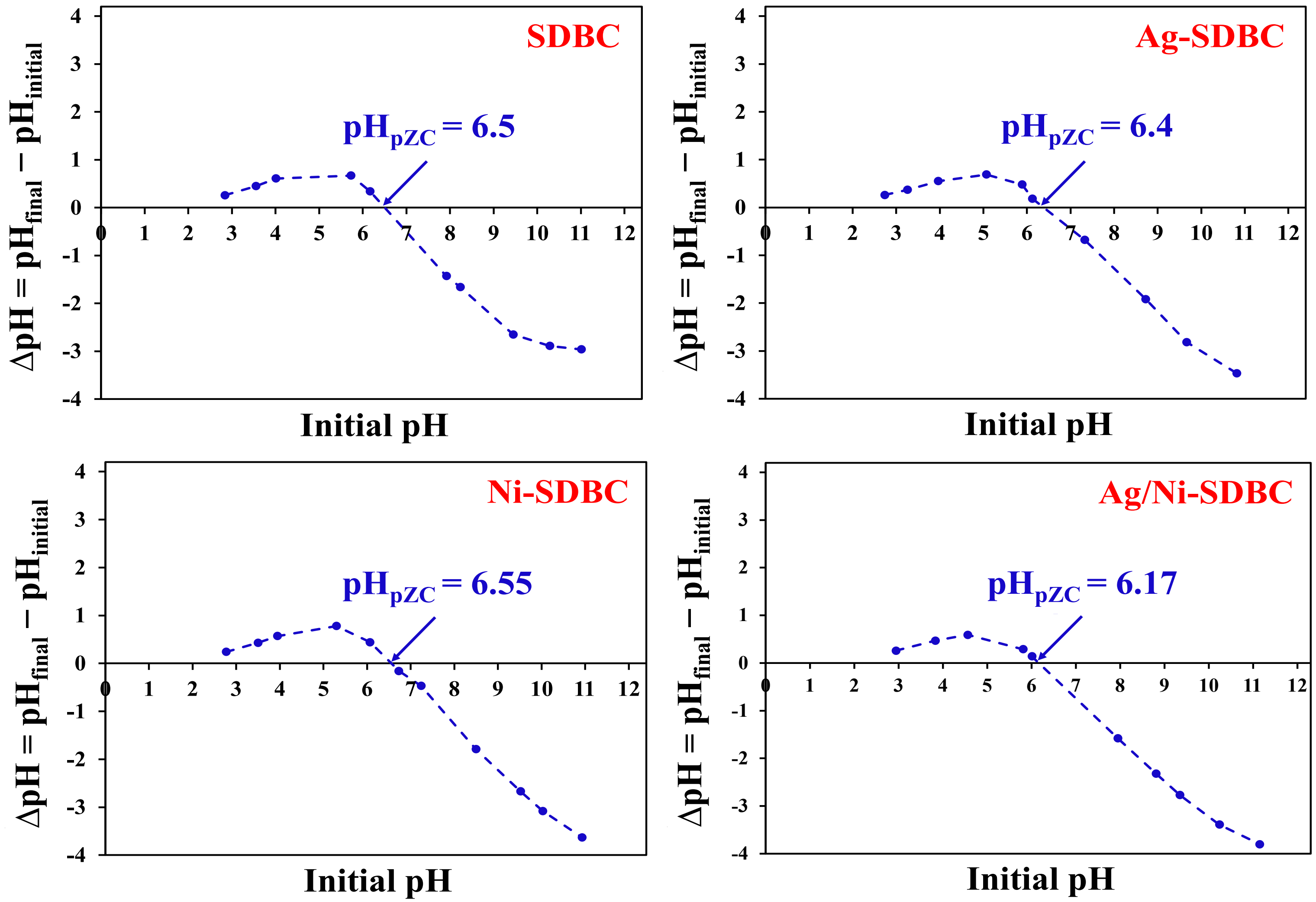

Fig. S5. pH_{pZC} values of pristine SDBC and the modified SDBC-based catalyst.

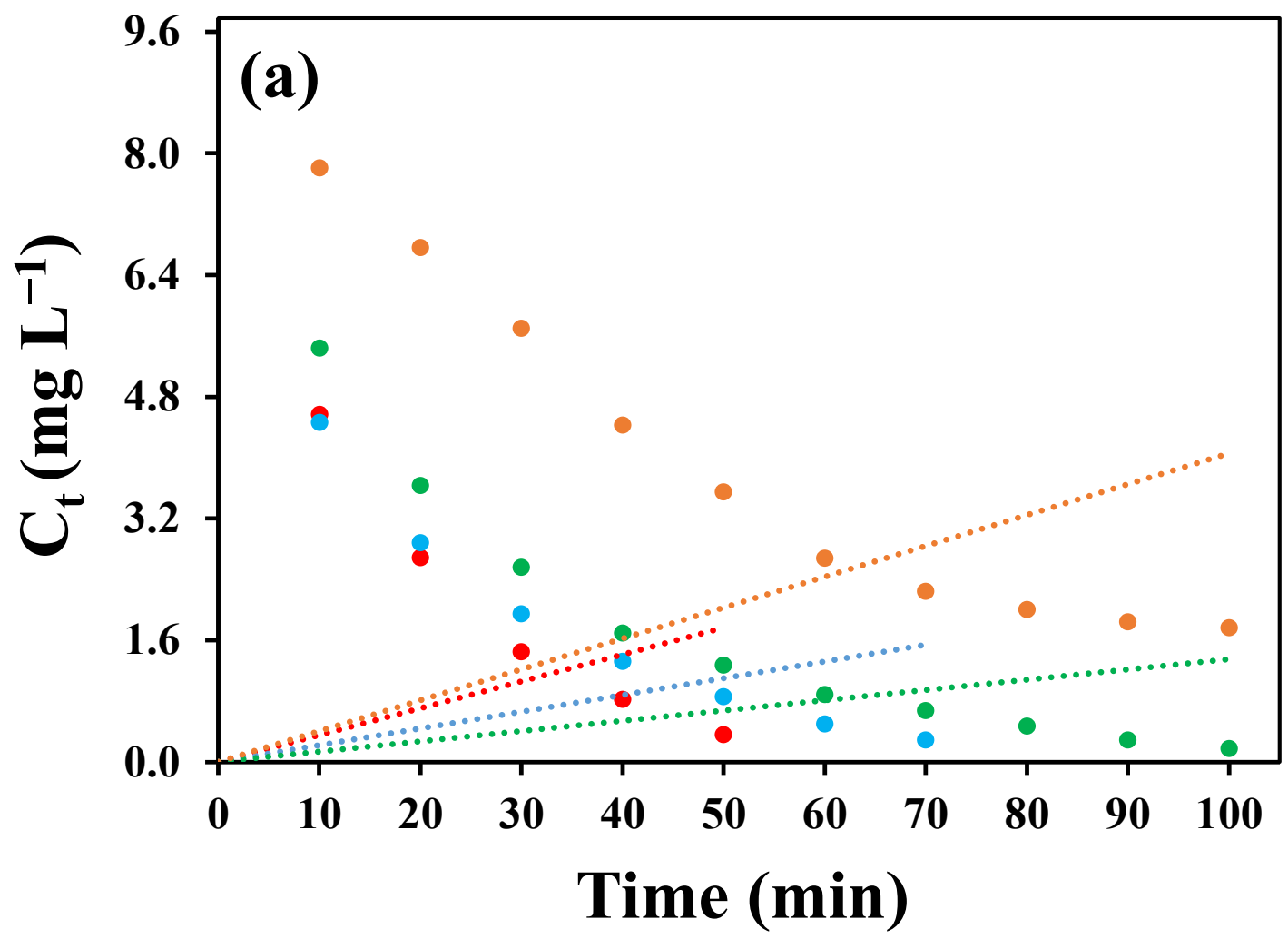

pH	k_0 (mg L^{-1} min^{-1})	R^2
pH = 3	0.0353	0.0523
pH = 5	0.022	0.0501
pH = 7	0.0135	0.0449
pH = 9	0.0405	0.2149

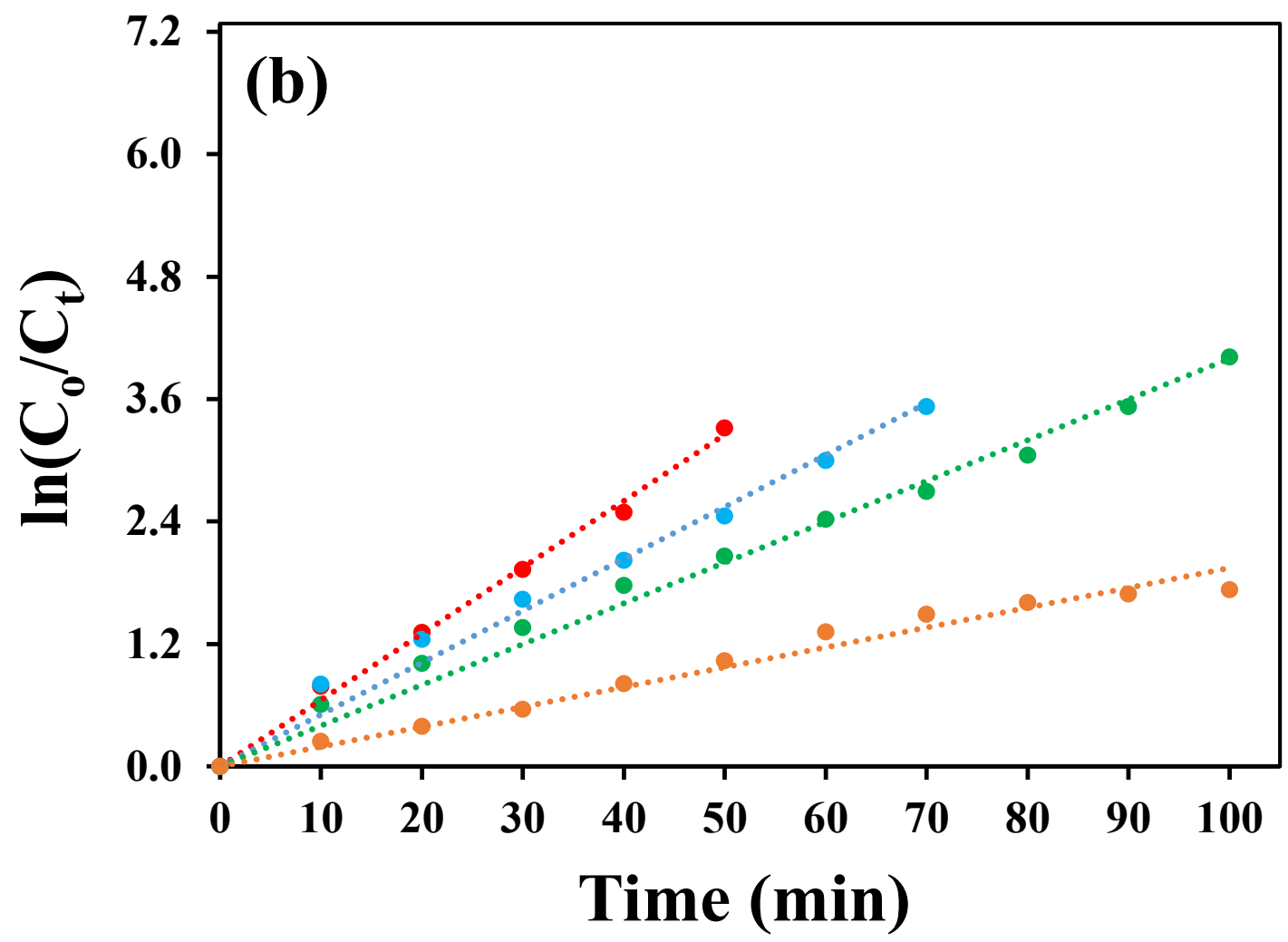

pH	k_1 (min^{-1})	R^2
pH = 3	0.065	0.9985
pH = 5	0.0509	0.9955
pH = 7	0.04	0.997
pH = 9	0.0195	0.9932

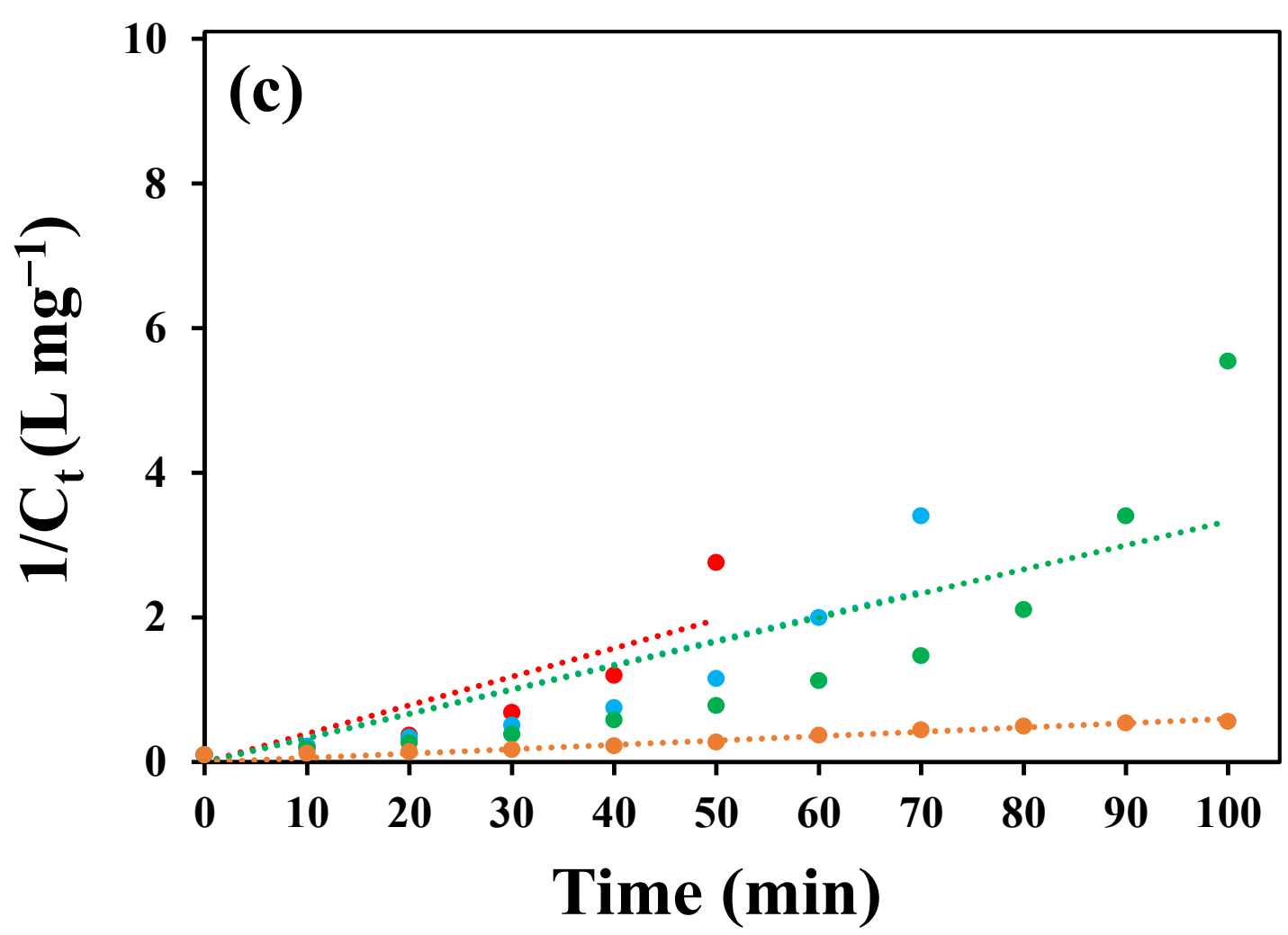

pH	k_2 (L mg^{-1} min^{-1})	R^2
pH = 3	0.0394	0.8715
pH = 5	0.0337	0.8828
pH = 7	0.0333	0.8302
pH = 9	0.006	0.987

Fig. S6. Comparison of the kinetic rate constants for LEV degradation in the Ag/Ni-SDBC/PI system under different pH conditions based on (a) zero-order, (b) pseudo-first-order, and (c) pseudo-second-order kinetic models. Experimental conditions: $[LEV]_o$ = 10 mg L^{-1}, $[PI]_o$ = 2 mM, $[Ag/Ni\text{-}SDBC]_o$ = 0.5 g L^{-1}, T = 25 °C, and reaction time = 100 min.

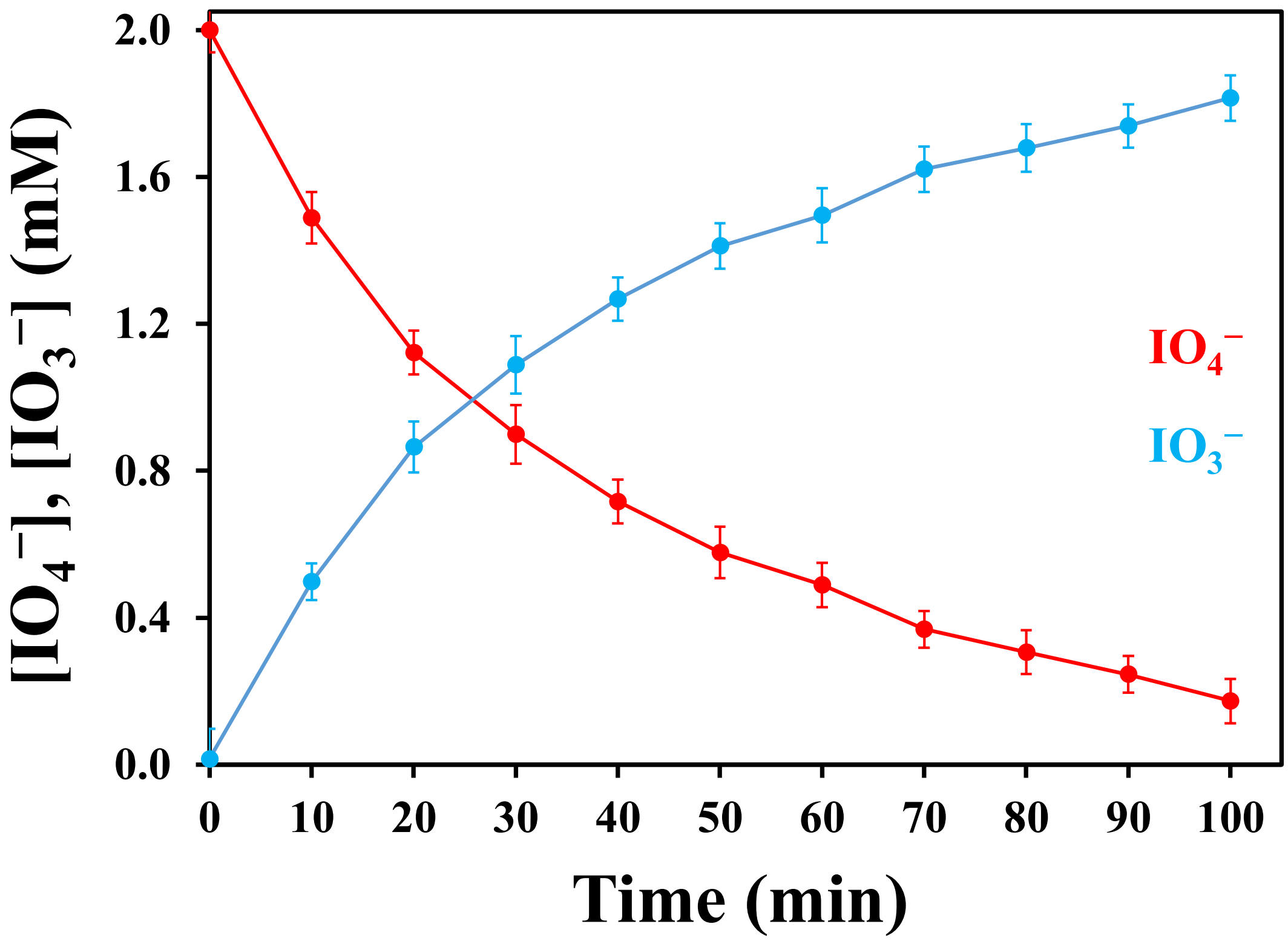

Fig. S7. Concentration profiles of IO_4^- and IO_3^- during LEV degradation in the Ag/Ni-SDBC/PI system. Conditions: $[LEV]_o$ = 10 mg L^{-1}, $[PI]_o$ = 2 mM, $[Ag/Ni\text{-}SDBC]_o$ = 0.5 g L^{-1}, pH = 7, T = 25 °C, and reaction time = 100 min.

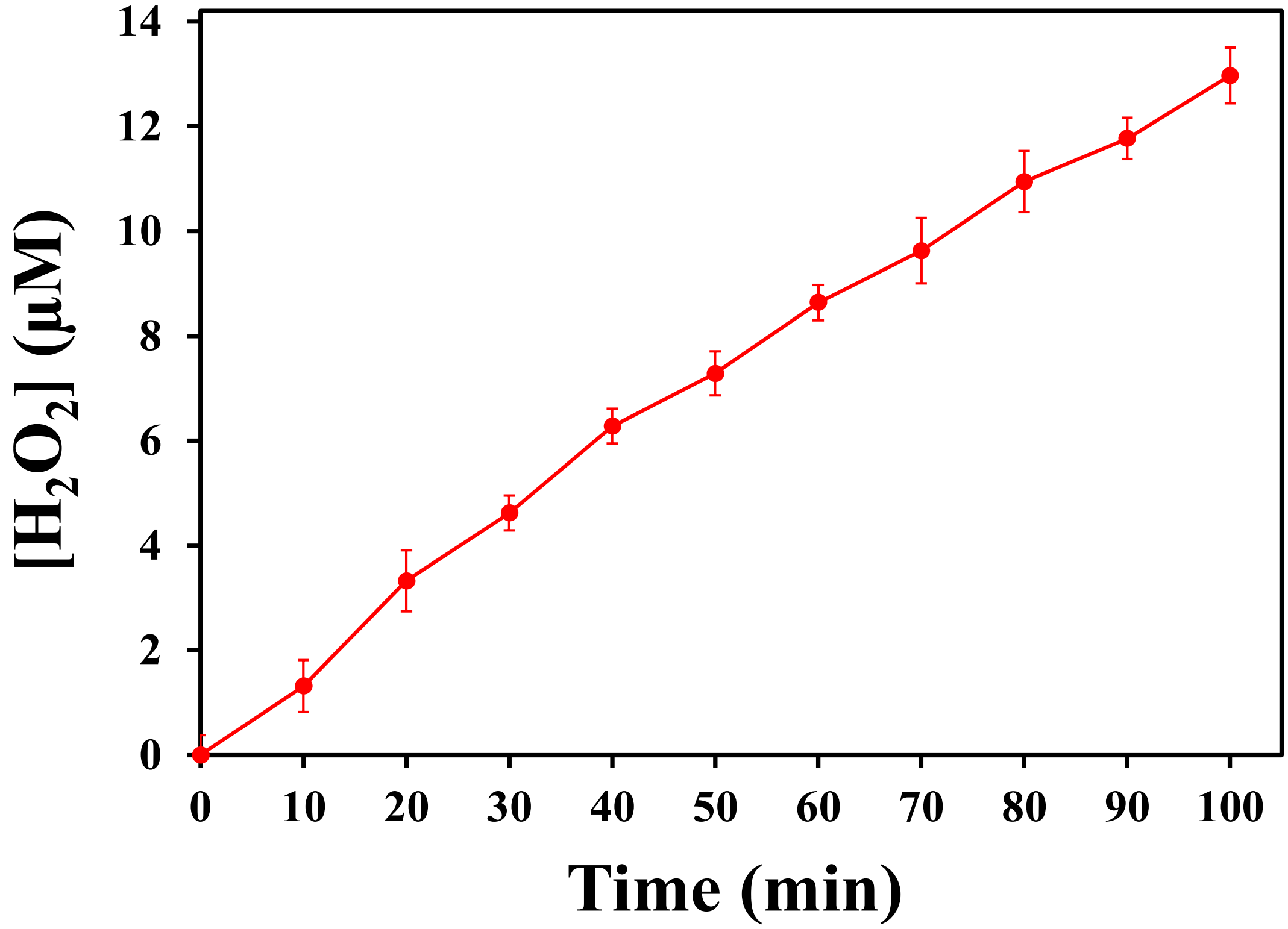

Fig. S8. H_2O_2 generation by the Ag/Ni-SDBC system in deionized water. Conditions: Deionized water = 100 mL, $[Ag/Ni\text{-}SDBC]_o = 0.5$ g L^{-1}, pH = 7, T = 25 °C, and reaction time = 100 min.

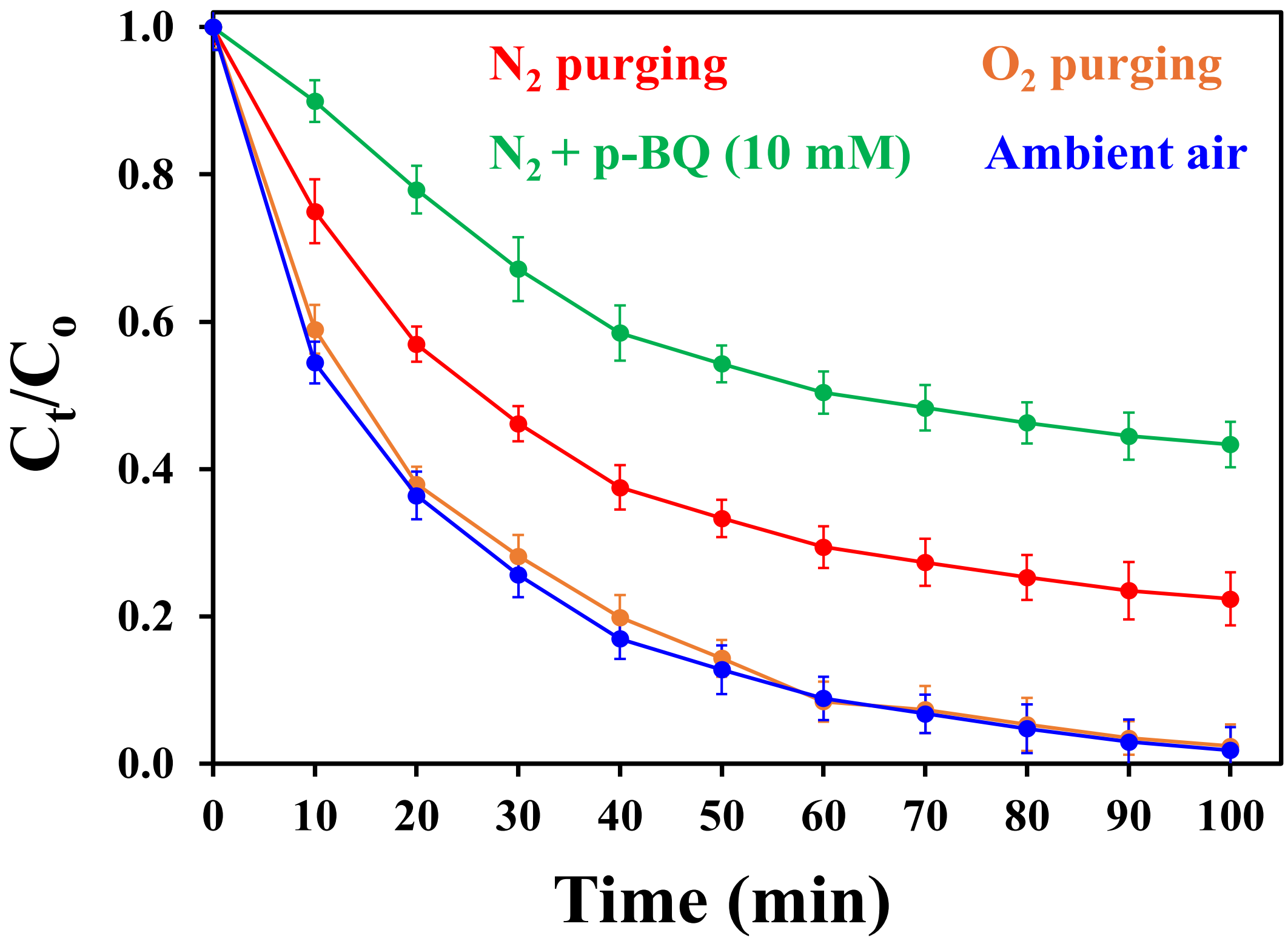

Fig. S9. Effects of different gaseous atmospheres on LEV degradation in the Ag/Ni-SDBC/PI system. Experimental conditions: $[LEV]_o = 10$ mg L^{-1}, $[PI]_o = 2$ mM, $[Ag/Ni\text{-}SDBC]_o = 0.5$ g L^{-1}, pH = 7, T = 25 °C, and reaction time = 100 min.

References

1 F. Ashraphy, M. Hasan, M. A. Alam, M. A. Akbor, M. S. Quddus and S. Kamruzzaman, Desalination Water Treat., 2025, 323, 101409.

2 M. M. Gaber, H. Shokry, A. H. Hassanin, S. Awad, M. Samy and M. Elkady, Applied Water Science 2025 15:2, 2025, 15, 32-.

3 B. E. Alvarez-Serna, T. A. García-Mejía, S. Arzate, A. Yañez-Aulestia, R. G. Ramírez-Chavarría and R. M. Ramírez-Zamora, Electroanalysis, 2025, 37, e202400096.

4 S. S. Saleh, H. M. Lotfy and H. T. Elbalkiny, Environ. Sci. Process. Impacts, 2023, 25, 2125–2138.

5 Y. Wang, J. Li, L. Ji and L. Chen, Molecules, 2022, 27, 2160.

6 E. Butovskaya, A. M. Carrillo Heredero, G. Segato, E. Faggionato, M. Borgia, D. Marchis, S. Menotta and S. Bertini, Food Additives & Contaminants: Part A, 2024, 41, 601–609.

7 R. Zhang, C. Peng, Q. Wang, X. Zou, Q. Zhao, J. Wang, J. Zhang, L. Dong, X. Zhang and N. Gao, Environ. Sci. (Camb)., 2023, 9, 2041–2052.

8 H. Sun, F. He and W. Choi, Environ. Sci. Technol., 2020, 54, 6427–6437.

9 K. Zhang, S. Zhang, C. Ye, R. Ou, H. Zeng, X. Yu and M. Feng, Chemical Engineering Journal, 2023, 451, 138642.

10 Y. Xiong, X. Tang, Y. Liu, W. Li, Y. He, Y. Deng, Z. Lin and Y. Zhou, Sep. Purif. Technol., 2024, 333, 125813.

11 Y. Bichsel and U. von Gunten, Environ. Sci. Technol., 2000, 34, 2784–2791.

12 K. Zhang, C. Ye, Y. Lou, X. Yu and M. Feng, J. Hazard. Mater., DOI:10.1016/J.JHAZMAT.2022.130058.

13 X. Wang, P. Zhang, S. Meng, C. Zhou, Z. Xiong, C. He, Y. Liu, H. Zhang, P. Zhou and B. Lai, Sep. Purif. Technol., 2025, 354, 129186.

14 L. Chen, H. Huang, X. Zhang, W. Tang, J. He and Y. Yang, Journal of Water Process Engineering, 2024, 65, 105879.

15 M. M. Gaber, M. Samy, E. A. El-Bestawy and H. Shokry, Chemosphere, 2024, 352, 141448.

16 L. Niu, G. Zhang, G. Xian, Z. Ren, T. Wei, Q. Li, Y. Zhang and Z. Zou, Sep. Purif. Technol., 2021, 259, 118156.

17 M. Wang, Y. Zhou, S. Yang, X. Jiang, X. Jiang, Z. Xing and Y. Guan, Water (Switzerland), 2023, 15, 2190.

18 Standard methods for the examination of water and waste water, American Public Health Association (APHA), American Water Works Association (AWWA), Water Environment Federation (WEF), Washington, DC, 23rd edn., 2017.

19 W. Duan, M. Zhang and R. Zhou, Water Science and Technology, 2024, 89, 212–224.

20 H. Zeng, Y. Chen, J. Xu, S. Li, J. Wu, D. Li and J. Zhang, Chemical Engineering Journal, 2024, 482, 148885.

21 W. Zou, M. Zhang, S. Chang, Z. Wang, D. Zhang, J. Huo, A. Peng, X. Zhang, W. Guo and H. H. Ngo, J. Environ. Chem. Eng., 2025, 13, 118226.

22 A. Eslami, F. Mehdipour, R. Feizi, F. Ghanbari, K. Y. A. Lin, A. Bagheri and S. Madihi-Bidgoli, Korean Journal of Chemical Engineering 2023 40:4, 2023, 40, 882–891.

23 F. Hayati, A. A. Isari, B. Anvaripour, M. Fattahi and B. Kakavandi, Chemical Engineering Journal, 2020, 381, 122636.

24 J. Song, H. Wei, R. Teng, Z. Zhang, F. Pan, Z. Zhang and M. Gao, Environ. Res., 2026, 289, 123415.

25 M. Khajeh, M. M. Amin, A. Fatehizadeh and T. M. Aminabhavi, Chemical Engineering Journal, 2021, 416, 129163.

26 Q. Jiang, S. Jiang, H. Li, R. Zhang, Z. Jiang and Y. Zhang, Chemical Engineering Journal, 2022, 431, 133937.
